# Short-term exposure to antibiotics begets long-term disturbance in gut microbial metabolism and molecular ecological networks

**DOI:** 10.1186/s40168-024-01795-z

**Published:** 2024-05-07

**Authors:** Yuehui Hong, Hao Li, Linkang Chen, Hongtian Su, Bin Zhang, Yu Luo, Chengji Li, Zuguo Zhao, Yiming Shao, Lianxian Guo

**Affiliations:** 1 Dongguan Key Laboratory of Public Health Laboratory Science, The First Dongguan Affiliated Hospital, School of Public Health, Guangdong Medical University, Dongguan, 523808 China; 2 Jiangmen Key Laboratory of Traditional Chinese Medicine Ingredients and Their Mechanisms of Action, Guangdong Jiangmen Chinese Medicine College, Jiangmen, 529000 China; 3 Dongguan Key Laboratory of Sepsis Translational Medicine, The First Dongguan Affiliated Hospital, Guangdong Medical University, Dongguan, 523808 China

**Keywords:** Antibiotic, Molecular ecological network, Gut microbiota, Microbial diversity, Metabolomics, Metagenomics

## Abstract

**Background:**

Antibiotic exposure can occur in medical settings and from environmental sources. Long-term effects of brief antibiotic exposure in early life are largely unknown.

**Results:**

Post a short-term treatment by ceftriaxone to C57BL/6 mice in early life, a 14-month observation was performed using 16S rRNA gene-sequencing technique, metabolomics analysis, and metagenomics analysis on the effects of ceftriaxone exposure. Firstly, the results showed that antibiotic pre-treatment significantly disturbed gut microbial α and β diversities (*P* < 0.05). Both Chao1 indices and Shannon indices manifested recovery trends over time, but they didn’t entirely recover to the baseline of control throughout the experiment. Secondly, antibiotic pre-treatment reduced the complexity of gut molecular ecological networks (MENs). Various network parameters were affected and manifested recovery trends over time with different degrees, such as nodes (*P* < 0.001, *R*^2^ = 0.6563), links (*P* < 0.01, *R*^2^ = 0.4543), number of modules (*P* = 0.0672, *R*^2^ = 0.2523), relative modularity (*P* = 0.6714, *R*^2^ = 0.0155), number of keystones (*P* = 0.1003, *R*^2^ = 0.2090), robustness_random (*P* = 0.79, *R*^2^ = 0.0063), and vulnerability (*P* = 0.0528, *R*^2^ = 0.28). The network parameters didn't entirely recover. Antibiotic exposure obviously reduced the number of key species in gut MENs. Interestingly, new keystones appeared during the recovery process of network complexity. Changes in network stability might be caused by variations in network complexity, which supports the ecological theory that complexity begets stability. Besides, the metabolism profiles of the antibiotic group and control were significantly different. Correlation analysis showed that antibiotic-induced differences in gut microbial metabolism were related to MEN changes. Antibiotic exposure also caused long-term effects on gut microbial functional networks in mice.

**Conclusions:**

These results suggest that short-term antibiotic exposure in early life will cause long-term negative impacts on gut microbial diversity, MENs, and microbial metabolism. Therefore, great concern should be raised about children’s brief exposure to antibiotics if the results observed in mice are applicable to humans.

Video Abstract

**Supplementary Information:**

The online version contains supplementary material available at 10.1186/s40168-024-01795-z.

## Introduction

Antibiotics have saved many lives of patients who would have otherwise died from infections. It is therefore not surprising that antibiotics are one of the most commonly prescribed medicines to patients with infectious diseases [1], including infants and children. In addition, antibiotics are commonly used in pet animals and animal husbandry for prophylactic and therapeutic reasons and also as growth promoters [2]. They are also applied as pesticides in agriculture [3, 4]. However, the extensive use of antibiotics has led to their regular and repeated release into the environment. An inevitable negative impact of antibiotic use is the emergence and dissemination of drug-resistant bacteria and resistance genes [2]. Antimicrobial resistance is a serious worldwide problem for both public and animal health [5, 6]. Antibiotic resistance has now been escalated by major world health organizations to one of the top health challenges facing the twenty-first century [7]. It had been demonstrated that farms using antimicrobial growth promotants (AGPs) had more resistant bacteria in the gut floras of the farm workers and farm animals than in those on farms not using AGPs [7]. Food may act as a vector for the transmission of resistant bacteria and resistance genes to humans [8], since food is easily contaminated by resistant bacteria and resistance genes in several ways, such as during animal slaughter or food processing. When the contaminated food is ingested, the bacteria may colonize humans or transfer resistance material to other bacteria belonging to the endogenous human flora, leading to negative effects. For instance, it has been shown that pork and poultry meat can both be sources of transfer of resistant strains and genes to humans [8, 9].

One of the main promoting factors for antimicrobial resistance is the antibiotic use for human health and problems with sanitation [10]. Hospitals are a major source for the release and spread of antibiotic-resistant bacteria in the environment. Great concerns have been raised because hospital effluent is generally discharged untreated into the main wastewater system and eventually into the environment, which may lead to antibiotic pollution. Besides, a close correlation between antibiotic use and the development of individual and community-level bacterial resistance has been verified [11].

Antibiotics are administered to over 10% of European children yearly [12] and are one of the most commonly used drugs in Chinese children. However, great concerns have been raised regarding the negative impacts of antibiotic exposure on human health, since researches have shown that antibiotic use is associated with gut microbial dysbiosis, asthma, inflammatory bowel disease (IBD), allergy, obesity, and diabetes [13]. Gut microbiota plays important roles in regulating the human immune system, in metabolism, and in hormone secretion and responses [14, 15]. It has been well-documented that antibiotic use is related to the disorder of the gut microbiome, which may cause various diseases [13]. This is particularly noticeable in children, as their gut microbiome is more susceptible to the effects of antibiotics. In fact, it has been demonstrated that children’s exposure to antibiotics is related to an increased risk for excessive weight gain, asthma, allergies, and autoimmune diseases [16, 17]. Besides, since animal models have verified that gut microbiota plays a role in the development of brain structure and function, great concerns have also been raised about the potential adverse impacts of antibiotics on child brain development [18, 19]. Besides antibiotic exposure from medical settings, antibiotics from environmental sources are also an exposure risk (as mentioned above) [20].

Studies have shown that the overuse and misuse of antibiotics in animal husbandry and medicine have increased the abundance of antibiotic resistance bacteria and genes in human-associated environments [11]. Many researches have focused on antibiotic resistance, whereas investigating the long-term negative effects of antibiotic exposure in early life is also an important topic. Although it has been shown that even brief antibiotic exposure can cause long-term effects on microbiota composition, little is known about the following points: (1) the long-term effects of antibiotic exposure in early life on gut microbial metabolism; (2) whether and how antibiotic exposure in early life exhibits long-term effects on the ecological networks in gut microbial communities; (3) whether network complexity in gut microbiota is related to network stability.

**Fig. 1:**
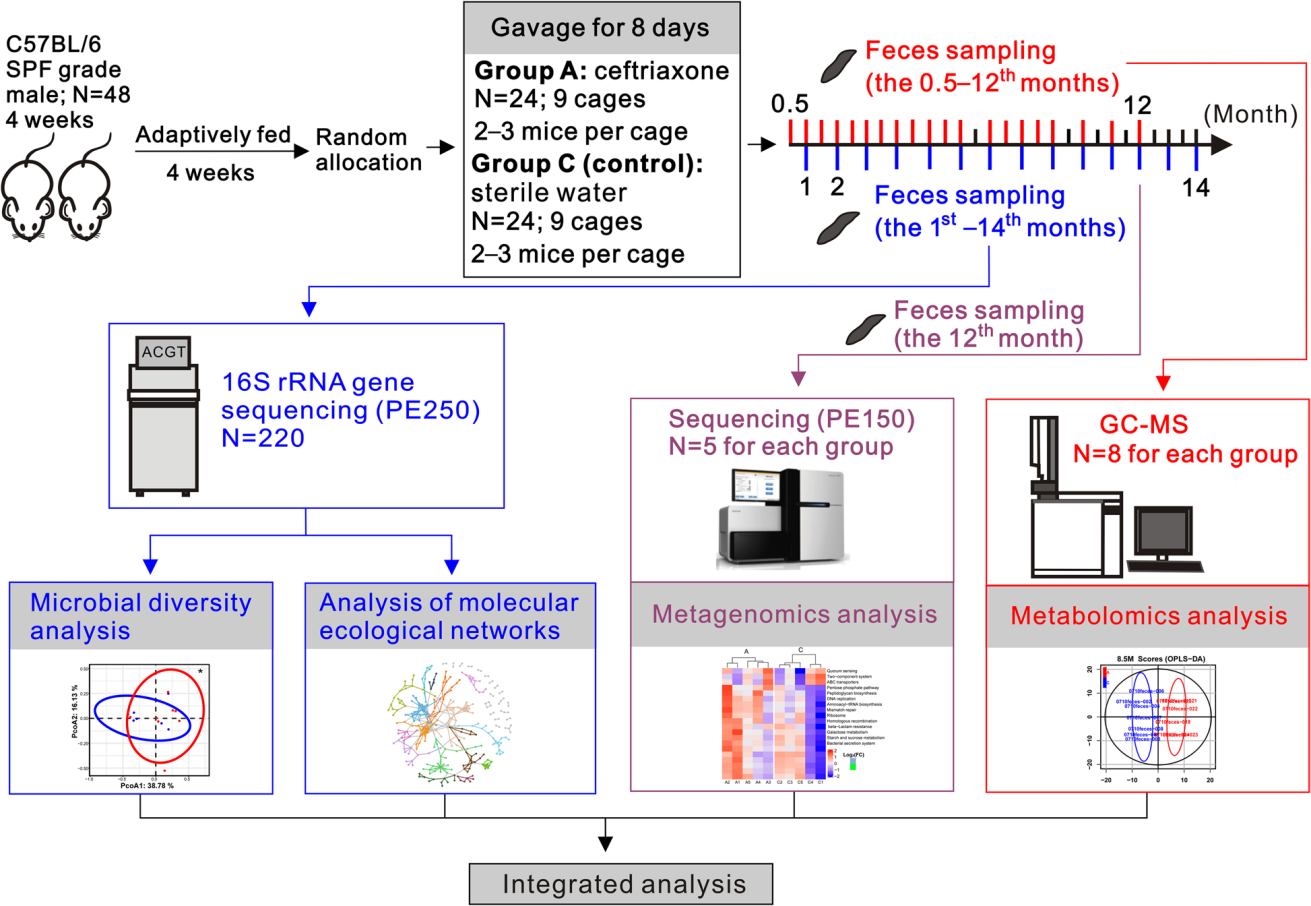
Schematic overview of the experimental design. N represents the sample size. PE means the paired-end sequencing mode. SPF means specific pathogen-free. Fecal samples from the 1st–14th months post the cessation of antibiotic treatment were used for 16S rRNA gene sequencing. Fecal samples from the 12th month and 0.5–12th months were detected by metagenomics analysis and metabolomics analysis, respectively

In this study, to investigate whether and how early-life antibiotic exposure exhibits long-term effects on the ecological networks and metabolism of the gut microbiota, we conducted a longitudinal study spanning 14 months to examine the temporal dynamics of gut microbial networks and fecal microbial metabolism post a short-term oral administration of antibiotics in C57BL/6 mice with 8 weeks of age (Fig. 1). The results may provide a deep insight into the long-term negative effects of antibiotic exposure in early life and provide guidance for treatment of disorders and diseases caused by antibiotic exposure.

## Materials and methods

### Animal experiment

This work received approval for research ethics from the Animal Care and Use Committee of the Laboratory Animal Science Institute of Guangdong Medical University (Additional file 1). Male C57BL/6 mice (approximately 16 g) at 4 weeks of age (SPF grade) were purchased from the Guangdong Medical Experimental Animal Center. The mice were treated humanely with efforts to minimize suffering. A schematic overview of the experimental design is shown in Fig. 1. A total of 48 mice were adaptively fed for 4 weeks prior to experiment treatment in 8 static microisolator cages supplemented with autoclaved padding under conditions of 22 ℃, 40–70% humidity, and a 12/12-h light/dark cycle. Then, the mice were randomly divided into two groups (antibiotic group (named group A); and control (named group C)), with 24 mice in each group (9 cages per group; 2–3 mice per cage; the mice had their own number and were kept in fixed cages to avoid intra-group and inter-group mixing). Mice in group A were continuously orally administered with 0.2 mL of ceftriaxone (400 mg/mL) [21–23] for 8 days, twice a day with an interval of 8 h between intragastric administrations. Mice in group C were treated with sterile water by gavage in the same manner. After treatment, the mice were kept in their previous cages. Fecal samples from the mice were collected in the following 14 months. Food and water were provided to the mice ad libitum throughout the experiment. Fecal pellets from mice were collected at least once every 2 weeks during the experiment. Feces from the mice were collected under aseptic conditions, immediately snap-frozen, and stored at − 80 ℃. The feces were used for microbial diversity analysis (samples from each month of the 1st–14th months), metabolomics analysis, and metagenomics analysis (samples from the 12th month). Fecal samples from 0.5 to 12th months (0.5, 1, 1.5, 2, 2.5, 3, 3.5, 4, 4.5, 5, 5.5, 6, 7, 7.5, 8, 8.5, 9, 10, 11, and 12 months) were used for the metabolomics analysis. Besides, the weight of the mice was measured during the whole experiment.

### Genomic DNA extraction, PCR, and Illumina sequencing

Genomic DNA from each feces sample was extracted with the PowerSoil^®^ DNA isolation kit (MoBio Laboratories, Carlsbad, CA, USA) according to the directions of manufacturers. After DNA quality verification, the V4-V5 region of the 16S rRNA gene was PCR amplified using primers 515F (5′-GTGCCAGCMGCCGCGGTAA-3′) and 907R (5′-CCGTCAATTCMTTTRAGTTT-3′). The primers were provided by Invitrogen (Carlsbad, CA, USA). PCR reaction mixtures contained 1 μL of each primer (10 μM), 25 μL of 2 × Premix Taq (Takara Biotechnology, Dalian Co., Ltd., Dalian, China), 3 μL of DNA (20 ng/μL), and sterile ddH_2_O to a total volume of 50 μL. PCR was carried out by the BioRad S1000 (Bio-Rad Laboratory, Hercules, CA, USA) using the following procedures: 94 ℃ for 5 min; 30 cycles of 94 ℃ for 30 s, 52 ℃ for 30 s, and 72 ℃ for 30 s; and 72 ℃ for 10 min. Triplicate reactions were performed for each sample, and the products were mixed, followed by purification using the EZNA Gel Extraction Kit (Omega Bio-Tek, Norcross, GA, USA). Sequencing libraries were prepared with the NEBNext^®^ Ultra™ DNA Library Prep Kit for Illumina^®^ (New England Biolabs, MA, USA) following the manufacturer’s directions. Paired-end sequencing (PE250) for 220 samples was performed using the Illumina Novaseq 6000 platform (Guangdong Magigene Biotechnology Co., Ltd., Guangzhou, China).

### Analysis of 16S rRNA gene-sequencing data

The obtained raw reads were processed by QIIME 2 (version 2021.2) [24]. After importing the raw data, the forward and reverse reads were truncated at 228 bases and 215 bases, respectively. Denoising and sequence combination were carried out with the DADA2 plugin [25], and sequences with a base quality score > 20 were retained. Taxonomic classification was performed using the Naïve Bayes classifier trained in the SILVA database. Unusual amplicon sequence variants (ASVs) with extremely low abundance were discarded, including the feature with a sum frequency of less than 10 at each time point and the feature appeared in less than 3 samples. Unclassified and contaminated ASVs were also filtered. Feature table, representative sequences, and species annotation were correspondingly combined. A total of 17,509,264 high-quality sequences were obtained. Rarefaction curves were analyzed. The feature table was rarefied at a depth of 37,800 sequences per sample. On the basis of the combined data, α- and β-diversity analyses were performed. Alluvial diagrams were created to visualize species composition across time. In β-diversity analysis, analysis of similarity (ANOSIM), multivariate analysis of variance (Adonis), and a multiple response permutation procedure (MRPP) were performed. Principal coordinate analysis (PCoA) and visualization were performed by the ggplot2 package based on the Bray–Curtis distance. Species difference analysis at the genus level was performed using the ALDEx2 tool. Data statistical analysis and result visualization were carried out using the R package (version 4.0.2) and GraphPad Prism 8 software. Besides, the functional potential of microbial communities was predicted based on the KEGG database using the Tax4fun2 in R package. Metabolic pathways with statistical differences were analyzed and visualized using the STAMP software.

### Construction of molecular ecological networks (MENs)

MEN analysis was based on the data of 16S rRNA gene sequencing. The MEN analysis was performed according to the methods previously described [26, 27]. MENs were constructed based on Pearson correlations of log-transformed ASV abundances, followed by a random matrix theory (RMT-)-based method, determining the correlation cut-off threshold [28]. The RMT was suitable for investigating the behaviors of various systems and MEN construction [29]. The RMT-based network method manifested obvious advantages as previously described [26]. For instance, this approach possessed a firm theoretical basis, since it was on the basis of two universal laws of RMT [26]. It can avoid arbitrary cut-off determination, which is a serious flaw in association network construction. Using the RMT-based network method can reduce the uncertainty in network construction and comparison [26]. The analysis tool of the RMT-based network method is called Molecular Ecological Network Analysis Pipeline (MENAP), which is usable from the Institute for Environmental Genomics, University of Oklahoma (http://ieg4.rccc.ou.edu/MENA/). To ensure the reliability of correlation analysis in this study, only the ASVs present at least in half of the samples were used for correlation calculation.

### Analysis of MEN parameters

The analysis methods of various network parameters referred to the approaches previously described in detail [26, 27]. The MEN indices were analyzed via the MENAP pipeline. The analyzed parameters included nodes, links, average degree (average K), average clustering coefficient (average CC), connectedness (Con), average path distance (GD), positive links, positive/negative ratios, number of modules, number of small modules, number of large modules, number of nodes in large modules, relative modularity (RM), number of keystones, vulnerability, and robustness. Connectors, module hubs, and network hubs were regarded as keystones [30]. To examine how each network parameter changed with time, regression analysis was performed between each network parameter and time (in months). Vulnerability and robustness were applied to characterize the stability of MENs. Robustness is the proportion of the remaining species in a network post random or targeted node removal [26, 31]. To simulate random species removal, 50% of nodes were randomly removed (robustness_random_removal). To simulate targeted removal, all module hubs were removed (robustness_targeted_removal). The proportion of residue nodes was regarded as the network’s robustness. The vulnerability of a node was used to determine the relative contribution of the node to the global efficiency [26]. Efficiency in ecological networks can indicate the speed of information transmitted to parts or the whole network.

### Preparation of fecal samples for metabolomics analysis

Fresh fecal samples (50 mg each) were placed in 1.5 mL centrifuge tubes. Eight samples were analyzed for each group at each sampling time. A total of 300 μL of purified water was added to every tube, followed by ultrasonic extraction for 5 min and vortex for 30 s. Then, the tubes were subjected to centrifugation (at 4 ℃) at 13,000 rpm for 15 min. Two hundred microliters of supernatant was pipetted. After discarding the residual water in the centrifuge tubes, 300 μL of methanol was added to every tube for ultrasonic extraction for 5 min, followed by vortex for 30 s. The tubes were subjected to centrifugation again (at 4 ℃) at 13,000 rpm for 15 min. Thereafter, 200 μL of supernatant was pipetted and combined with the previous supernatant in an injection vial, followed by evaporation dryness using nitrogen. After adding 80 μL of methoxyamine hydrochloride pyridine solution (20 mg/mL), the mixture was placed in an oven at 80 ℃ for 30 min. After natural cooling, 100 μL of BSTFA-TMCS was added, followed by a reaction in the oven at 70 ℃ for 2 h. Then, 150 μL of n-heptane solution containing 0.1 g/L n-docosane as internal standard was applied to terminate the reaction, followed by centrifugation at 13,000 rpm for 5 min. The resulting supernatant was used for GC–MS metabolomics analysis. Quality control (QC) sample was prepared by mixing an equal volume (50 μL) of the extract from every fecal sample. Then, the mixture (the same volume as that of other samples) served as the QC and was operated in the same way as other samples.

### Acquisition of GC–MS data

The following procedure was performed according to the methods previously described [32], with some modifications. The samples were analyzed via the Gas Chromatography Mass Spectrometer (7890B/5975A GC–MS System, Agilent, CA, USA). A DB-5MS UI capillary column coated with 5% phenyl methyl silox (Agilent J & W Scientific, Folsom, CA, USA) was used in the GC. One microliter of the samples was injected into the instrument at a split ratio of 10:1. Helium was used for carrier gas with a constant flow rate of 1 mL/min. The temperatures of injection, transfer line, ion source, and quadrupole were set at 280 ℃, 280 ℃, 230 ℃, and 150 ℃, respectively. The initial temperature program was set as isothermal heating (70 ℃) for 2 min, followed by increasing to 300 ℃ at a rate of 10 ℃/min. The final temperature was kept for 5 min. The solvent was delayed for 4 min. Electron impact ion source (EI) was employed with an electron energy of 70 eV. The full scan mode (SCAN) was used for data acquisition with a mass scanning range of 50–650 m/z.

### Analysis of GC–MS-based metabolomics data

The GC–MS data in [.D] format were transformed into “.abf” format with the AbfConvert (AnalysisBaseFileConverter tool). The retention index for all the compounds present in the metabolomic profile was calculated. The calculation was performed based on a calibration file, containing retention time and retention index values of selected 13 compounds (Fames) present in every sample. Then, the data were preprocessed, cleaned, deconvoluted, and aligned via the Automated Mass Spectral Deconvolution and Identification System (AMDIS, National Institute of Standards and Technology, USA) interface to match against the Mass Spectral and Retention Time Index (RI) library in the Fiehnlib Metabolome Database. Metabolites were further analyzed by comparing fragmentation patterns present in the Fiehnlib database. Peak seeking and quantification of selective ion traces were performed via the AMDIS. Generally, if a compound had an AMDIS match factor over 70%, a probability score larger than 20%, and a matching RI to a known compound, it was considered “probable”. The data, including metabolite names, the specific peak index (retention time), and peak areas, were imported into R software (version 4.0.2) for internal standard normalization. QC was corrected using the RSC algorithm. Missing values were supplemented using the Random Forest algorithm, and the data were log_2_ transformed.

### Bioinformatics analysis of metabolomics data

After the raw data were pretreated, OPLS-DA was performed using the ropls package in R software (version 4.0.2). Values of variable importance in the projection (VIP) were obtained. Data from QC samples were removed, followed by a comparison between groups. The *t*-test was adopted to determine statistical significance between the two groups. The criteria “fold change (FC) > 1, VIP > 1, and *P* < 0.05” were used to screen up-regulated differential metabolites. FC < 1, VIP > 1, and *P* < 0.05 were used to identify down-regulated differential metabolites. The differential metabolites were used for matching KEGG ID by the MetaboAnalyst 4.0 (https://www.metaboanalyst.ca/). Then, enrichment analysis was carried out in the KEGG database (organism group: bacteria). Visualization of differential metabolites and differential metabolic pathways was performed using R software (version 4.0.2). In addition, correlation analysis was carried out to examine the relation between network parameters and differential metabolic pathways.

### Metagenomics analysis

Genomic DNA from each feces sample in the 12th month was extracted using the ALFA-SEQ Advanced Soil DNA Kit (mCHIP BioTech Co., Ltd., Guangzhou, China) according to the directions of manufacturers. Following the detection of quantity and purity, the DNA sample was mixed with fragmentation buffer and subjected to random interruption using the ultrasonic cell disruptor. Then, sequencing libraries were constructed, followed by quality detection. The Illumina HiSeq 2500 platform was used for metagenomic sequencing (PE150). After base calling, the sequencing data were transformed into raw reads in the FASTQ files. The sequencing raw data were subjected to quality control using the Trimmomactic software [33]. Clean data were aligned to the host genome sequences using the BWA software (v0.7.17; -k 30; other parameters were default) [34] and filtered to exclude the reads from the mice. The remaining clean reads were de novo assembled using the MEGAHIT (https://github.com/voutcn/megahit; k-min 35, k-max 95, k-step 20). Residual reads in each sample were mixed and also assembled. After the assembly, scaffolds were obtained. Scaftigs were obtained by trimming the sequences containing N in the scaffolds. The scaftigs with lengths over 500 bp were retained for further analysis. Prodigal [35] was adopted to predict the open reading frame (ORF). Gene clustering and elimination of redundancy were performed using Mmseqs [36]. A non-redundant gene catalog (Unigene) was obtained post selecting the longest sequence in each cluster as the representative sequence. Clean reads were aligned to the gene catalog using BBMap [37], followed by calculating the abundance of each Unigene in each sample. Unigenes in the non-redundant gene catalog were aligned to the NCBI-NR database for species annotation. MetaPhlAn2 [38] was also used for species annotation. Then, the results of species composition and abundance in each taxonomic hierarchy were obtained based on the species annotation and gene abundance table. The predicted gene sequences were aligned to the KEGG database for function annotation. The enrichment of KEGG pathways for each sample was analyzed. The Wilcoxon rank-sum test or *t*-test was adopted to analyze the differences between groups. ComplexHeatmap in the R package (version 4.2.1) was used for heatmap visualization.

### Functional network analysis

Functional network analysis was based on the data of 16S rRNA gene sequencing and metagenomic sequencing, respectively. For the former analysis, metabolic pathways were predicted based on the KEGG database and the Tax4fun2 in R package. Pearson correlation coefficients between pathways were calculated, and functional networks were constructed using pathways and the correlation as nodes and links, respectively. Core subnetworks were extracted using the MCODE plugin (degree cutoff: 2; K-core: 2; Max. depth: 100) in the Cytoscape software [39]. Finally, the networks were subjected to visualization using the Cytoscape. Functional network analysis of metagenomic sequencing data was based on KEGG annotation results. Pearson correlation coefficients between proteins/enzymes or pathways at the level_3 categories were calculated. Functional networks, unless specifically stated, were constructed using pathways and the correlation as nodes and edges (links), respectively. Core subnetworks were extracted using the MCODE plugin (degree cutoff: 2; K-core: 3; Max. depth: 100) in the Cytoscape. Network visualization was also performed using the Cytoscape.

## Results and discussion

### Effects of antibiotic exposure on the weight and gut microbial diversity in mice

As shown in Supplementary Figure S1, most time the average weight of mice in the antibiotic group seemed smaller than that of the control, whereas only days 0, 105, and 120 after the cessation of antibiotic treatment showed statistical differences. Interestingly, the weight of mice in the antibiotic group was somewhat greater than that of the control at the endpoint of 8-day antibiotic treatment (day 0). In terms of these data, it was hard to explain the underlying mechanisms of weight changes related to short-term exposure to ceftriaxone.

**Fig. 2:**
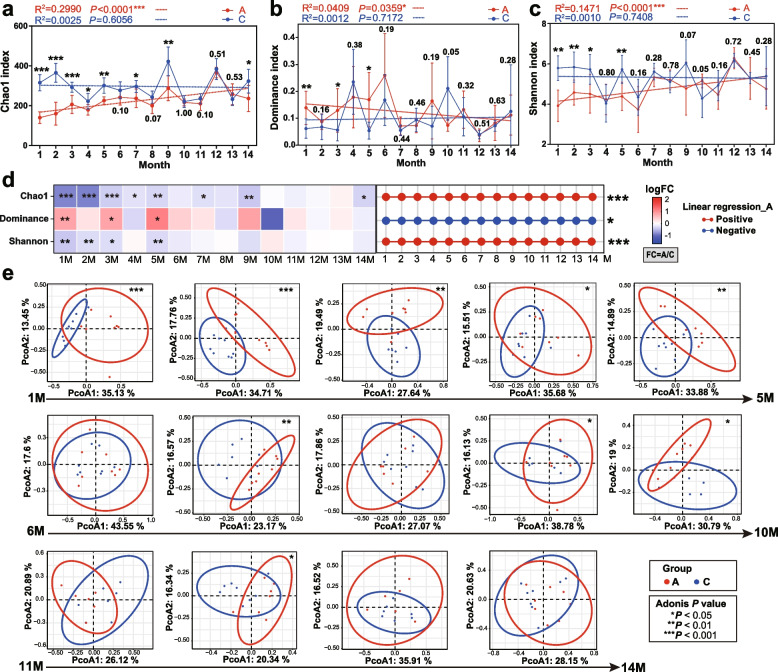
Results of α- and β-diversity analyses. The data were from 16S rRNA gene sequencing. M is short for month. Chao1 index, Dominance index, and Shannon index are shown in **a**, **b**, and **c**, respectively. Normality testing was performed using the Shapiro–Wilk test. For α-diversity, the nonparametric test was adopted due to that some samples were not in accordance with normal distribution. Wilcoxon test was performed for the comparison between the antibiotic group and control. Red and blue dotted lines indicate the regression of the antibiotic group and control, respectively. The corresponding *R*^2^ and *P* values are shown. Each number around the broken lines denotes the *P* value of statistical analysis between two groups at each time point. **d** Heatmaps showing the relative magnitude of α-diversity indices between antibiotic group (A) and control (C). The values are represented by logFC (FC = A/C). The right half part of **d** shows the results of regression analysis (for the antibiotic group) with α-diversity indices against time. Connections between red nodes indicate that the index increases over time (positive), while connections between blue nodes represent that the index decreases with time (negative). **e** PCoA analysis. The Bray–Curtis distance was used for β-diversity analysis. The *P* values were produced from Adonis analysis. The data on ASV abundances were used for the PCoA analysis. *P < 0.05; **P < 0.01; ***P < 0.001

The rarefaction curves in microbial diversity analysis are shown in Supplementary Figure S2, indicating enough sequencing depth. We used linear regression analysis to reveal the changes of three α-diversity indices across time (14 months). In the first few months, Chao1 indices and Shannon indices were significantly lower in the antibiotic group (Fig. 2a, c), indicating that antibiotic treatment resulted in reduced gut microbial diversity. In the antibiotic group Chao1 index and Shannon index showed an overall increasing trend over time, suggesting that the gut microbial diversity gradually recovered over time after cessation of antibiotic use. Both the Chao1 indices and Shannon indices from the antibiotic group in the 11th, 12th, and 13th months approached those of the control group. However, the diversity didn’t entirely recover, since the Chao1 indices between the two groups were still significantly different (*P* < 0.05) in the 14th month. Although the difference in Shannon indices between the two groups in the 14th month was not statistically significant, a minor difference was observed. The Dominance indices in the first 3 months of the ceftriaxone group were greater than those of the control, indicating that some microorganisms exhibited dominance following antibiotic treatment. At the phylum level, Bacteroidota was obviously dominant in the first 3 months in the antibiotic group relative to the control (Supplementary Figure S3). Proteobacteria and Desulfobacterota were dominant in the 1st and 2nd months. At the genus level, *Muribaculaceae* was obviously dominant in the first 3 months in the antibiotic group. It has been demonstrated that Proteobacteria is a microbial signature of dysbiosis in gut microbiota and that during the process of gut microbial dysbiosis, the adaptation of Proteobacteria will enhance, which makes them dominant [40]. Species difference analysis using the ALDEx2 tool showed that at the genus level, the 1st, 2nd, 3rd, and 7th months contained differential species between two groups (Supplementary Figure S4). *Enterococcus* was enriched in the antibiotic group in the 1st and 2nd months. *Enterococcus*, which belongs to opportunistic pathogens, can cause infections, such as urinary tract infections, bacteremia, and endocarditis [41]. Since antibiotic-resistant bacteria might multiply, antibiotic-induced reduction in microbial diversity did not necessarily mean a reduction in bacteria load [42]. On the whole, the Dominance index in the antibiotic group decreased with time (Fig. 2b, d), also manifesting a recovery trend to the level of control.

The Bray–Curtis distance was adopted to analyze β-diversity. The data on ASV abundances were used for the PCoA analysis. The results showed that samples from the 1st–5th, 7th, 9th, 10th, and 12th months were significantly separated (*P* < 0.05), while the 6th, 8th, 13th, and 14th months had more overlapped samples (Fig. 2e). Three non-parametric dissimilarity analyses (ANOSIM, Adonis, and MRPP) demonstrated that significant differences in microbial diversities were observed between antibiotic group and control, in particular in the first few months (Table S1).

It has been demonstrated that even a brief course of antibiotics can dramatically reduce gut microbial diversity [43], manifesting long-term negative impacts [44]. Perturbation (e.g., by antibiotics) to gut microbiota can shift the microbiome from its original equilibrium to another state [45, 46]. In this study, it was uncertain whether the mice’s gut microbiome in the antibiotic group had reached a new balanced state since the diversity in the last few months still exhibited fluctuations. It could be seen from Fig. 2a that the Chao1 index of the antibiotic group in the 14th month was lower than that of the control (*P* < 0.05). Studies showed that reduced diversity in gut microbiota may favor the colonization and overgrowth of pathogenic microorganisms [45]. The observed dominance of Proteobacteria and *Enterococcus* in our research was consistent with these studies.

Collectively, α- and β-diversity analyses suggested the following points: (1) ceftriaxone treatment in early life significantly reduced the microbial diversity in the gut of C57BL/6 mice in the first few months; (2) the gut microbiome manifested a recovery trend post the cessation of treatment, but the trend seemed somewhat unstable, implying that the negative impacts of ceftriaxone exposure were long-lasting.

### Effects of antibiotic exposure on gut molecular ecological networks (MENs)

**Fig. 3:**
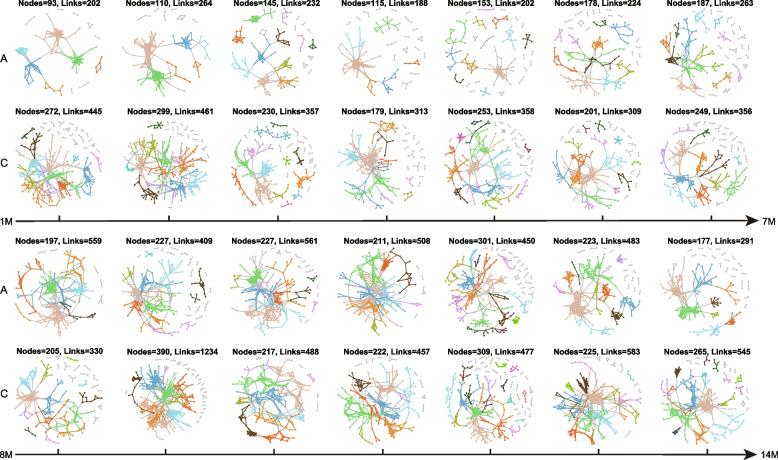
Visualization of MENs. MENs were constructed based on Pearson correlations of log-transformed ASV abundances. A and C represent the antibiotic group and control, respectively. M is short for month. Large modules (≥ 5 nodes) are indicated by different colors, and small modules (< 5 nodes) are shown in grey. Details of network topological parameters are shown in Table S2

In ecosystems, different species are interconnected, involving complicated ecological relationships, such as commensalism, mutualism, neutralism, amensalism, competition, predation, and parasitism. These association networks in microbial ecology are typically recognized as MENs, with species as nodes and their relationships as links [47]. We constructed 28 time-series MENs (empirical networks; Fig. 3 and Table S2) based on Pearson correlations of log-transformed amplicon sequence variant (ASV) abundances, followed by a random matrix theory-based method [28], providing a threshold for network construction. The empirical MENs exhibited obvious differences from random MENs (Table S3) and had scale-free characteristics (*R*^2^ = 0.515–0.946). The empirical MENs manifested small-world features with short geodesic distances (the average shortest path between two nodes) of 3.813–7.771 (Table S2), which allowed the effects of a perturbation to distribute quickly through the whole network, rendering the entire system efficient [26]. It could be seen from Fig. 3 that in the first few months, the MENs of the ceftriaxone group were obviously simpler than those of control, indicating that antibiotic exposure reduced the network complexity of gut microbiota. Nevertheless, the network complexity showed a recovery trend over time with fluctuations.

**Fig. 4:**
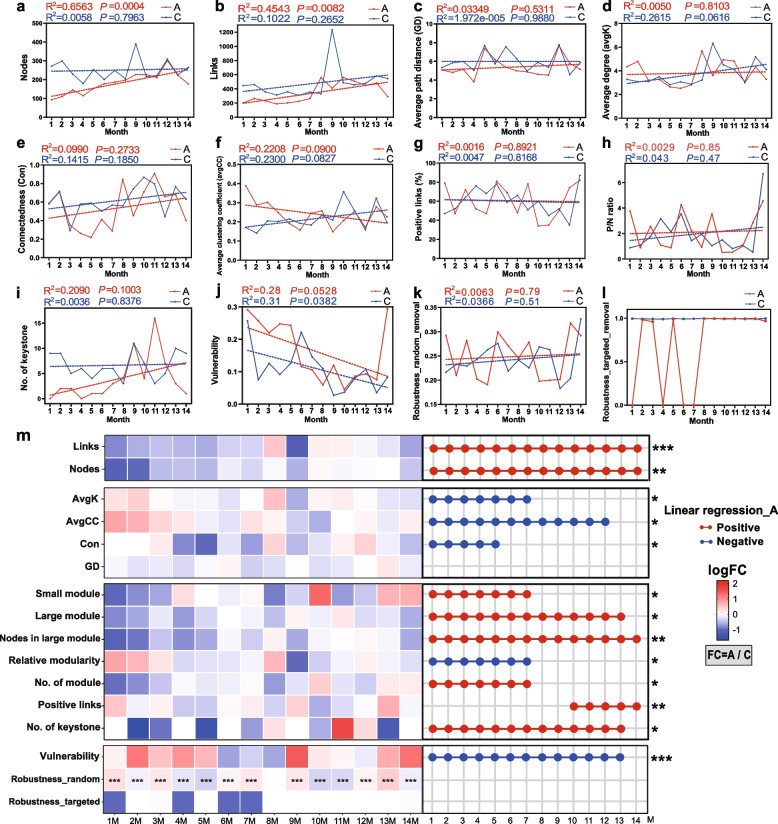
Visualization of network parameters. A and C represent antibiotic group and control, respectively. M is short for month. Detailed parameters of network indices are shown in Table S4. Network parameters were regressed against time. *R*^2^ and *P* values from regression analysis are shown. Detailed information on regression analysis is shown in Table S5. In **a**–**k**, red and blue dotted lines indicate the regression of the antibiotic group and control, respectively.** h** P/N means positive/negative. **k** Robustness determined as the proportion of taxa remained with 50% of nodes were randomly removed from each of the MENs. **l** Robustness was determined as the proportion of taxa remained with all module hubs removed from each of the MENs. **m** Heatmaps showing the relative magnitude of network indices between antibiotic group (A) and control (C). The values are represented by logFC (FC = A/C). The right half part of **m** shows the results of regression analysis (for the antibiotic group) with various indices against time. Connections between red nodes indicate that indices increase over time (positive), while connections between blue nodes represent that indices decrease with time (negative). For statistical analysis, normality testing was performed using the Shapiro–Wilk test. The data for robustness analysis were in accordance with normal distribution. Thus,* P* values of robustness_random were calculated using *t*-test. *P < 0.05; **P < 0.01; ***P < 0.001

To further examine whether and how antibiotic exposure affected the network complexity of gut microbiota, various network topological parameters were regressed against time. On the whole, network size (total number of nodes; *P* < 0.001, *R*^2^ = 0.6563; Fig. 4a) and network connectivity (total number of links; *P* < 0.01, *R*^2^ = 0.4543; Fig. 4b) increased across time in antibiotic group (Fig. 4m), while the variation trend in control group was not obvious. Nodes in MENs represent species. Figure 4a showed that in the first few months, the number of nodes in the ceftriaxone group was obviously less than that of control, thereafter it increased over time, approaching that of control. But in the 14th month obvious difference between the two groups still appeared. Therefore, the changing trend of nodes in the antibiotic group was consistent with that of Chao1 indices. The difference in average path distance (GD) between the two groups was not obvious (Fig. 4c). The average connectivity (average links per node; average K) in the 1st and 2nd months of the ceftriaxone group was larger than that of the control, followed by an obvious downtrend until the 7th month (Fig. 4d). Thereafter, variations of average K in the two groups exhibited similar trend with fluctuations. The connectedness (Con) in several months of ceftriaxone group was obviously lower than that of control, though the overall trend of Con in regression analysis was similar between the two groups (Fig. 4e). Interestingly, the average clustering coefficient (the extent to which nodes are clustered, average CC) in the first 4 months of the ceftriaxone group was higher than that of the control (Fig. 4f). Nevertheless, it manifested an overall downtrend, which was contrary to the average CC in control group.

Positive correlations between nodes generally indicate cooperative connections, such as commensalism, mutualism, syntrophic interactions, and shared environmental requirements [26]. Negative associations between nodes represent competition for limiting resources, amensalism, predation, parasitism, etc. It has been demonstrated that the positive/negative (P/N) ratio (the ratio of the number of positive links to the number of negative links) can reflect the balance between promoting and inhibiting interactions among microbial species in gut microbiota and that the P/N ratio may be one of the most critical changes in a disordered microbiome [48]. The P/N ratio was much larger in diseased networks, whereas more negative links appeared in healthy microbial networks [48]. It has been shown that cooperation reduces the stability of the microbiome, whereas competition increases the stability [49, 50]. Networks with mutually beneficial and competitive associations are more robust and stable [31]. In this study, although the overall variation trend of positive links was similar between the two groups as shown by regression analysis (Fig. 4g), obvious differences were observed in the 1st, 4th, 5th, 8th, 9th, 10th, and 13th months. The positive associations and P/N ratio (Fig. 4h) of the ceftriaxone group in the 1st month were obviously higher than those of the control, indicating that antibiotic exposure enhanced positive associations in the gut MENs, which might result in less robust and less stable networks in the gut microbiome. Thereafter, the P/N ratio exhibited a recovery trend, yet obvious differences were observed in the 4th, 5th, 9th, 13th, and 14th months.

**Fig. 5:**
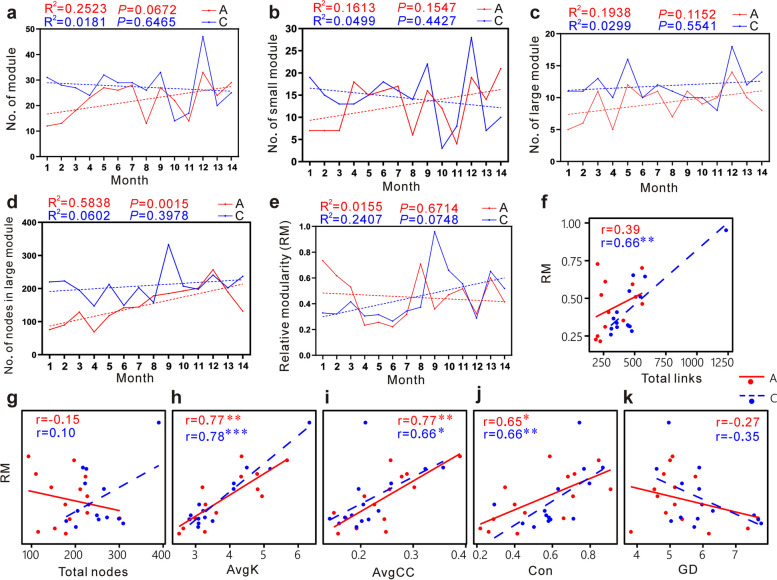
Module indices and correlations between relative modularity (RM) and other network parameters. A and C represent the antibiotic group and control, respectively. **a**–**e** Module parameters were regressed against time. *R*^2^ and *P* values from regression analysis are shown. Red and blue dotted lines indicate the regression of the antibiotic group and control, respectively. **f**–**k** Red solid line and blue dashed line represent the antibiotic group and control, respectively. The Spearman correlation coefficients (*r*) are shown for the antibiotic group and control in corresponding colors. *P < 0.05; **P < 0.01; ***P < 0.001

Changes in network structure can further lead to variations in network organizational principles, such as modularity. Modularity reflects the extent to which a network is compartmentalized into different modules, in which the nodes within a module closely connect with each other but are less associated with nodes from other modules [26]. A network is considered to have good modularity when the modularity value is greater than 0.4. Module can be categorized into small modules (< 5 nodes) and large modules (≥ 5 nodes) [26]. All the networks we constructed had good modularity (modularity was between 0.594 and 0.848; Table S2). The number of modules in the first 3 months of the antibiotic group was obviously less than that of the control (Fig. 5a and Supplementary Figure S5) but with an overall increasing trend over time (*R*^2^ = 0.2523, *P* = 0.0672). The number of modules in the antibiotic group gradually recovered to be consistent with the control across time, though in the 8th, 10th, and 12th months, the differences were obviously observed. It suggested that there were some fluctuations during the recovery process. The number of small modules in the first 3 months of the antibiotic group was obviously less than that of control. It had an overall increasing trend, recovering to approach the baseline of the control over time, but the obvious difference still appeared in the 14th month (Fig. 5b). The number of large modules in the first 5 months of the antibiotic group was obviously less than that of control (Fig. 5c). In the 6th, 7th, 9th, 10th and 11th months the number of large modules in the antibiotic group approached that of control. However, the recovery trend was unstable, since the number of large modules in the antibiotic group was still obviously less than that of control in the 8th, 12th, 13th, and 14th months. Similarly, the number of nodes in large modules of the antibiotic group exhibited a recovery trend post-cessation of treatment, whereas obvious differences between the two groups reappeared in the 14th month (Fig. 5d). The abundance proportions of bacterial species in each module and the bacteria correlations within and among modules were shown in Supplementary Figure S5. Besides affecting the number of modules, antibiotic exposure also led to an obvious alteration of bacterial composition and their associations in MEN modules, though the composition exhibited a slight recovery trend in the last few months.

Since network size and connectivity vary among different MENs, relative modularity (RM) is more meaningful for comparing module structure among different networks [26]. Interestingly, RM in the first 3 months of the antibiotic group was obviously higher than that of the control (Fig. 5e). Thereafter, RM between the two groups in the 4th, 5th, 6th, 7th, 11th, 12th, and 13th months was similar, respectively, while obvious differences were observed in the 8th, 9th, 10th, and 14th months, respectively. The Spearman correlation analysis showed that both the RM in the ceftriaxone group and control increased with links, average K, average CC, and Con, respectively (Fig. 5f, h, i, and j). Interestingly, RM in the control group was positively correlated with nodes, but in the ceftriaxone group, it exhibited a negative correlation (Fig. 5g). Both the RM in the ceftriaxone group and control decreased with GD (Fig. 5k). These results suggested that the relationships between RM and some parameters of network structure were overall similar between the ceftriaxone group and control, though some differences existed.

The changed network complexity may lead to changes in the role of specific species within the network. Species that play key roles in shaping network structure are regarded as keystone nodes [30, 51]. Regression analysis showed that the number of keystones in the antibiotic group manifested an overall increasing trend (Fig. 4i). The trend seemed to recover to the baseline of control over time, though fluctuations in some months occurred. Specifically, the numbers of keystones of the antibiotic group in the 1st–7th months were obviously less than those of control, indicating that antibiotic use obviously reduced the number of key species in gut MENs. The numbers of keystones in the 8th, 9th, 10th, and 12th months were similar between the two groups. But obvious fluctuations appeared in the 11th, 13th, and 14th months. In addition, the keystones-affiliated taxa in the 1st–13th months were entirely different between the two groups, and only one keystone was shared in the 14th month (Table S6). These results suggested that keystones were obviously different between the ceftriaxone group and the control and that new keystones appeared in the antibiotic group during the recovery process of network complexity. The observed differences in keystones might be a critical promoting factor in shaping different microbial network structures between the two groups.

Network vulnerability (the maximum decrease in network efficiency when deleting a single node from the network) of the ceftriaxone group was higher than that of control in most months, in particular in the first few months, as well as the 9th and 14th months (Fig. 4j). Both the network vulnerability in these two groups manifested decreasing trend over time. Robustness (the resistance to node loss) on the basis of random species loss (robustness_random) exhibited obvious differences between the two groups in the 1st, 3rd, 4th, 5th, 7th, 9th, 10th, 11th, 13th, and 14th months (Fig. 4k, m). Robustness based on targeted removal of keystones (robustness_targeted) exhibited fluctuations of up and down from the 1st to 8th months in the ceftriaxone group (lower than that of control in some months), thereafter it maintained at levels similar to that of control (Fig. 4l). Collectively, these results suggested that antibiotic exposure reduced gut MEN stability in the first few months, followed by recovery trend though it didn’t entirely recover to the level of control.

In summary, most network parameters, including nodes, links, average K, average CC, number of modules, number of small modules, number of large modules, number of nodes in large modules, RM, number of keystones, positive links, P/N ratio, robustness, and vulnerability, were obviously different between two groups in the first few months. The results suggested that ceftriaxone exposure markedly changed the gut MENs, including network complexity and network stability. As time went on, the network parameters showed an overall recovery trend, whereas they didn’t entirely recover to the baseline of control, suggesting that the negative impacts of ceftriaxone use in early life on the gut MENs were long-lasting. It should be noted that the microbial diversity, network complexity, and network stability were not maintained to certain levels in the 14-month time series in the control group, with fluctuations over time. This phenomenon was consistent with reported viewpoints that the gut microbial diversity changed with age [42] and that characteristics of MENs are dynamic over time [26].

### Relationship between MEN complexity and stability

**Fig. 6:**
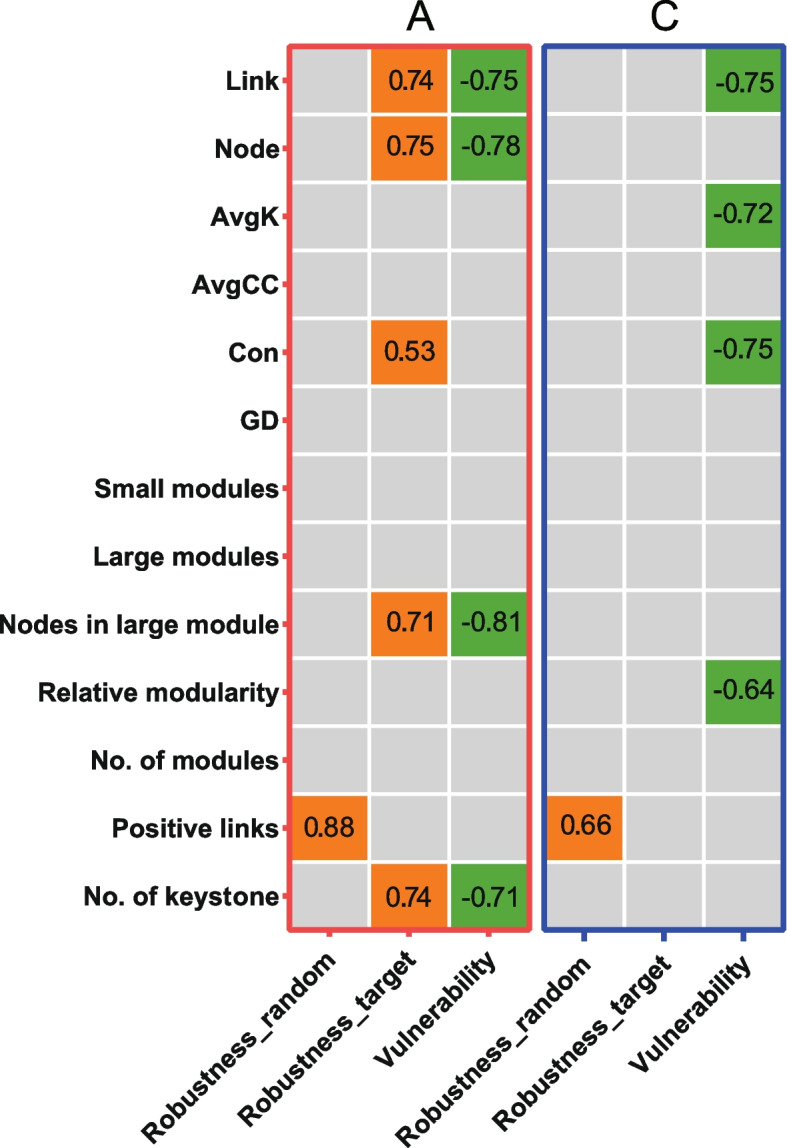
Correlations between network complexity and stability. Correlations with statistical significance (*P* < 0.05) are shown, with orange grids indicating positive correlations and green grids indicating negative correlations. Numbers inside the grids represent corresponding correlation coefficients. Correlations with no statistical significance are indicated by grey. Detailed information about the correlations is shown in Table S7

Whether and how MEN complexity affects ecosystem stability has been a controversial question for many years [31, 52–56], which remains understudied in the areas of microbial ecology [26]. Researchers found that network stability in grassland soil microbial communities under warming strongly correlated with network complexity, which was consistent with the central ecological belief that complexity leads to stability [26]. Nevertheless, this observed phenomenon might not be necessarily applicable to other ecosystems since controversial results regarding the relations between network stability and complexity have been reported [31, 55, 56]. To determine whether and how MEN complexity in gut microbiota under antibiotic challenge affects network stability, correlation analysis was performed between the parameters of network complexity and stability (Fig. 6 and Table S7). Significant correlations were observed in antibiotic group between various complexity parameters and network stability, while the correlations with statistical significance in the control group were relatively less. Both the network robustness based on random species loss (“robustness_random”) in the antibiotic group (*r* = 0.88) and the control (*r* = 0.66) were positively correlated with positive links. Robustness based on targeted removal of keystones (“robustness_target”) in the antibiotic group was positively correlated with links, nodes, Con, nodes in large modules, and the number of keystones, respectively. However, no correlation with statistical significance regarding “robustness_target” in the control group was found. Vulnerability in the antibiotic group was negatively correlated with links, nodes, nodes in large modules, and the number of keystones, respectively. Vulnerability in the control group was negatively correlated with links, average K, Con, and relative modularity, respectively. These results suggested the following points. On one hand, network complexity is related to network stability in the gut microbial community, whether it is under antibiotic exposure or not. On the other hand, since the correlations between MEN complexity and stability, as well as the measurements of complexity and stability, were significantly different between the two groups, it was plausible that antibiotic-induced changes in gut MEN complexity affected the network stability. These results also supported the ecological theory that complexity begets stability [57].

### Effects of antibiotic exposure on gut microbial metabolism

**Fig. 7:**
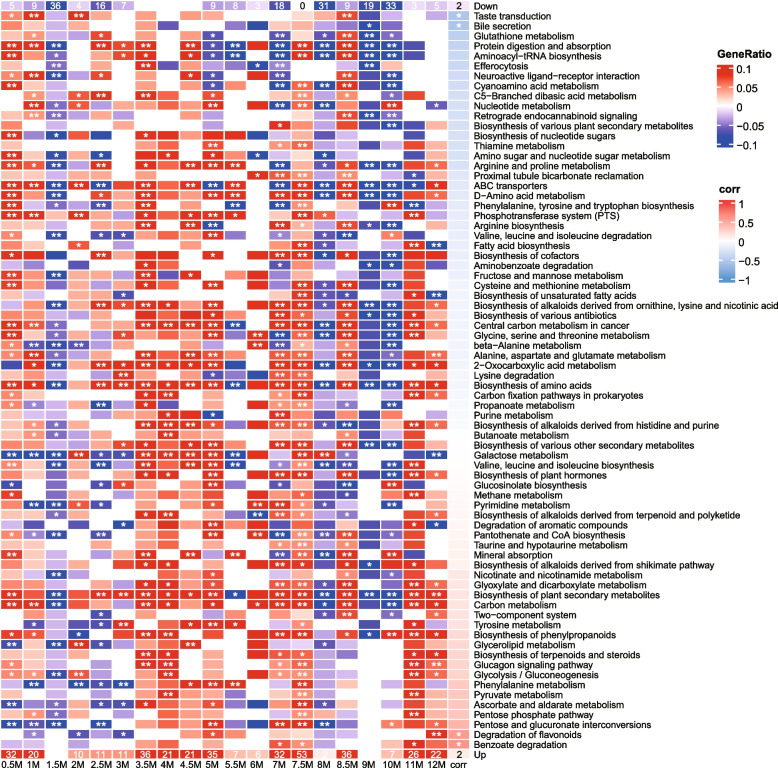
Metabolic pathways with significant differences in metabolome analysis. The horizontal direction on the bottom indicates the sampling time. M is short for month. Samples from a total of 20 time points were used for metabolomics analysis. The enriched pathways were produced by annotation of differential metabolites in the KEGG database (organism group: bacteria). Pathways with differential metabolites contained in over 60% of time points (i.e., > 12 time points) are shown. The heatmap colors were generated from the GeneRatio. The GeneRatio was the ratio of the number of metabolites enriched in a specific pathway to the total number of metabolites in this pathway, with + /– values to discriminate the upregulation in the antibiotic group (red) and control (blue), respectively. The first line marked by “Down” indicates the number of metabolic pathways (blue; with significance) enriched by metabolites with higher abundances in the control group at each time point. The last line marked by “Up” indicates the number of metabolic pathways (red; with significance) enriched by metabolites with higher abundances in the antibiotic group. The rightmost column of grids marked by “corr” indicates the correlation between GeneRatio values and sampling time, with red and blue representing positive and negative correlations, respectively. In some months, some differential metabolites are not contained in specific metabolic pathways, which are represented by white grids. In other grids, red and blue indicate that the differential metabolites were in higher abundance in the antibiotic group and control, respectively. *P < 0.05; **P < 0.01

Metabolomics analysis showed that numerous metabolites with significant differences between the antibiotic group and the control were identified, with variations of relative content over time (Supplementary Figure S6). In each month the number of metabolites detected, the number of up- and down-regulated metabolites in the antibiotic group, and the number of annotated pathways were shown in Table S8. The orthogonal to partial least squares discriminant analysis (OPLS-DA) also indicated that metabolites between the two groups were significantly different (Supplementary Figure S7). At most time points the differences between two groups are larger than that within a single group. Figure 7 showed the main metabolic pathways with significant differences between the two groups, of which pathways related to amino acid metabolism (15 pathways) were the most significantly affected by antibiotic exposure. The results also showed that some pathways displayed significant differences between the two groups at most time points, suggesting that the post-antibiotic effect on these pathways was long-lasting. For instance, “biosynthesis of amino acids” displayed significant differences between the two groups at 18 time points (18/20; a total of 20 time points were detected). There were six pathways related to carbohydrate metabolism with significant differences between the two groups. Many other metabolic pathways, such as pathways related to lipid metabolism and nucleotide metabolism, also exhibited significant differences. Carbohydrate metabolism, amino acid metabolism, and lipid metabolism are three main material metabolisms. Nucleotide metabolism that involves genetic information transmission, such as DNA synthesis and RNA synthesis, is also vitally important to organisms. These results suggested the following points: (1) ceftriaxone exposure significantly impacted the metabolisms of the murine intestinal microbiota, which might lead to the occurrence of metabolic diseases if the observed results were applicable to humans; (2) the post-antibiotic effect on some gut microbial metabolic pathways was long-lasting.

### Relationship between gut MENs and metabolome following antibiotic exposure

To determine the relation between MENs and metabolome, correlation analysis was carried out between various network parameters and the metabolic pathways with significant differences between the ceftriaxone group and control. There were 48 and 49 correlations with statistical significance in the ceftriaxone group and control, respectively (Supplementary Figure S8). In the ceftriaxone group, 21 pathways contained statistically significant correlations with network parameters. Among these metabolic pathways, there were 7 pathways with significant correlations related to amino acid metabolism, including “valine, leucine, and isoleucine biosynthesis”, “phenylalanine, tyrosine, and tryptophan biosynthesis”, “valine, leucine, and isoleucine degradation”, “phenylalanine metabolism”, “tyrosine metabolism”, “taurine and hypotaurine metabolism”, and “glycine, serine, and threonine metabolism”. “Valine, leucine, and isoleucine biosynthesis” was positively correlated with total nodes and robustness_target, respectively, and was negatively correlated with average CC, robustness_random, and vulnerability, respectively. There was one pathway (galactose metabolism) with statistical significance related to carbohydrate metabolism and one pathway (biosynthesis of unsaturated fatty acids) related to lipid metabolism. In the control group (Supplementary Figure S8b), 33 pathways contained statistically significant correlations with network parameters. Among these metabolic pathways, there were 7 pathways with significant correlations related to amino acid metabolism, including “valine, leucine, and isoleucine biosynthesis”, “biosynthesis of amino acids”, “phenylalanine, tyrosine, and tryptophan biosynthesis”, “valine, leucine, and isoleucine degradation”, “glycine, serine, and threonine metabolism”, “lysine degradation”, and “cysteine and methionine metabolism”. “Valine, leucine, and isoleucine biosynthesis” was positively correlated with average CC and robustness_random, respectively, and was negatively correlated with the number of keystones. There were 7 pathways related to carbohydrate metabolism and one pathway (glycerolipid metabolism) related to lipid metabolism. Obviously, the correlations between network parameters and metabolic pathways in the two groups were different. On one hand, there was a difference in the number and type of metabolic pathways with statistical significance in the correlation analysis. On the other hand, for the same metabolic pathway, network parameters from the two groups correlated with the pathway with statistical significances were almost different. Collectively, these results suggested that the antibiotic exposure-induced changes of metabolic pathways (in particular the ones related to amino acid metabolism) in gut microbiota were related to the variations of MENs, though their causal relation remained to be investigated.

### Post-antibiotic effect on gut microbial metabolic function

To further study the post-antibiotic effect, gut microbial metabolic function was predicted based on the 16S rRNA gene-sequencing data. Besides, metagenomics analysis was performed. In terms of the number of pathways with significant differences (corrected *P* value < 0.05) between the two groups, the 1st month (118 pathways) was the largest (Table S9). The number displayed a decreasing trend in the following several months, then with fluctuation, and finally it was still greater than zero. It suggested that in the 14th month, there were still differences in microbial metabolic pathways between the two groups. More specifically, the number of dominant pathways in the control group in the 1st month was 80, which was much greater than that of the antibiotic group, suggesting that many pathways had been impaired by antibiotic exposure. The number in the control group displayed a decreasing trend in the following several months, then with fluctuation, and finally it was still greater than that of the antibiotic group in the last 3 months (Table S9). Most pathways with significant differences were crucial to bacterial survival and physiological functions. Obviously, antibiotic exposure has made the gut microbiota more vulnerable. The variation trend of the number of metabolic pathways with significant differences was similar to that of microbial network parameters. For instance, the values of network vulnerability in the antibiotic group in the first few months were greater than those of control (Fig. 4j), suggesting that antibiotic exposure made the gut microbial network more vulnerable or less stable. Afterward, the vulnerability displayed a recovery trend with fluctuation in the level of control. But it did not entirely recover. These results further indicated that the changes in gut microbial metabolism following antibiotic exposure were related to MEN variations.

As shown in Supplementary Figure S9, in the 1st month eight dominant pathways related to antibiotic biosynthesis were found in the control group, whereas only two pathways were dominant in the antibiotic group. Competition increases microbial network stability, whereas cooperation reduces the stability [49, 50]. Some bacteria can secrete antibiotics to inhibit other microorganisms, which is a phenomenon of competition. The less dominant pathways related to antibiotic biosynthesis in the antibiotic group might beget reduced competition, which might reduce the microbial network stability. Indeed, the gut microbial network stability was markedly weakened post-antibiotic exposure (Fig. 4). Afterward, the network stability exhibited a recovery trend with fluctuations, but it did not entirely recover to the level of control. The change trend of dominant pathways related to antibiotic biosynthesis (Table S9) was similar to that of the network stability.

**Fig. 8:**
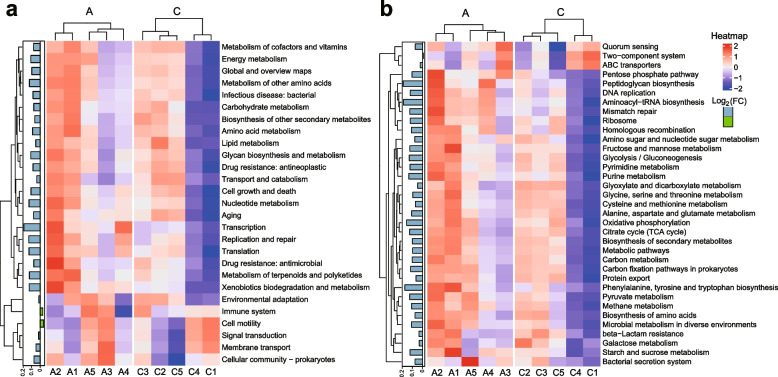
Abundance analysis of metabolic pathways between the antibiotic group and control based on metagenomic sequencing data. The data were from metagenomic analysis of feces samples in the 12th month after the cessation of antibiotic exposure. The data were filtered to exclude the reads from the mice. The heatmap was produced using the ComplexHeatmap in R package (version 4.2.1). The horizontal columns on the left side of each figure display the relative abundance between the two groups. A and C represent the antibiotic group and control, respectively. FC represents the ratio of abundance (A/C). **a** The KEGG pathways were from the level_2 category. **b** The KEGG pathways were from the level_3 category. Pathways ranking in the top 35 in average abundance were shown in each figure, excluding the pathways not associated with microbes

“Xenobiotics biodegradation and metabolism” was dominant in the antibiotic group in the 1st, 9th, 11th, and 12th months post-antibiotic exposure (Supplementary Figure S10). Metagenomic sequencing data also showed that this pathway was dominant in the antibiotic group in the 12th month after the cessation of antibiotic exposure (Fig. 8a). More specifically, there were 6, 6, and 8 dominant pathways related to the degradation of organic toxicants in the antibiotic group in the 1st, 9th, and 11th months, respectively (Supplementary Figure S9 and Table S9), while the number of dominant pathways in the control was 1, 1, and 2, respectively. The relative dominance trend (Table S9) was similar to the changing trend of microbial network parameters (Fig. 4). Generally, antibiotic treatment was a stress for gut bacteria. The stress might have caused stress reactions in a portion of bacteria, which made them resistant to external harmful factors. Thus, we observed the phenomenon that in the 1st month post-antibiotic exposure more dominant pathways related to the degradation of organic toxicants were in the antibiotic group (relative to control). The recurrence of the phenomenon in the 9th and 11th months might be caused by the long-term post-antibiotic effect, as we observed a similar effect on the microbial network. Additionally, “quorum sensing” (QS) was dominant in the 1st and 12th months in the antibiotic group (Supplementary Figure S9). There was no difference between the two groups in other months. Increasing evidence shows that QS can enhance the stress response in bacteria [58]. QS is an important dependent pathway for bacterial communication [59]. The inter-species and inter-kingdom communication mediated by QS could shape the gut microbial community. Besides, it has been demonstrated that QS plays an important role in alleviating dysbiosis of the gut microbiota caused by antibiotic exposure in mice [60]. It was therefore not surprising that the enrichment of QS in the antibiotic group in the 1st month post-antibiotic treatment was observed in the present study. The enriched QS could play a role in the stress response to antibiotic exposure. The microbial network parameters (Figs. 4 and 5) showed that there was still slight fluctuation in the 12th month, displaying the long-term post-antibiotic effect. Since the microbial network involved inter-species and inter-kingdom communication, it was plausible that the recurrence of QS enrichment in the antibiotic group in the 12th month might be induced by the long-term post-antibiotic effect. Indeed, metagenomic sequencing data also showed that QS was relatively dominant in the antibiotic group in the 12th month (Fig. 8b).

As shown in Supplementary Figure S9, “beta-lactam resistance” was enriched in the antibiotic group in the 9th month, and “vancomycin resistance” was dominant in the 9th and 11th months. Ceftriaxone belongs to beta-lactam antibiotics. Metagenomic sequencing data showed that “beta-lactam resistance” was relatively dominant in the antibiotic group in the 12th month (Fig. 8b). These results suggest that there is still a risk of developing antimicrobial drug resistance even after a brief ceftriaxone exposure in early life. Interestingly, pathways belonging to “drug resistance: antineoplastic” in the antibiotic group were weakened in the 1st, 8th, 9th, and 11th months, respectively (Table S9). These pathways included “platinum drug resistance” and “antifolate resistance”. It had been recently demonstrated that some gut microorganisms could overcome resistance to antitumor drugs [61]. It was plausible that ceftriaxone exposure might result in the relative enrichment of some microorganisms, and accordingly their roles in overcoming antitumor drug resistance displayed.

### Changes in functional networks caused by antibiotic effect

A total of 28 time-series functional networks (Supplementary Figure S11 and Table S10) were constructed based on 16S rRNA gene-sequencing data. As shown in Table S11, the number of network clusters in the antibiotic group was greater than that of control in the 1st, 2nd, 6th, 7th, 8th, 9th, 10th, 11th, and 13th months, respectively. We compared the pathway composition of clusters (ranking in the top 3 with a score) between the two groups in each month and found that in most comparisons the number of shared pathways between two clusters from the two groups was small (Table S12), indicating the difference in pathway composition of clusters between the two groups. It could be seen from Table S11 and Figure S11 that most clusters from the two groups contained different numbers of nodes or links, indicating the difference in the topology structure of functional networks. Additionally, we constructed functional networks (Supplementary Figure S12 and Table S13) based on metagenomic sequencing data from the 12th-month samples. The number of network clusters in the antibiotic group was smaller than that of the control (Table S14), which was consistent with the functional network result (the 12th month; Table S11) based on the 16S rRNA gene-sequencing data. Some clusters from the two groups also contained different numbers of nodes or edges (links). Analysis of shared pathways between arbitrary two clusters (ranking in the top 3) from the two groups showed that there were differences in pathway composition among the clusters (Table S15). Thus, the topological structure of functional networks from the antibiotic group and control group was obviously different (Supplementary Figure S13). Collectively, these results suggested that ceftriaxone treatment caused changes in functional networks, including the variations of network topology, the number of network clusters, and the pathway composition of clusters. The ceftriaxone effect on gut microbial functional networks in C57BL/6 mice was long-lasting.

### Epilogue

Microorganisms in gut microbiota are interconnected, with complicated associations that can be represented by MENs [47]. Gut microbial dysbiosis may lead to the occurrence of various diseases, such as metabolic diseases [42]. Antibiotic exposure is not uncommon, which can be found in medical settings and from environmental sources. Thus, great concern should be raised about antibiotic exposure. Since this study showed that the antibiotic exposure-induced changes of gut MENs were long-lasting and that the MEN variations were associated with microbial metabolism changes, we propose that restoration of the gut MENs is critically important for the treatment of disorders and diseases caused by antibiotic exposure, such as metabolic diseases.

Ceftriaxone, a β-lactam antibiotic with comparable activities against medically important pathogens, belongs to third-generation cephalosporins and is commonly applied to clinical practice [62]. Ceftriaxone-induced intestinal dysbacteriosis is a focus of research and a growing health concern [63]. In terms of application, ceftriaxone can be administrated via intraperitoneal injection [64], gavage [21], subcutaneous injection [65], intravenous injection [66], aerosol inhalation delivery [67], and intramuscular injection [68], among which intravenous injection and intramuscular injection are two typical administration methods in clinical treatment. In this study, we adopted gavage administration referring to the practice of many researchers who administered ceftriaxone orally to mouse models [21–23]. Ceftriaxone, soluble in water, is not easily absorbed by the intestine. Gavage administration of ceftriaxone to mice contributes to observing the drug’s direct impacts on the gut microbiota. A prospective cohort study, using different methods of antibiotic administration, and using different antibiotics can be conducted for further studying the effects of antibiotics on the molecular ecological networks and metabolism in gut microbiota. Results from these researches might provide more new discoveries and provide more guidance for antibiotic use.

## Conclusion

In summary, this study provides new and deep insights into the long-term effects of brief antibiotic exposure in early life on mice’s gut microbial diversity, MENs, and metabolism. Antibiotic use in early life caused significant changes in microbial diversity, metabolism as well as MEN complexity and stability. Changes in network complexity have affected network stability. Antibiotic-induced differences in gut microbial metabolism were related to MEN variations. Post cessation of antibiotic treatment, the microbial diversity and MENs exhibited a recovery trend, but they didn’t entirely recover to the levels of control, suggesting that the negative impacts of antibiotics on gut microbial diversity and MENs were long-lasting. Antibiotic treatment also caused long-term effects on gut microbial functional networks in mice. Therefore, great concern should be raised about children’s brief exposure to antibiotics if the results observed in mice are applicable to humans.

### Supplementary Information

**Additional file 1.** Research ethics approval.

**Additional file 2: Supplementary Figure S1.** Weight changes of mice over time. Triangles and solid dots indicate the mean weight of mice. A and C represent antibiotic group and control, respectively. The middle of boxplot represents median; the top and the bottom of a box represent upper quartile and lower quartile, respectively; bars at the top and the bottom show the maximum and minimum, respectively, after excluding the abnormal values. Antibiotic group and control are shown in red and blue, respectively. Hollow circles indicate the abnormal values. Day 0 means the endpoint of 8-day antibiotic treatment. For statistical analysis, normality test and homogeneity test of variance were performed. If it met the parameter test conditions, *t*-test was performed; otherwise, the Wilcoxon rank sum test was carried out. *P < 0.05 indicates statistic difference. **Figure S2.** Rarefaction curves in microbial diversity analysis. A and C represent antibiotic group and control, respectively. M is short for month. **Figure S3.** Alluvial diagrams of species composition across time. The data are from 16S rRNA gene sequencing. A and C represent antibiotic group and control, respectively. The figures were produced using the R package (version 4.1.2). Each column indicates species composition proportion. In b the top 20 genera with relative abundance are shown. Other genera not ranking in the top 20 are combined and named as “other”. **Figure S4.** Species difference analysis at the genus level using the ALDEx2 tool. The data are from 16S rRNA gene sequencing. A and C represent antibiotic group and control, respectively. M is short for month. The screening criteria of differential species are as follows: absolute value of “Effect” > 1; FDR < 0.05. Only the 1st (a), 2nd (b), 3rd (c), and 7th (d) months contain differential species at the genus level. **Figure S5.** Visualization of network modules. The data analysis is based on 16S rRNA gene sequencing. A and C represent antibiotic group and control, respectively. M is short for month. The number of modules, nodes, links, and positive links are shown. The left side of each panel shows the schematic diagram of each module, while the right side (pie chart) shows the microbial composition (at the phylum level) of each corresponding module. Pie charts show the modules with > 10 nodes. Nodes with different colors in the modules represent different microbes at the phylum level. Module hubs and connectors are shown in the modules. Green line and yellow line indicate positive and negative associations, respectively. **Figure S6.** Differential metabolites identified by metabolomics analysis. Horizontal direction indicates sampling time. M is short for month. FC is short for fold change (A/C, where A and C represent antibiotic group and control, respectively). Grids from the last column indicate the correlation between FC values of differential metabolites and sampling time, with red and blue representing positive and negative correlations, respectively. In other grids, red and blue indicate the differential metabolite with higher abundance in antibiotic group and control, respectively. The Arabic numbers in the first (marked by “Up”) and last (marked by “Down”) lines represent the number of up-regulated and down-regulated metabolites at each time point, respectively. *P < 0.05; **P < 0.01. **Figure S7.** Differential analysis of metabolites between ceftriaxone group and control. The analysis was performed using the orthogonal to partial least squares discriminant analysis (OPLS-DA). a Samples from antibiotic group and control are represented by red and blue, respectively. Horizontal direction indicates the differences between groups, and longitudinal direction represents the differences within a single group. b r < 0 indicates that the difference between groups is less than the difference within a single group; r > 0 indicates that the difference between groups is larger than the difference within a single group.

**Additional file 3: Supplementary Figure S8.** Correlations between network parameters and metabolic pathways with significant differences. The network parameters are from the data of microbial MENs. Correlations for antibiotic group and control are shown in a and b, respectively. The enriched pathways were produced by annotation of differential metabolites in the KEGG database (organism group: bacteria). The correlations were carried out between –lg(*P* value of pathway enrichment) and network parameters, generating correlation coefficients and corresponding *P* values. Red and blue represent positive and negative correlations, respectively. avgK, average K; avgCC, average clustering coefficient; Con, connectedness; GD, average path distance. *P < 0.05; **P < 0.01.

**Additional file 4: Supplementary Figure S9.** Metabolic function prediction on KEGG level_3 category based on the data of 16S rRNA gene sequencing. The metabolic function potential of microbial communities was predicted using the Tax4fun2 in R package and the STAMP software. A and C represent antibiotic group and control, respectively. 1M means the 1st month, and so on. Pathways with corrected *P* values less than or equal to 0.05 are shown.

**Additional file 5: Supplementary Figure S10.** Metabolic function prediction on KEGG level_2 category based on the data of 16S rRNA gene sequencing. A and C represent antibiotic group and control, respectively. 1M means the 1st month, and so on. Pathways with corrected *P* values less than or equal to 0.05 are shown. There is not any pathway with significant difference in the 5th or 10th month.

**Additional file 6: Supplementary Figure S11.** Functional networks based on the 16S rRNA gene-sequencing data. A and C represent antibiotic group and control, respectively. Pathways and the correlation between pathways are represented by nodes and links, respectively. In each subfigure different subnetworks are represented by different colors. Yet, it does not mean that in groups A and C the same colour indicate the same subnetwork. The unclustered pathways are displayed in the grid layout.

**Additional file 7: Supplementary Figure S12.** Functional networks based on metagenomic sequencing data from the 12th month samples. A and C represent antibiotic group and control, respectively. Pathways and the correlation between pathways are represented by nodes and edges, respectively. The metabolic pathways were annotated at the level_3 categories in the KEGG database. In each subfigure different subnetworks are represented by different colors. Yet, it does not mean that in groups A and C the same colour indicate the same subnetwork. The unclustered pathways are displayed in the grid layout.

**Additional file 8: Supplementary Figure S13.** Functional networks constructed using KO number (protein/enzyme) and the correlation between KO as nodes and edges (links), respectively. The networks were constructed based on metagenomic sequencing data. Core subnetworks were extracted mainly based on the following parameters: degree cutoff 2; K-core 5; Max. depth 100. A and C represent antibiotic group and control, respectively. In each subfigure different subnetworks are represented by different colors.

**Additional file 9: Table S1.** Dissimilarity comparison of microbial diversities between antibiotic group and control.

**Additional file 10: Table S2.** Parameters of empirical networks.

**Additional file 11: Table S3.** Comparisons between empirical MENs and random MENs.

**Additional file 12: Table S4.** Detailed information of network indices for plotting of heatmap.

**Additional file 13: Table S5.** Regression analysis of network indices against time.

**Additional file 14: Table S6.** Keystones in antibiotic group and control in each month.

**Additional file 15: Table S7.** Detailed information of correlations between network complexity and stability.

**Additional file 16: Table S8.** The number of identified metabolites and pathways in metabolomics analysis.

**Additional file 17: Table S9.** The discussed pathways with significant difference between two groups.

**Additional file 18: Table S10.** Detailed information of functional networks based on the 16S rRNA gene-sequencing data.

**Additional file 19: Table S11.** Statistics of functional networks based on 16S rRNA gene-sequencing data.

**Additional file 20: Table S12.** Shared pathways between two clusters from antibiotic group and control.

**Additional file 21: Table S13.** Detailed information of functional networks based on the metagenomic sequencing data.

**Additional file 22: Table S14.** Statistics of functional networks based on metagenomic sequencing data.

**Additional file 23: Table S15.** Shared pathways between two clusters from antibiotic group and control based on metagenomic sequencing data.

## Data Availability

Raw sequences of 16S rRNA gene sequencing were deposited in the Sequence Read Archive (SRA) under accession number PRJNA863425. The metagenomic sequencing data had been deposited in the China National Center for Bioinformation under accession number PRJCA019236.
