# Supplementary material for: Short-term exposure to antibiotics begets long-term disturbance in gut microbial metabolism and molecular ecological networks
Source: Microbiome. 2024 May 7;12:80. doi: 10.1186/s40168-024-01795-z (PMC11075301; doi:10.1186/s40168-024-01795-z)
Supplement: Supplementary file 2 — Additional file 1. Research ethics approval. [file 40168_2024_1795_MOESM1_ESM.pdf]

The Suggestion Of Animal Research Ethics

ID Number GDY2004001

<table>
<tr><td>Project Title</td><td colspan="4">Gut microbial metabolism and molecular ecological networks</td></tr>
<tr><td>Experimenter：</td><td colspan="4">Guo Lianxian</td></tr>
<tr><td>Project Organization：</td><td colspan="4">School of Public health, Guangdong Medical University</td></tr>
<tr><td rowspan="4">Situation of using laboratory animal</td><td colspan="4">Source of laboratory animal: Guangdong Medical Laboratory Animal Center</td></tr>
<tr><td>Species or strain</td><td>C57BL/6 mice</td><td colspan="2">Grade SPF</td></tr>
<tr><td colspan="2">Age 28-30 day</td><td colspan="2">Weight 13-15g</td></tr>
<tr><td colspan="2">Quantity 100 pcs
♀ 0 pcs ♂ 100 pcs</td><td colspan="2">Experimental date：2020.9.30 -2021.12.23</td></tr>
<tr><td colspan="5">Based on principles of animal research ethics, the experiment is approved for meeting the generally accepted 3R (Reduction, replacement, Refinement) principles such as the selection of laboratory animal, experimental purpose, methods, observation indicator, execution methods of experimental animals after experiments etc.</td></tr>
<tr><td>Condition of the barrier housing facility</td><td colspan="4">This housing facility is a barrier housing facility, and it's in keeping with national standard. The care of laboratory animal and the animal experimental operation have conforming to 《Administration Rule of Laboratory Animal》, et al.</td></tr>
<tr><td colspan="5">Comments of laboratory animal ethical committee(LAEC)

After acessment, this project meets the relevant ethical requirements.

Stamp: laboratory animal ethical committee, Guangdong Medical University

Date(yy/mm/dd)：2020-8-25</td></tr>
</table>
