# Supplementary material for: Short-term exposure to antibiotics begets long-term disturbance in gut microbial metabolism and molecular ecological networks
Source: Microbiome. 2024 May 7;12:80. doi: 10.1186/s40168-024-01795-z (PMC11075301; doi:10.1186/s40168-024-01795-z)
Supplement: Supplementary file 3 — Additional file 2: Supplementary Figure S1. Weight changes of mice over time. Triangles and solid dots indicate the mean weight of mice. A and C represent antibiotic group and control, respectively. The middle of boxplot represents median; the top and the bottom of a box represent upper quartile and lower quartile, respectively; bars at the top and the bottom show the maximum and minimum, respectively, after excluding the abnormal values. Antibiotic group and control are shown in red and blue, respectively. Hollow circles indicate the abnormal values. Day 0 means the endpoint of 8-day antibiotic treatment. For statistical analysis, normality test and homogeneity test of variance were performed. If it met the parameter test conditions, t-test was performed; otherwise, the Wilcoxon rank sum test was carried out. *P < 0.05 indicates statistic difference. Figure S2. Rarefaction curves in microbial diversity analysis. A and C represent antibiotic group and control, respectively. M is short for month. Figure S3. Alluvial diagrams of species composition across time. The data are from 16S rRNA gene sequencing. A and C represent antibiotic group and control, respectively. The figures were produced using the R package (version 4.1.2). Each column indicates species composition proportion. In b the top 20 genera with relative abundance are shown. Other genera not ranking in the top 20 are combined and named as “other”. Figure S4. Species difference analysis at the genus level using the ALDEx2 tool. The data are from 16S rRNA gene sequencing. A and C represent antibiotic group and control, respectively. M is short for month. The screening criteria of differential species are as follows: absolute value of “Effect” > 1; FDR < 0.05. Only the 1st (a), 2nd (b), 3rd (c), and 7th (d) months contain differential species at the genus level. Figure S5. Visualization of network modules. The data analysis is based on 16S rRNA gene sequencing. A and C represent antibiotic group and control, [file 40168_2024_1795_MOESM2_ESM.docx]

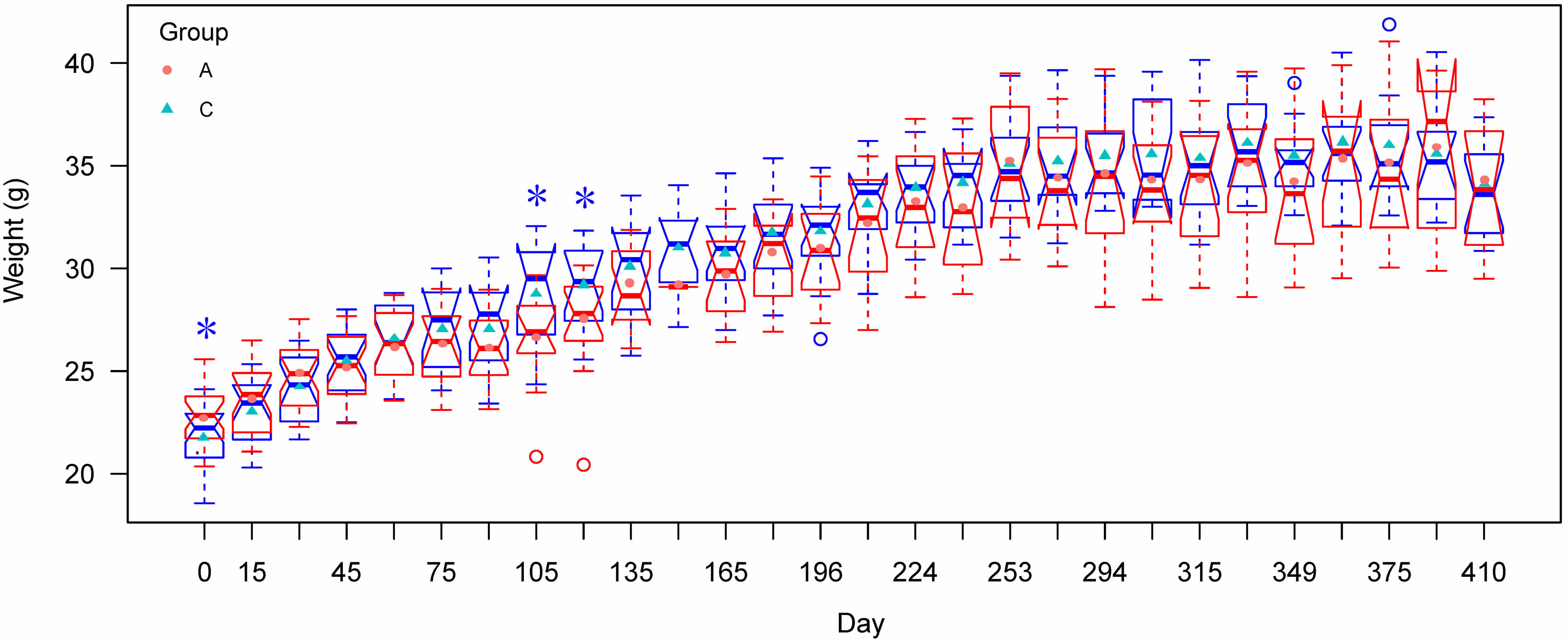


**Supplementary Fig. S1.** Weight changes of mice over time. Triangles and solid dots indicate the mean weight of mice. A and C represent antibiotic group and control, respectively. The middle of boxplot represents median; the top and the bottom of a box represent upper quartile and lower quartile, respectively; bars at the top and the bottom show the maximum and minimum, respectively, after excluding the abnormal values. Antibiotic group and control are shown in red and blue, respectively. Hollow circles indicate the abnormal values. Day 0 means the end point of 8-days antibiotic treatment. For statistical analysis, normality test and homogeneity test of variance were performed. If it met the parameter test conditions, *t*-test was performed; otherwise, the Wilcoxon rank sum test was carried out. **P* < 0.05 indicates statistic difference.


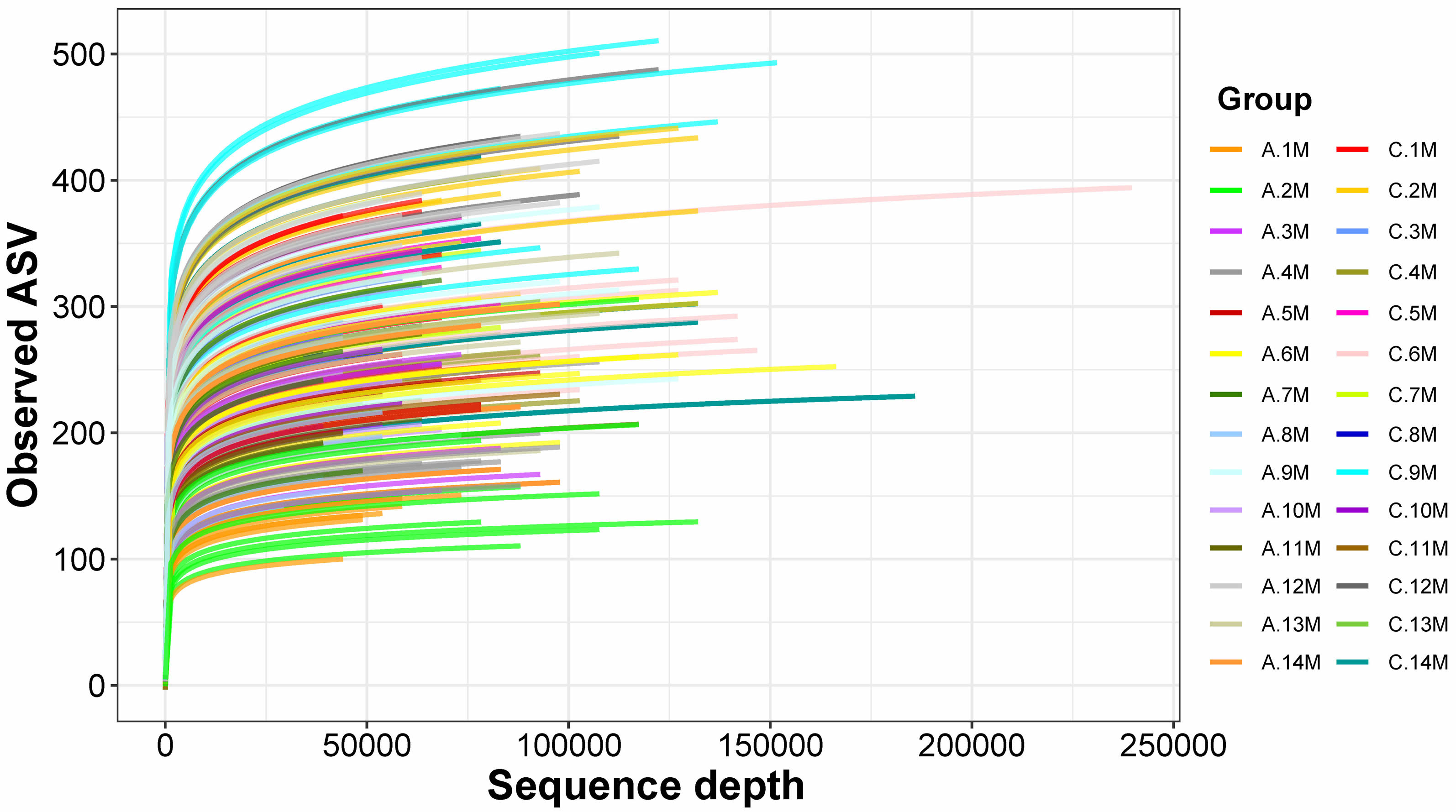


**Supplementary Fig. S2.** Rarefaction curves in microbial diversity analysis. A and C represent antibiotic group and control, respectively. M is short for month.


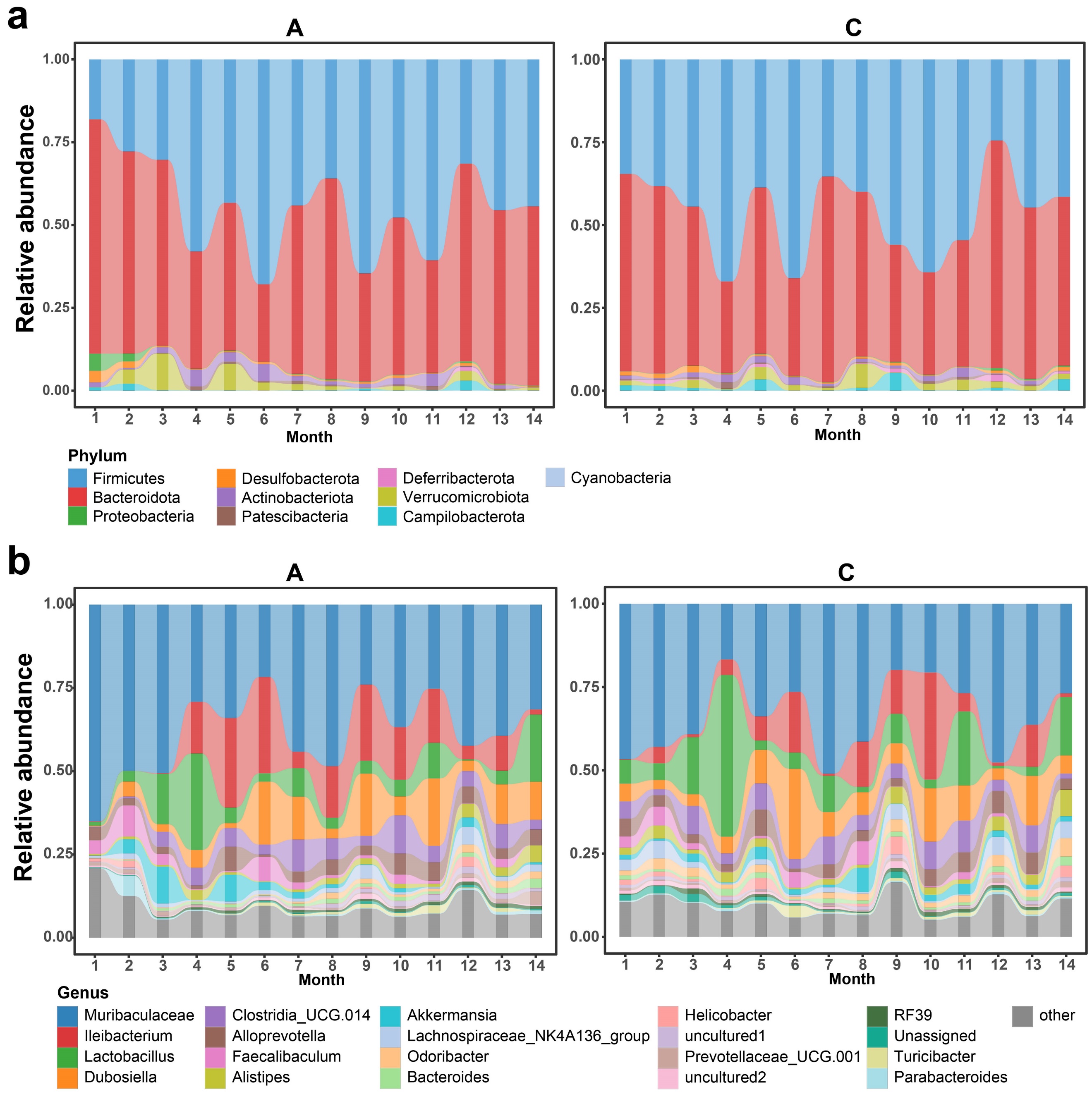


**Supplementary Fig. S3.** Alluvial diagrams of species composition across time. The data are from 16S rRNA gene sequencing. A and C represent antibiotic group and control, respectively. The figures were produced using the R package (version 4.1.2). Each column indicates species composition proportion. In **b** the top 20 genera with relative abundance are shown. Other genera not ranking in the top 20 are combined and named as “other”.


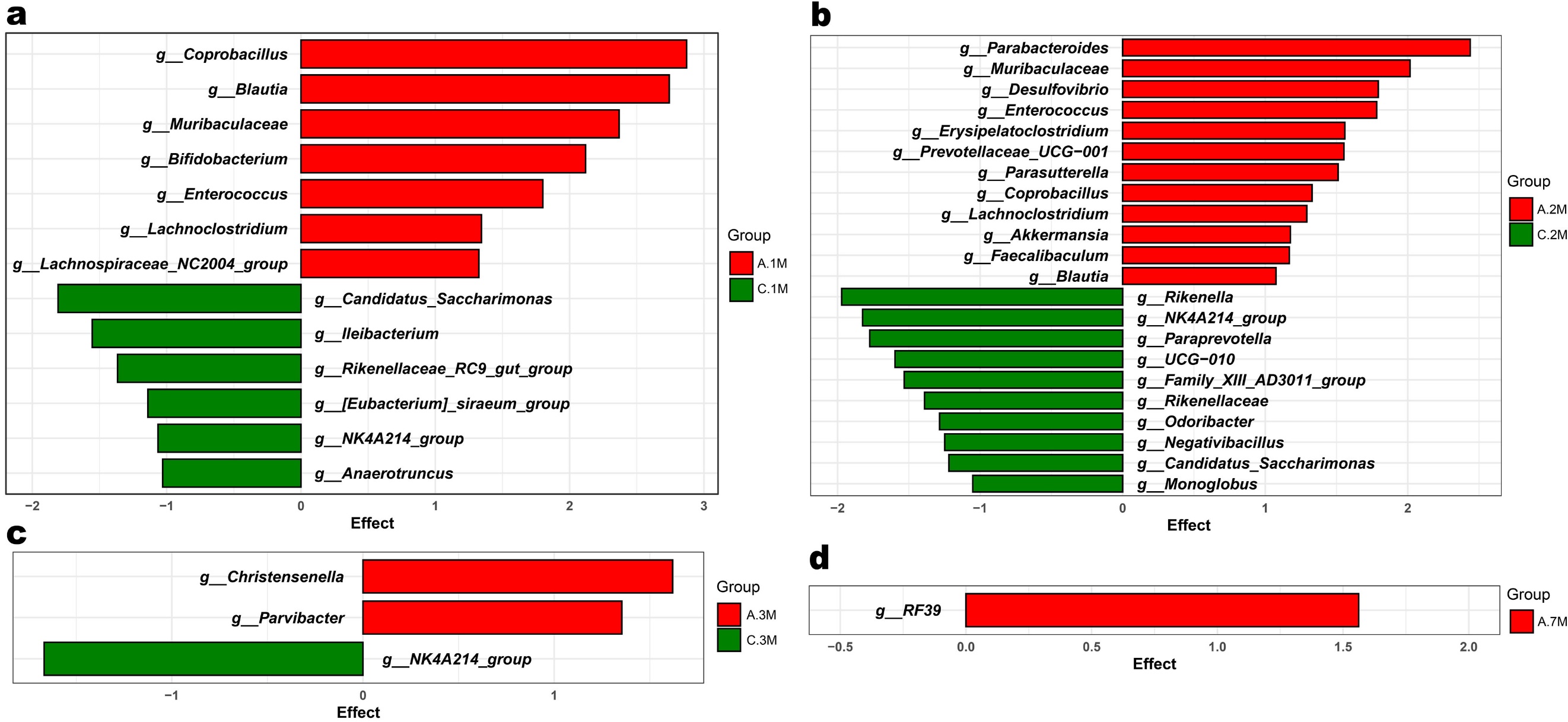


**Supplementary Fig. S4.** Species difference analysis at the genus level using the ALDEx2 tool. The data are from 16S rRNA gene sequencing. A and C represent antibiotic group and control, respectively. M is short for month. The screening criteria of differential species are as follows: absolute value of “Effect” > 1; FDR < 0.05. Only the 1^st^ (**a**), 2^nd^ (**b**), 3^rd^ (**c**), and 7^th^ (**d**) months contain differential species at the genus level.


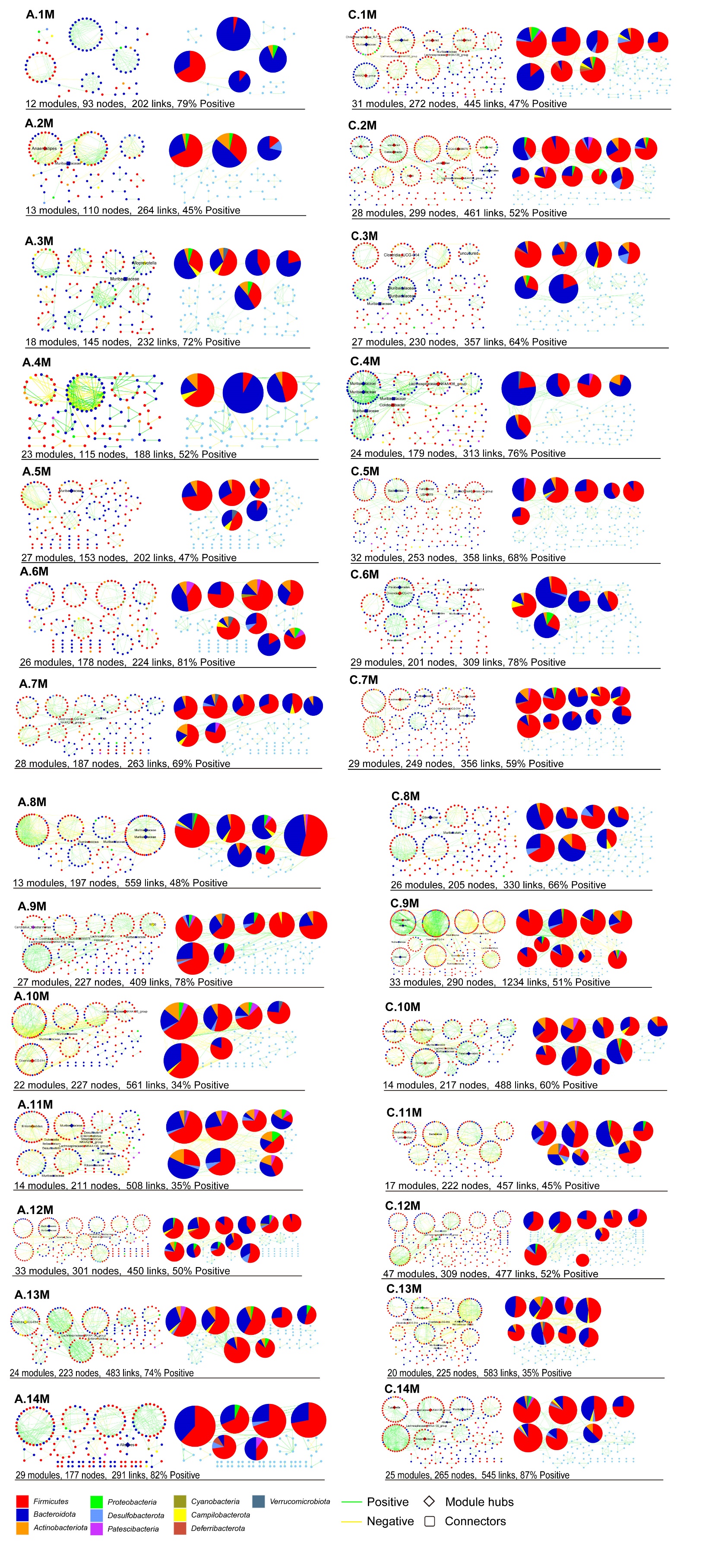


**Supplementary Fig. S5.** Visualization of network modules. The data analysis is based on 16S rRNA gene sequencing. A and C represent antibiotic group and control, respectively. M is short for month. The number of modules, nodes, links and positive links are shown. The left side of each panel shows the schematic diagram of each module, while the right side (pie chart) shows the microbial composition (at the phylum level) of each corresponding module. Pie charts show the modules with >10 nodes. Nodes with different colors in the modules represent different microbes at the phylum levels. Module hubs and connectors are shown in the modules. Green line and yellow line indicate positive and negative associations, respectively.


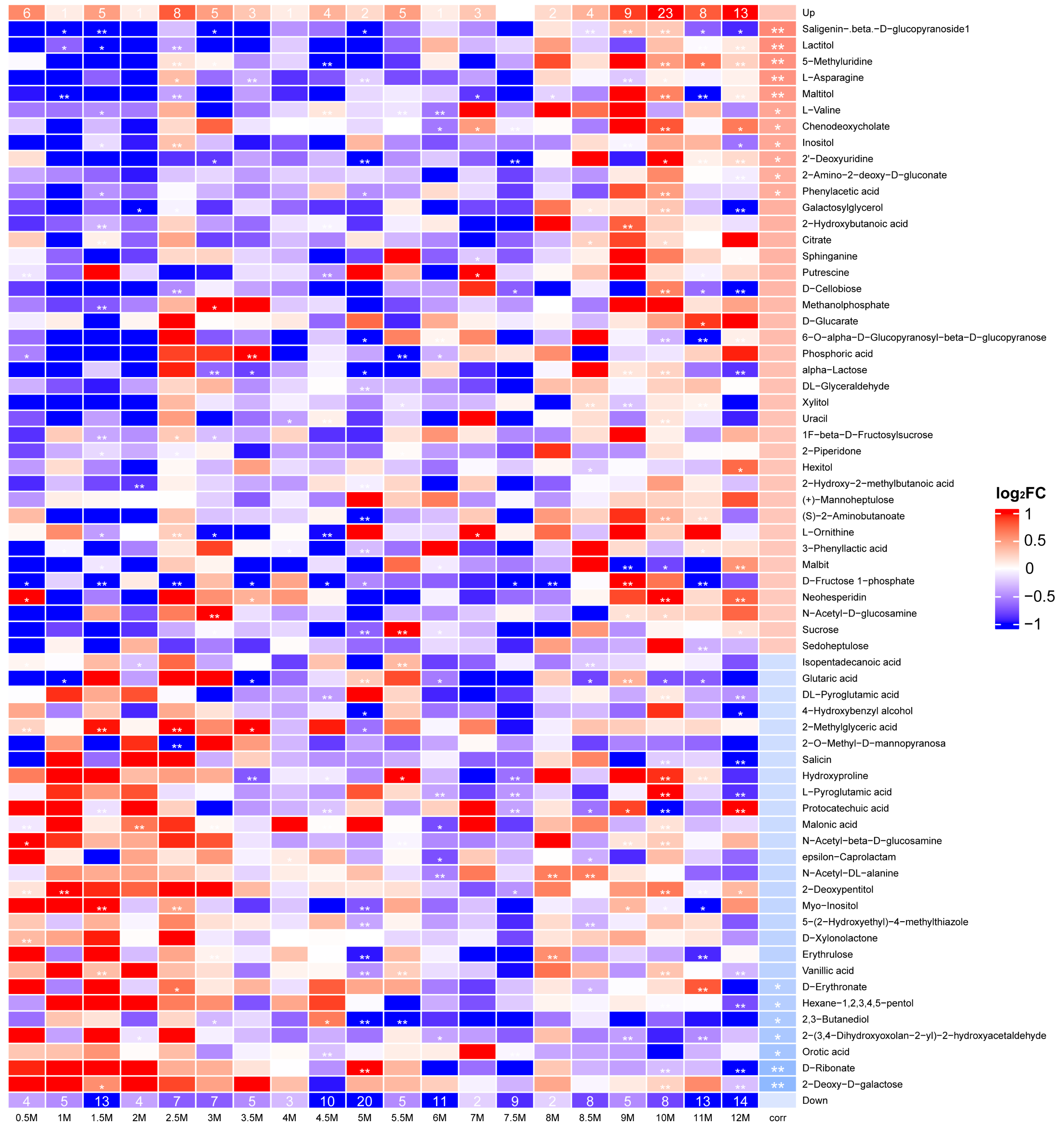


**Supplementary Fig. S6.** Differential metabolites identified by metabolomics analysis. Horizontal direction indicates sampling time. M is short for month. FC is short for fold change (A/C, where A and C represent antibiotic group and control, respectively). Grids from the last column indicate the correlation between FC values of differential metabolites and sampling time, with red and blue representing positive and negative correlations, respectively. In other grids, red and blue indicate the differential metabolite with higher abundance in antibiotic group and control, respectively. The Arabic numbers in the first (marked by “Up”) and last (marked by “Down”) lines represent the number of up-regulated and down-regulated metabolites at each time point, respectively. ******P* < 0.05; *******P* < 0.01.
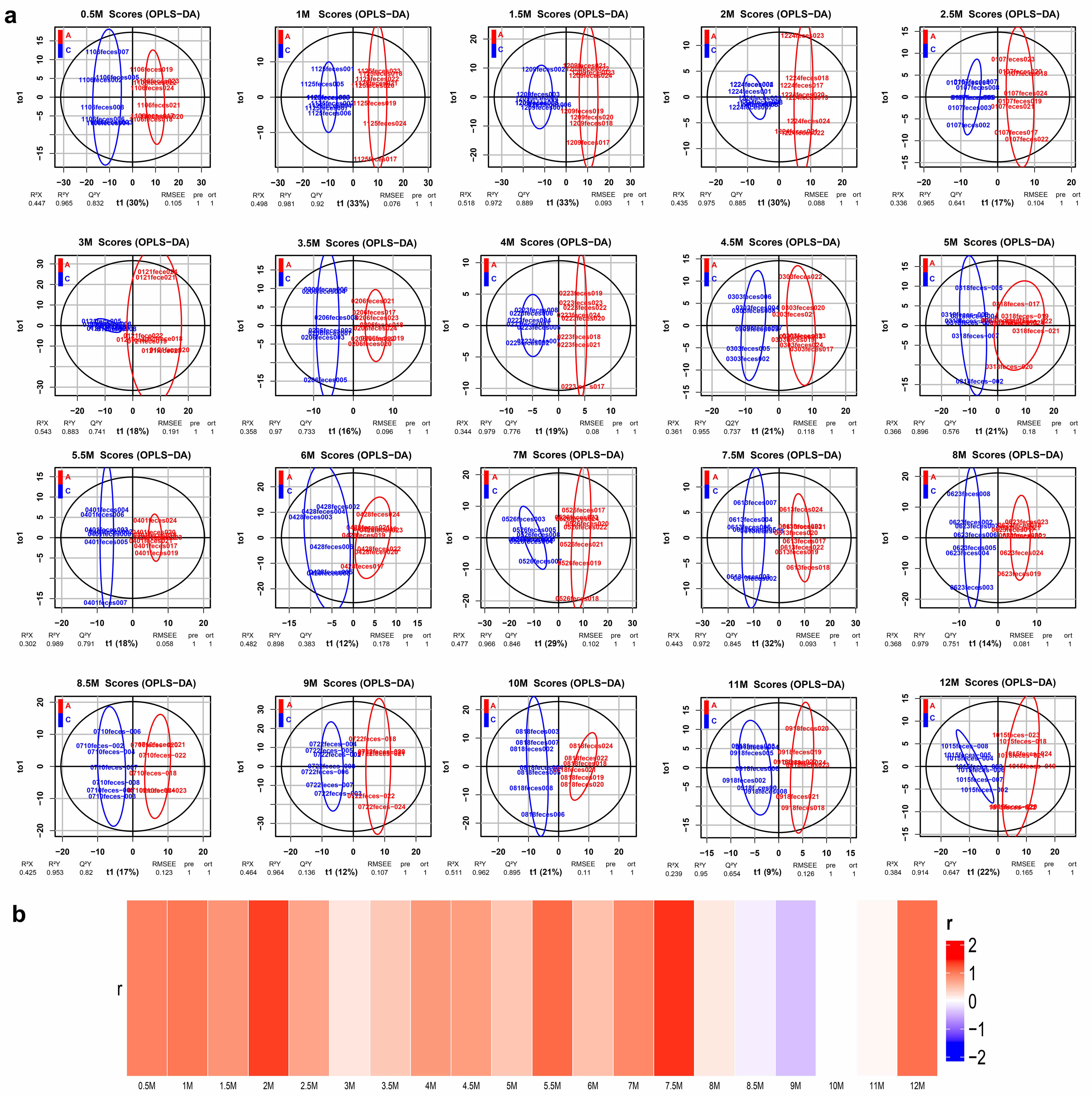


**Supplementary Fig. S7.** Differential analysis of metabolites between ceftriaxone group and control. The analysis was performed using the orthogonal to partial least squares discriminant analysis (OPLS-DA). **a** Samples from antibiotic group and control are represented by red and blue, respectively. Horizontal direction indicates the differences between groups, and longitudinal direction represents the differences within a single group. **b** r < 0 indicates that the difference between groups is less than the difference within a single group; r > 0 indicates that the difference between groups is larger than the difference within a single group.
