# Supplementary figures and images for: Short-term exposure to antibiotics begets long-term disturbance in gut microbial metabolism and molecular ecological networks
Source: Microbiome. 2024 May 7;12:80. doi: 10.1186/s40168-024-01795-z (PMC11075301; doi:10.1186/s40168-024-01795-z)

a

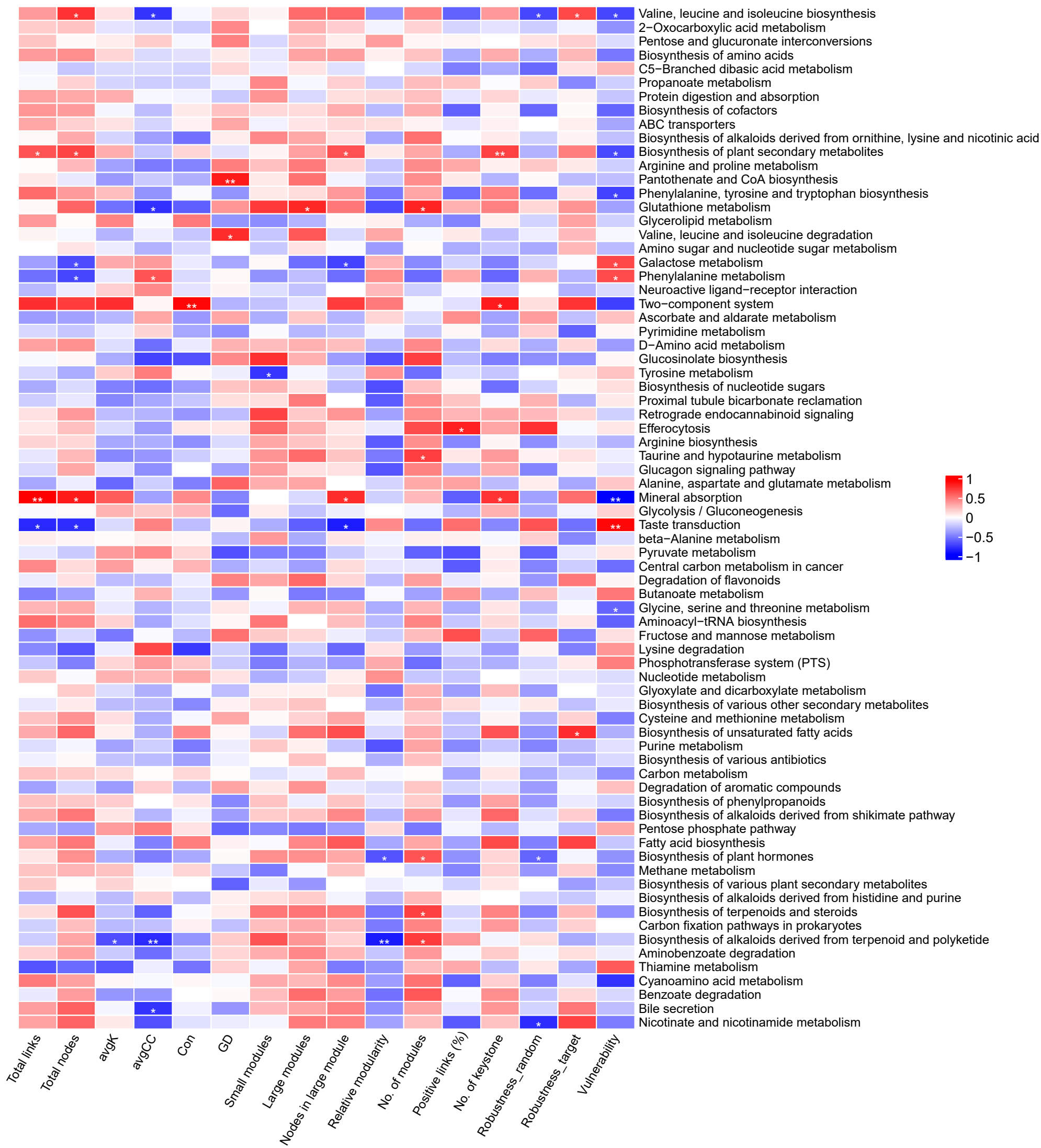

b

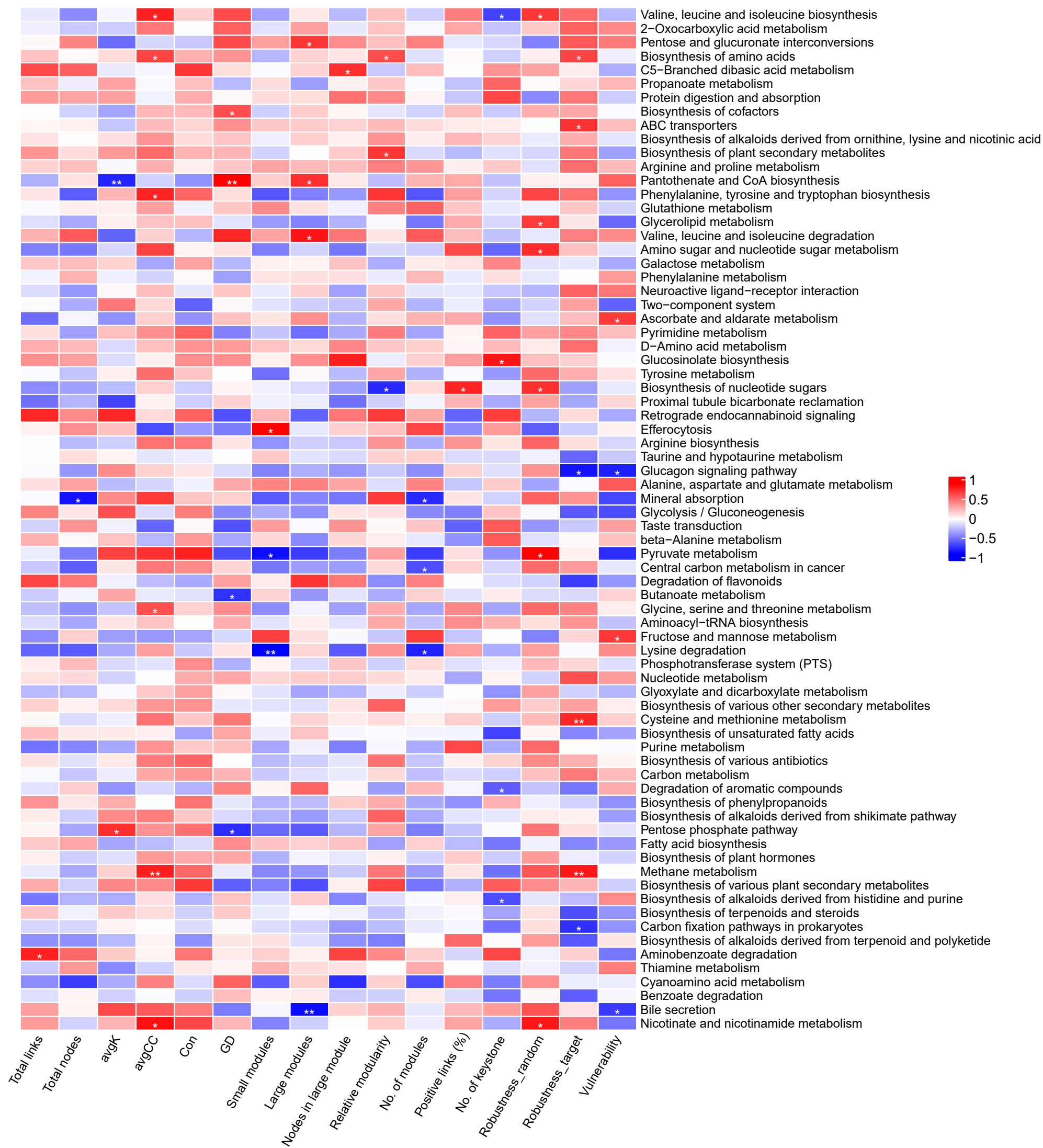

Supplement: Supplementary file 4 — Additional file 3: Supplementary Figure S8. Correlations between network parameters and metabolic pathways with significant differences. The network parameters are from the data of microbial MENs. Correlations for antibiotic group and control are shown in a and b, respectively. The enriched pathways were produced by annotation of differential metabolites in the KEGG database (organism group: bacteria). The correlations were carried out between –lg(P value of pathway enrichment) and network parameters, generating correlation coefficients and corresponding P values. Red and blue represent positive and negative correlations, respectively. avgK, average K; avgCC, average clustering coefficient; Con, connectedness; GD, average path distance. *P < 0.05; **P < 0.01. [file 40168_2024_1795_MOESM3_ESM.pdf]

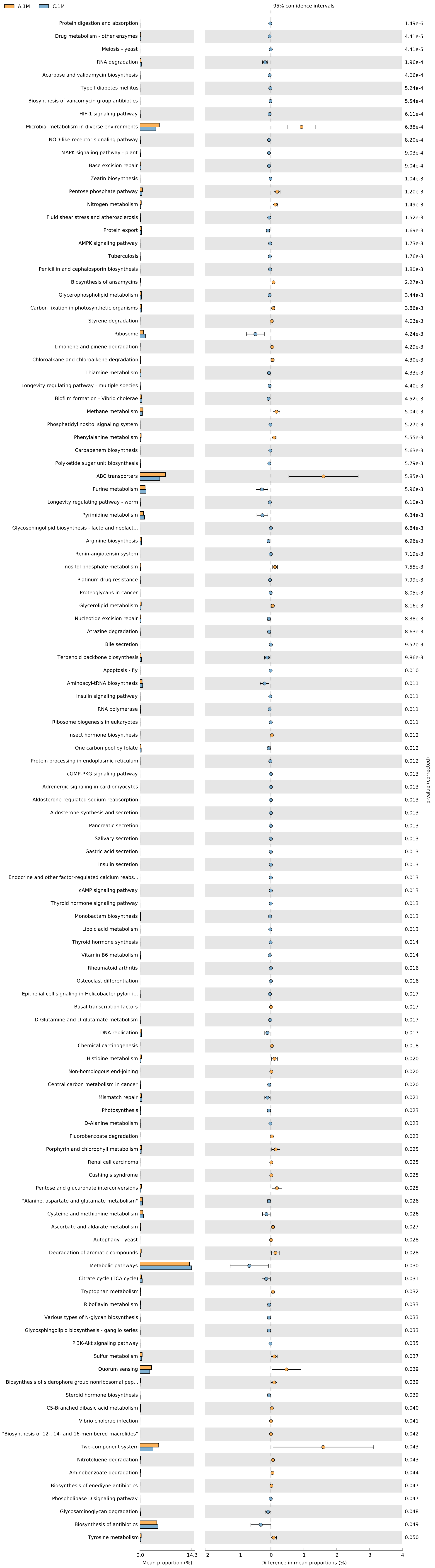

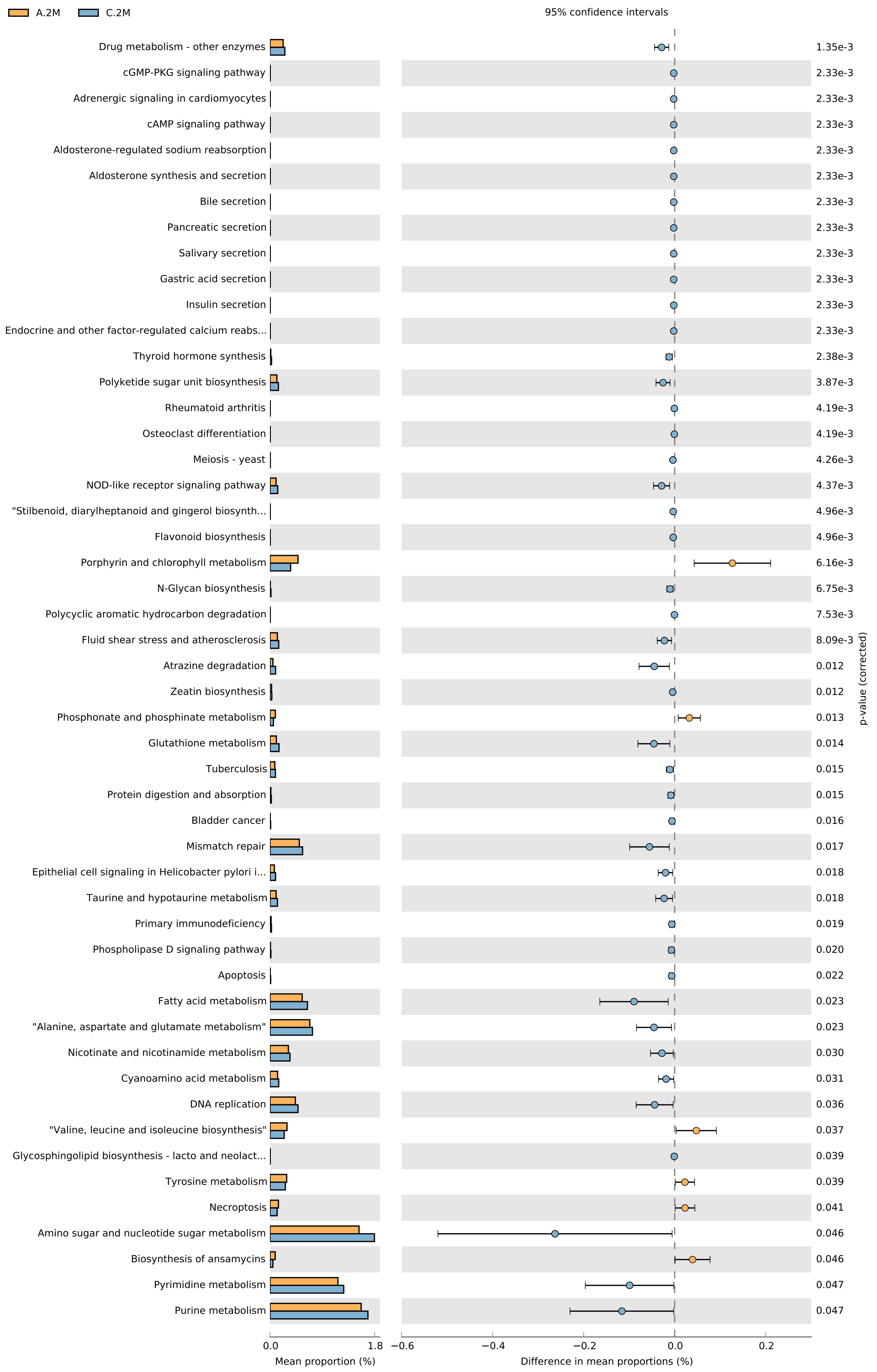

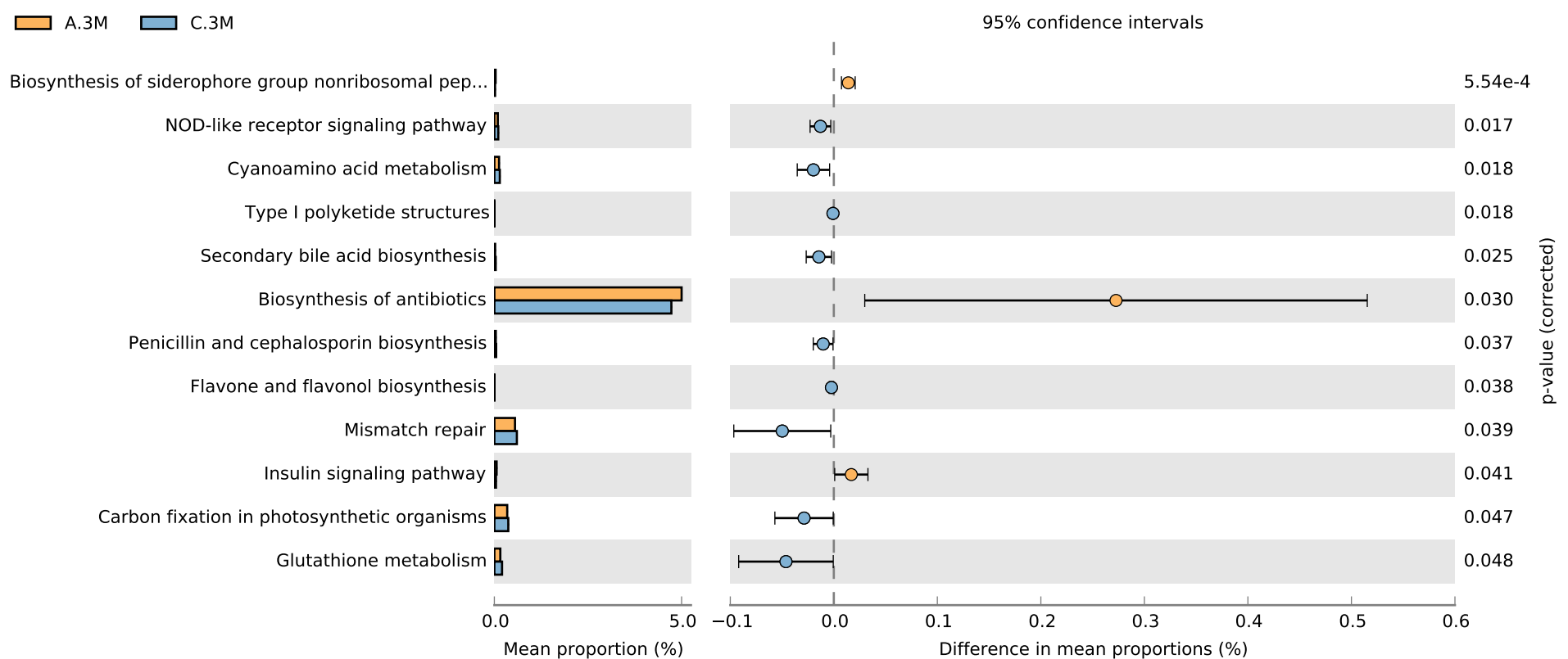

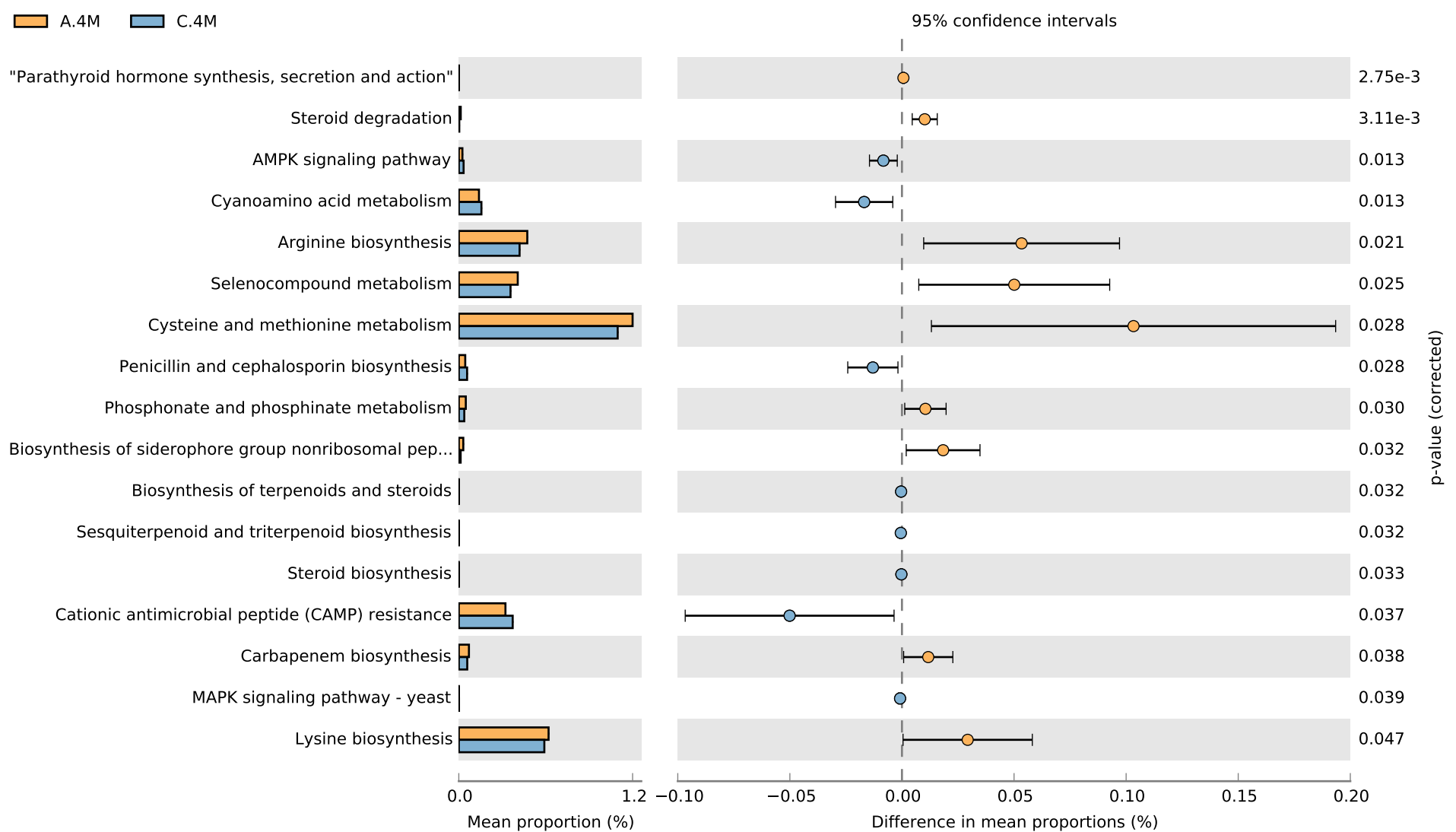

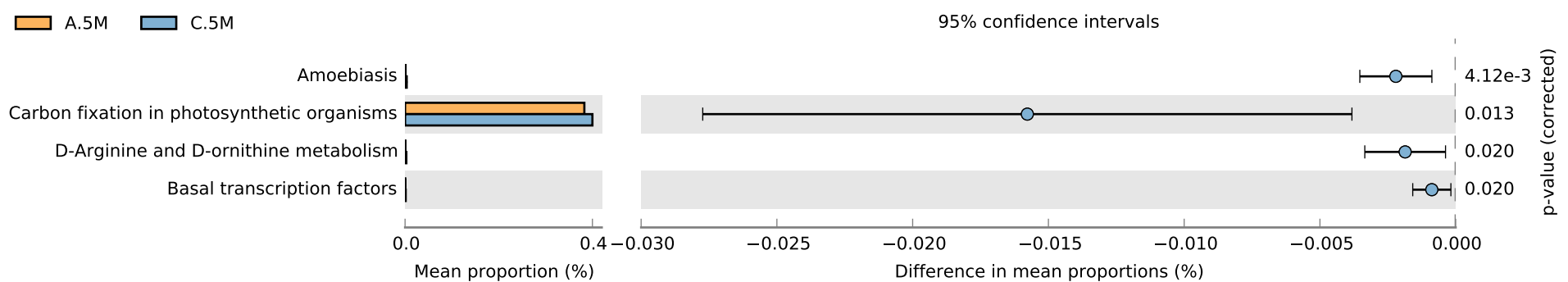

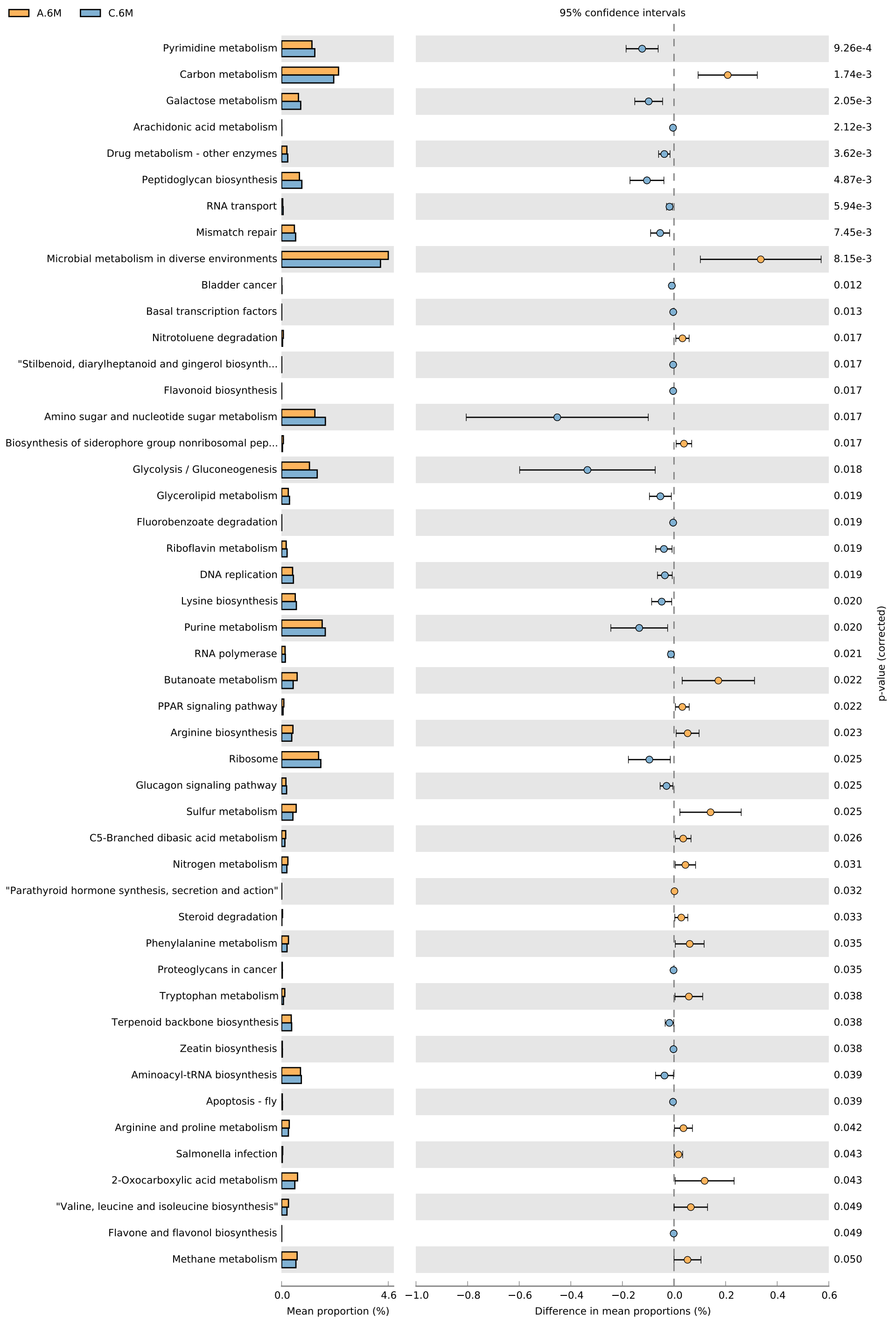

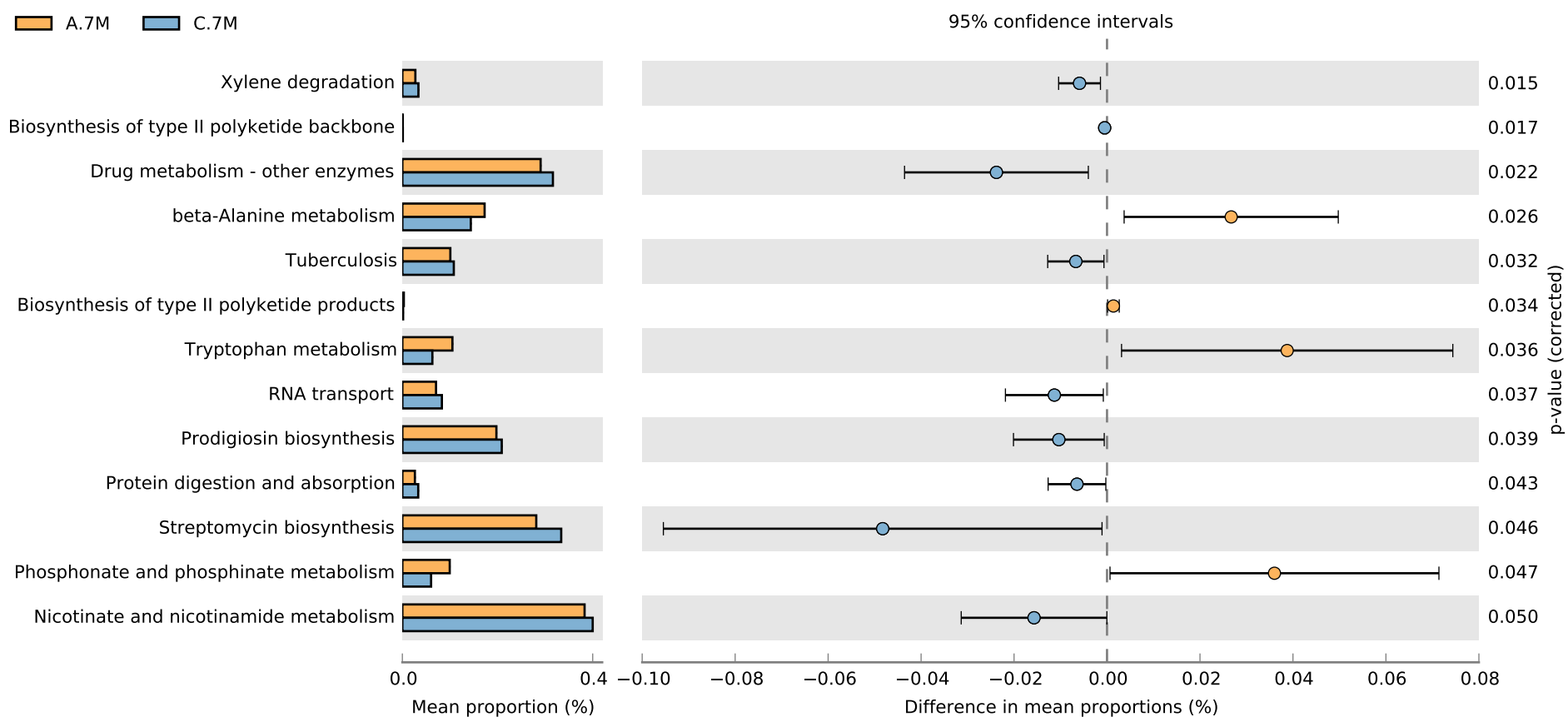

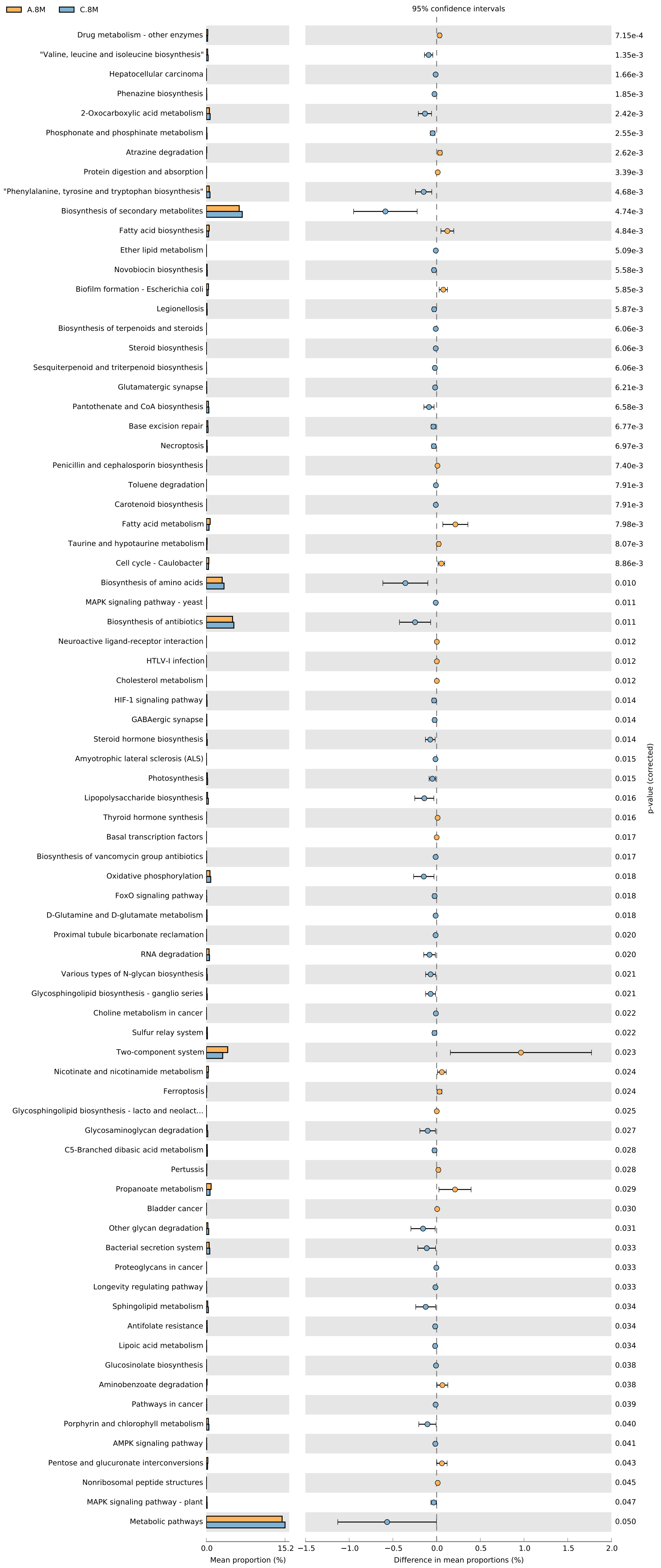

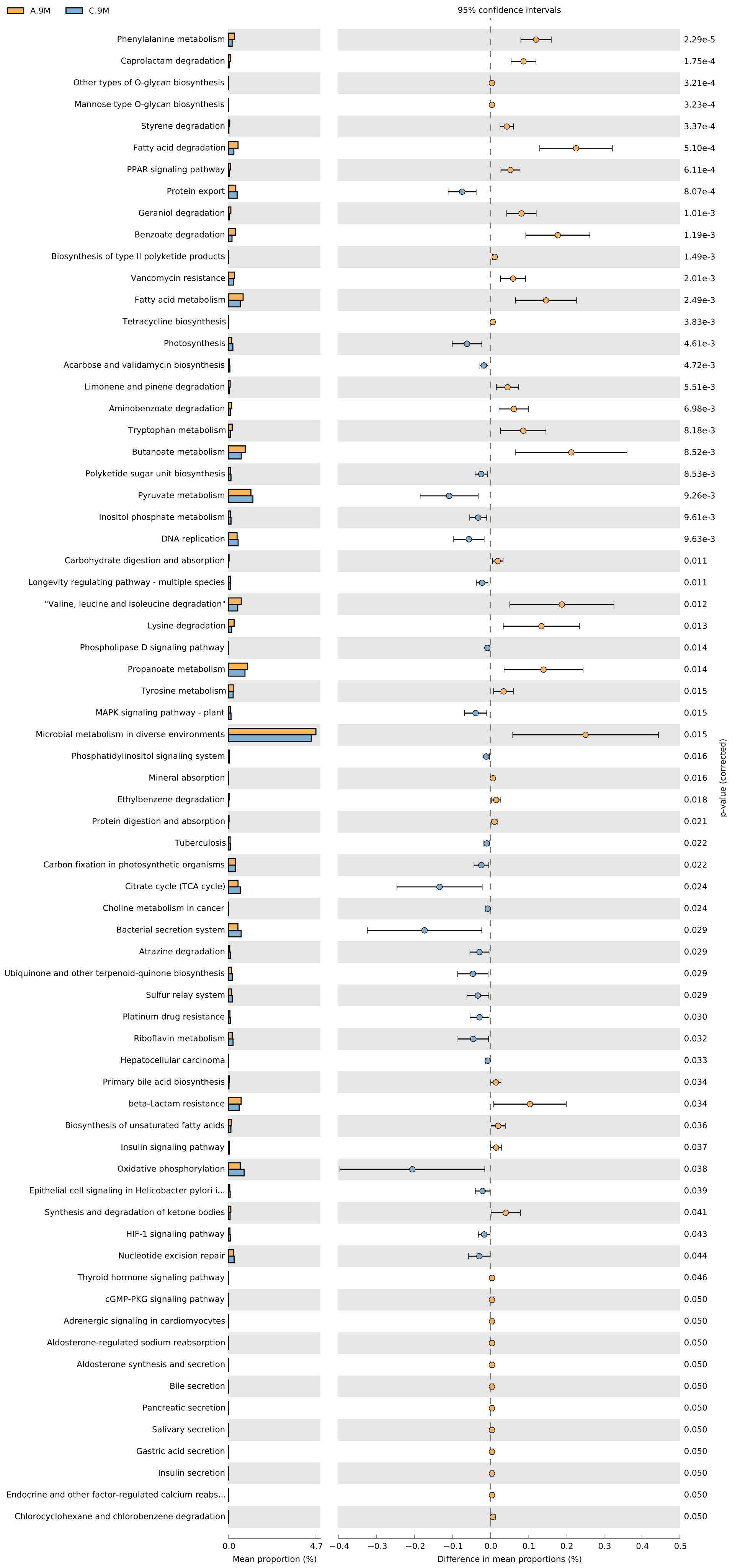

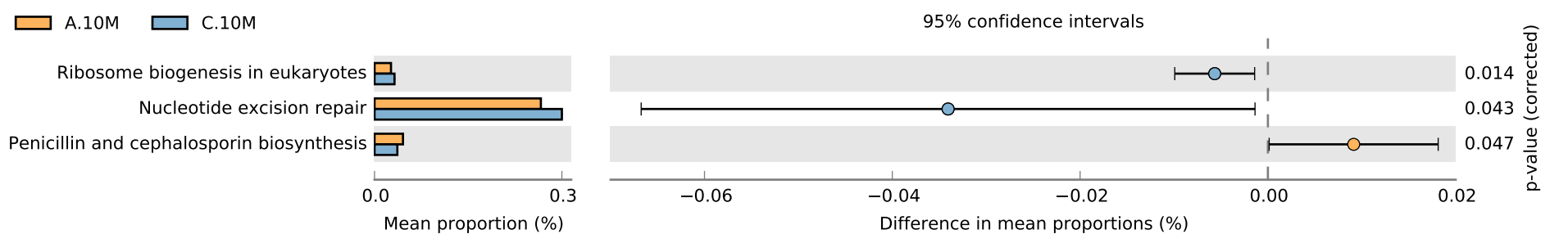

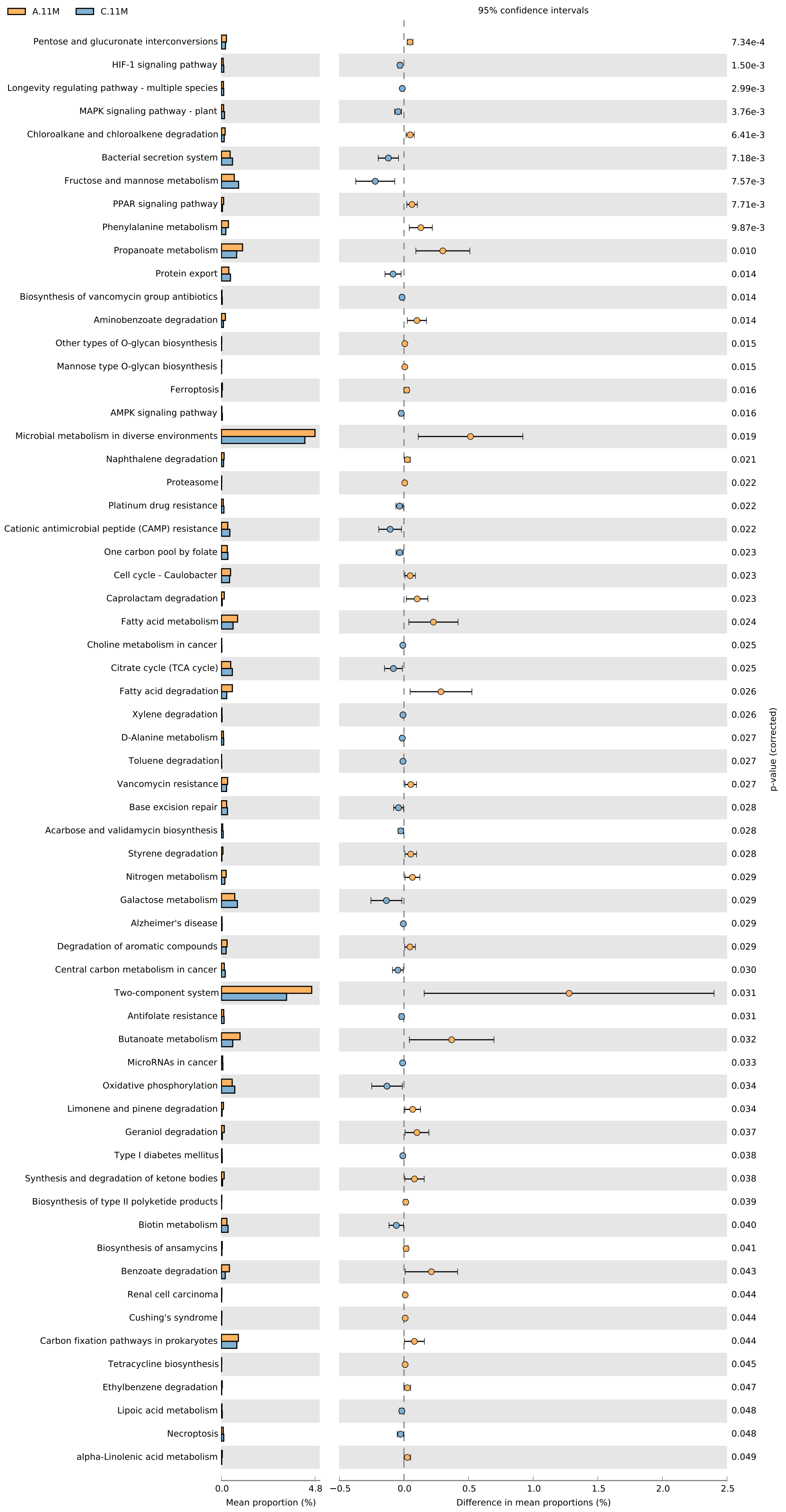

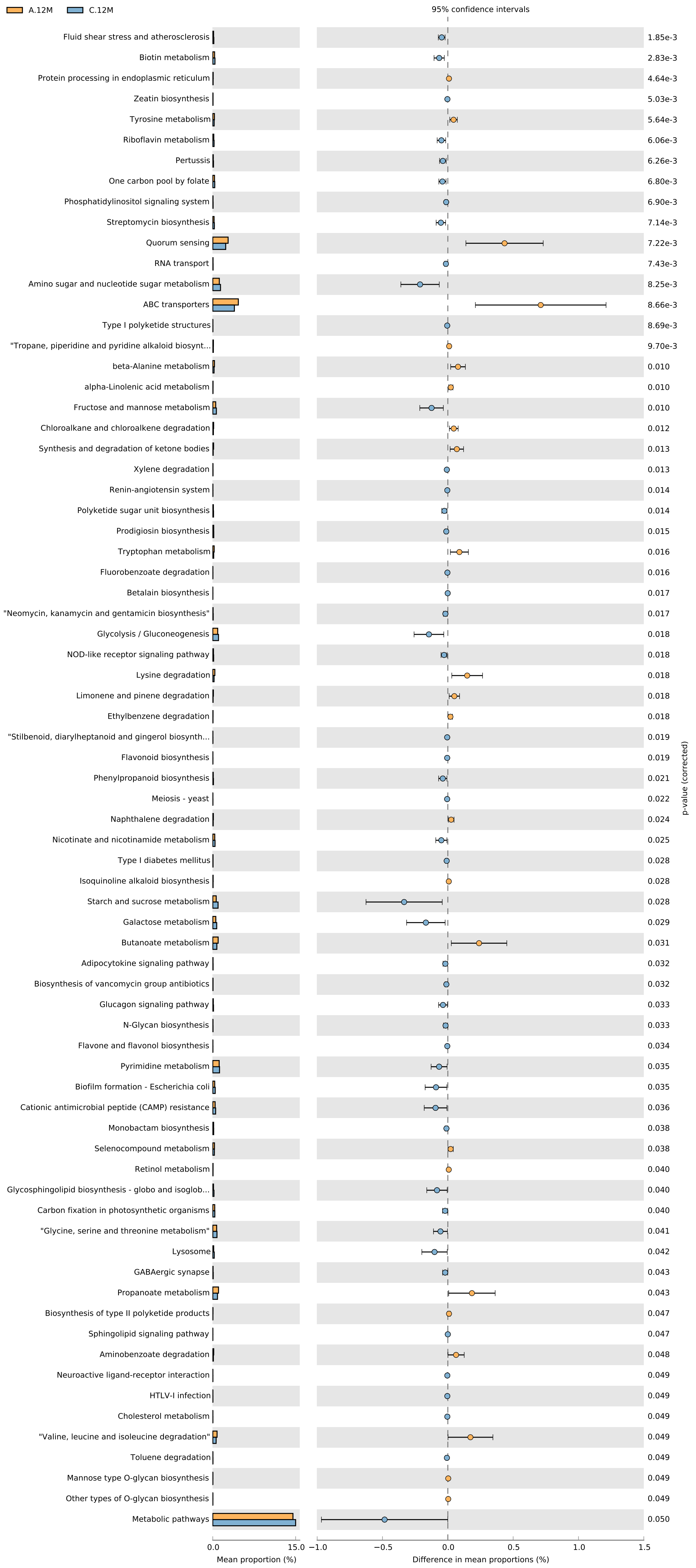

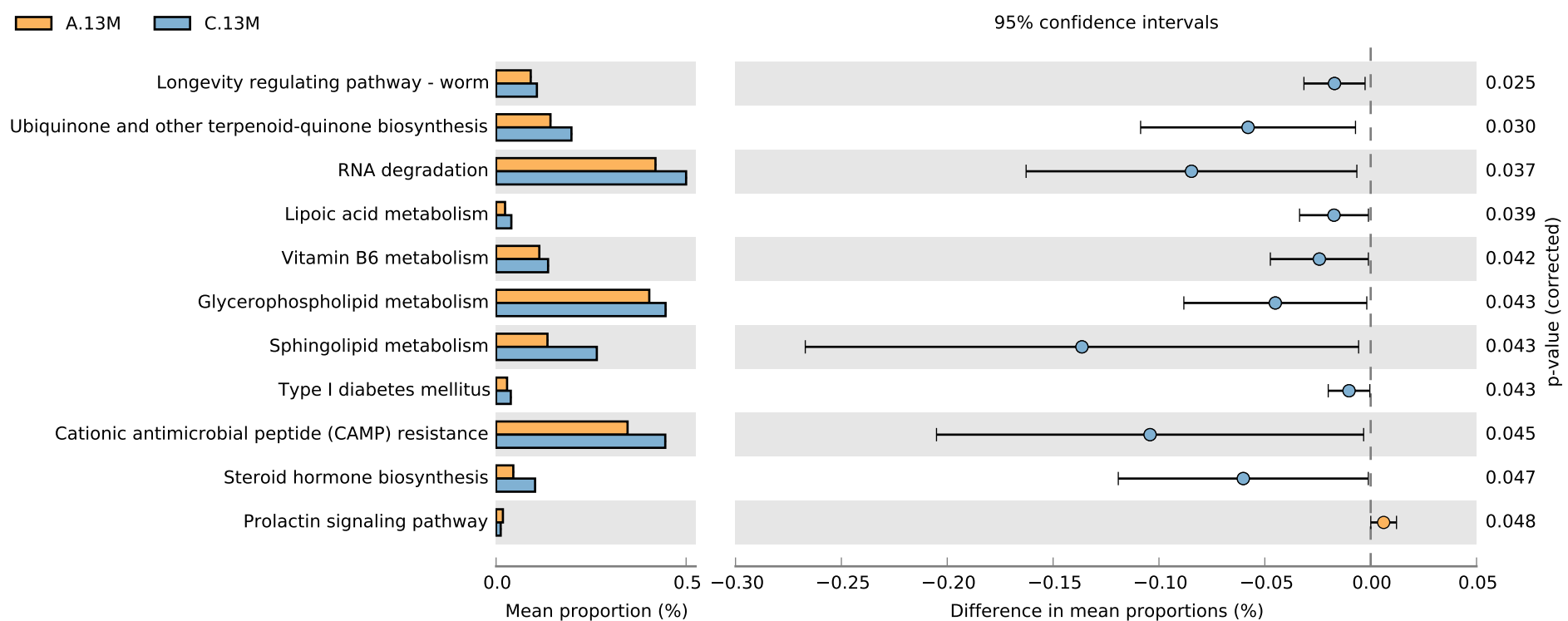

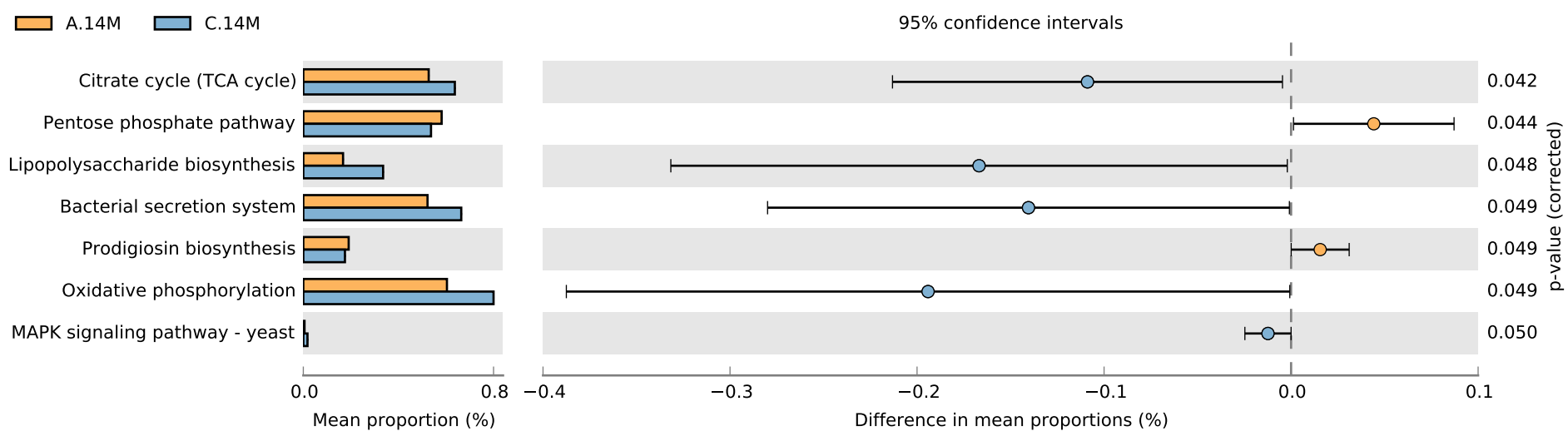

Supplement: Supplementary file 5 — Additional file 4: Supplementary Figure S9. Metabolic function prediction on KEGG level_3 category based on the data of 16S rRNA gene sequencing. The metabolic function potential of microbial communities was predicted using the Tax4fun2 in R package and the STAMP software. A and C represent antibiotic group and control, respectively. 1M means the 1st month, and so on. Pathways with corrected P values less than or equal to 0.05 are shown. [file 40168_2024_1795_MOESM4_ESM.pdf]

A

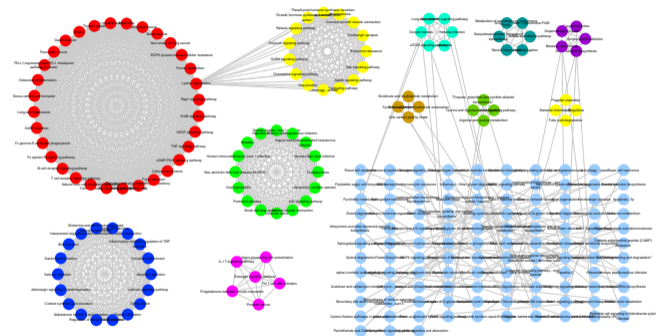

C

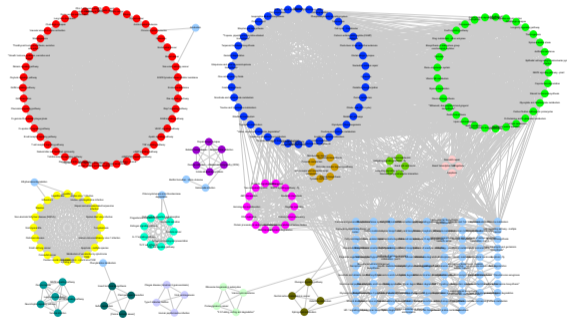

Supplement: Supplementary file 8 — Additional file 7: Supplementary Figure S12. Functional networks based on metagenomic sequencing data from the 12th month samples. A and C represent antibiotic group and control, respectively. Pathways and the correlation between pathways are represented by nodes and edges, respectively. The metabolic pathways were annotated at the level_3 categories in the KEGG database. In each subfigure different subnetworks are represented by different colors. Yet, it does not mean that in groups A and C the same colour indicate the same subnetwork. The unclustered pathways are displayed in the grid layout. [file 40168_2024_1795_MOESM7_ESM.pdf]

A

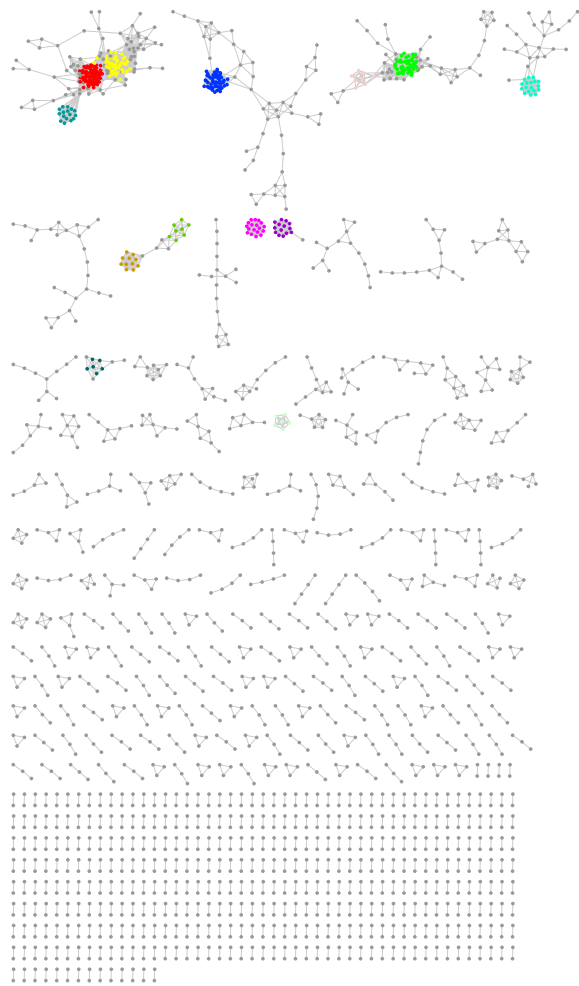

C

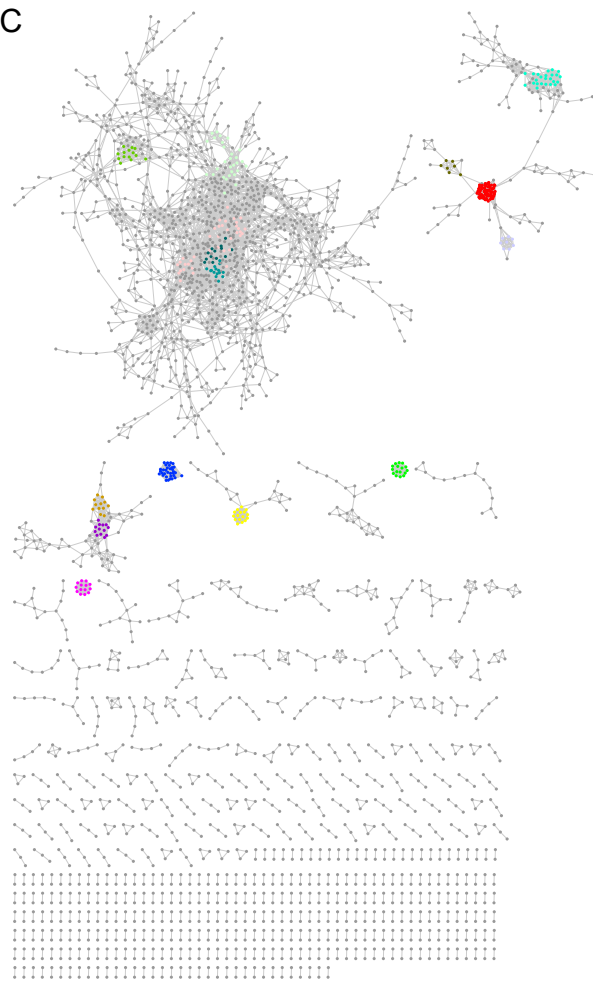

Supplement: Supplementary file 9 — Additional file 8: Supplementary Figure S13. Functional networks constructed using KO number (protein/enzyme) and the correlation between KO as nodes and edges (links), respectively. The networks were constructed based on metagenomic sequencing data. Core subnetworks were extracted mainly based on the following parameters: degree cutoff 2; K-core 5; Max. depth 100. A and C represent antibiotic group and control, respectively. In each subfigure different subnetworks are represented by different colors. [file 40168_2024_1795_MOESM8_ESM.pdf]
