# Supplementary material for: Short-term exposure to antibiotics begets long-term disturbance in gut microbial metabolism and molecular ecological networks
Source: Microbiome. 2024 May 7;12:80. doi: 10.1186/s40168-024-01795-z (PMC11075301; doi:10.1186/s40168-024-01795-z)
Supplement: Supplementary file 6 — Additional file 5: Supplementary Figure S10. Metabolic function prediction on KEGG level_2 category based on the data of 16S rRNA gene sequencing. A and C represent antibiotic group and control, respectively. 1M means the 1st month, and so on. Pathways with corrected P values less than or equal to 0.05 are shown. There is not any pathway with significant difference in the 5th or 10th month. [file 40168_2024_1795_MOESM5_ESM.pdf]

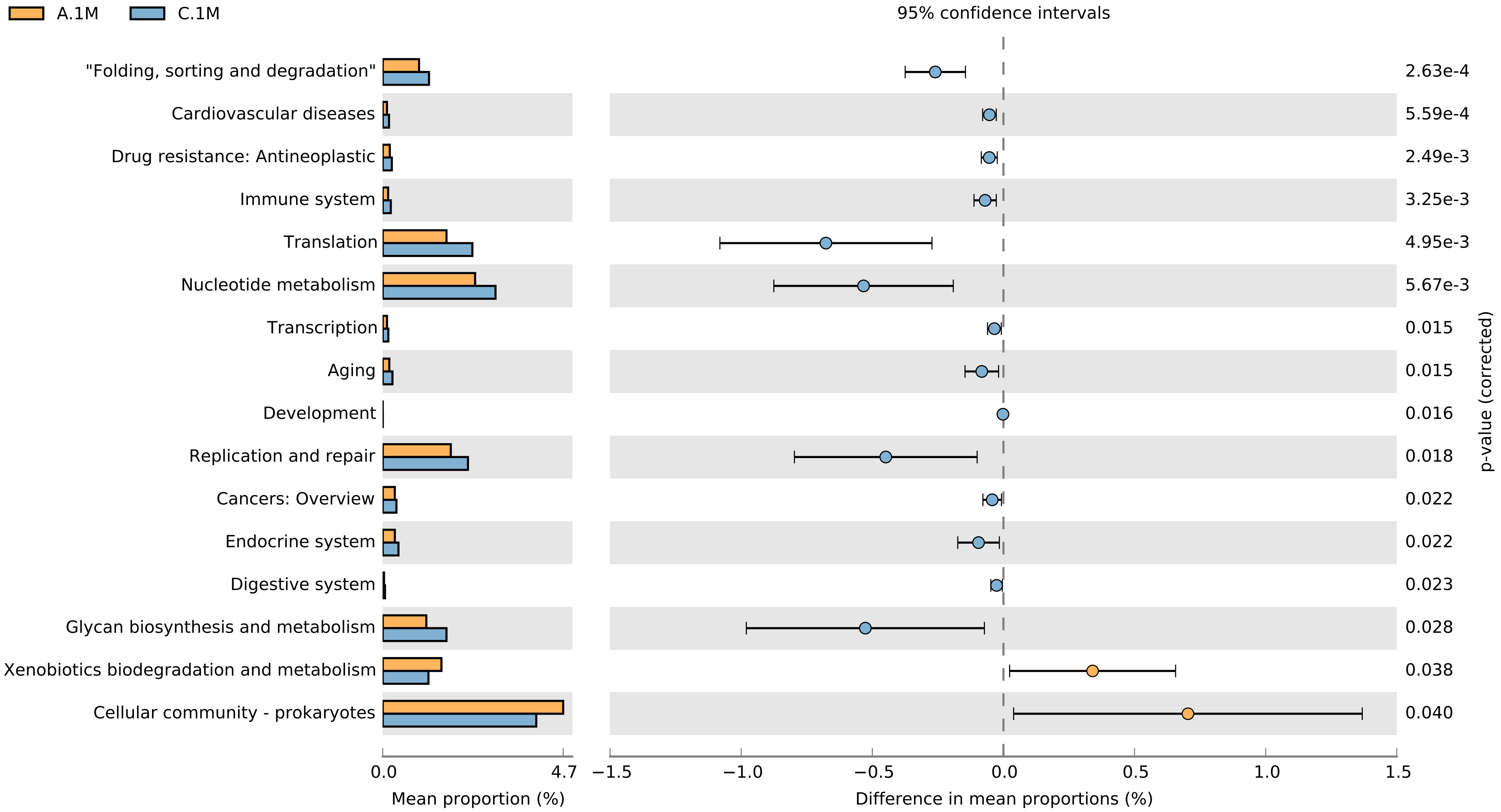
A.1M
C.1M
95% confidence intervals
"Folding, sorting and degradation"
Cardiovascular diseases
Drug resistance: Antineoplastic
Immune system
Translation
Nucleotide metabolism
Transcription
Aging
Development
Replication and repair
Cancers: Overview
Endocrine system
Digestive system
Glycan biosynthesis and metabolism
Xenobiotics biodegradation and metabolism
Cellular community - prokaryotes
2.63e-4
5.59e-4
2.49e-3
3.25e-3
4.95e-3
5.67e-3
0.015
0.015
0.016
0.018
0.022
0.022
0.023
0.028
0.038
0.040
p-value (corrected)
0.0
4.7
Mean proportion (%)
−1.5
−1.0
−0.5
0.0
0.5
1.0
1.5
Difference in mean proportions (%)

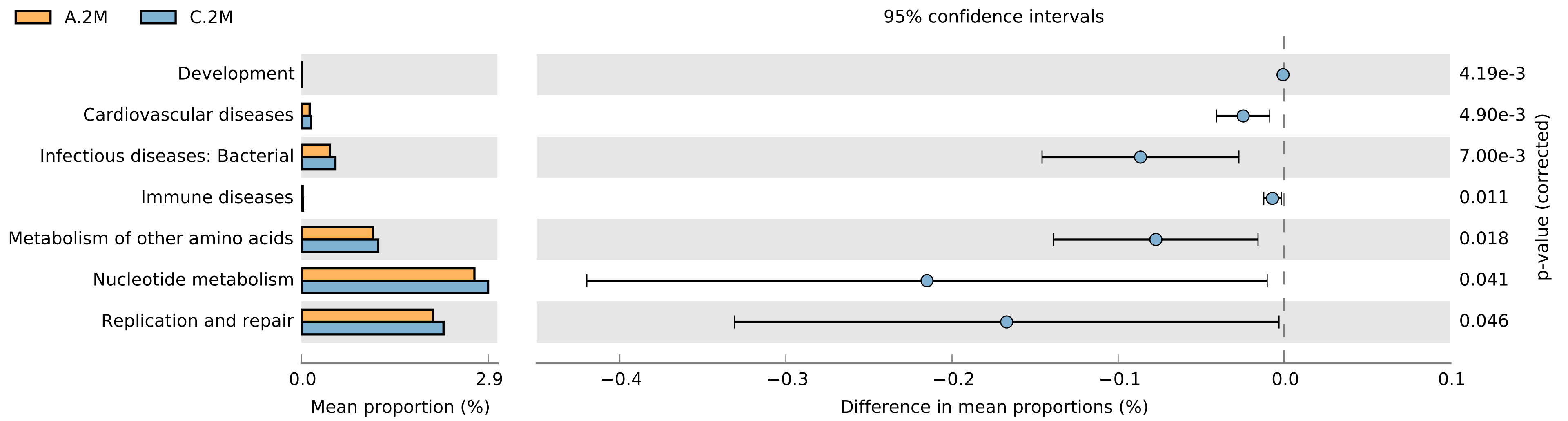
A.2M
C.2M
95% confidence intervals
Development
Cardiovascular diseases
Infectious diseases: Bacterial
Immune diseases
Metabolism of other amino acids
Nucleotide metabolism
Replication and repair
4.19e-3
4.90e-3
7.00e-3
0.011
0.018
0.041
0.046
p-value (corrected)
0.0
2.9
Mean proportion (%)
−0.4
−0.3
−0.2
−0.1
0.0
0.1
Difference in mean proportions (%)

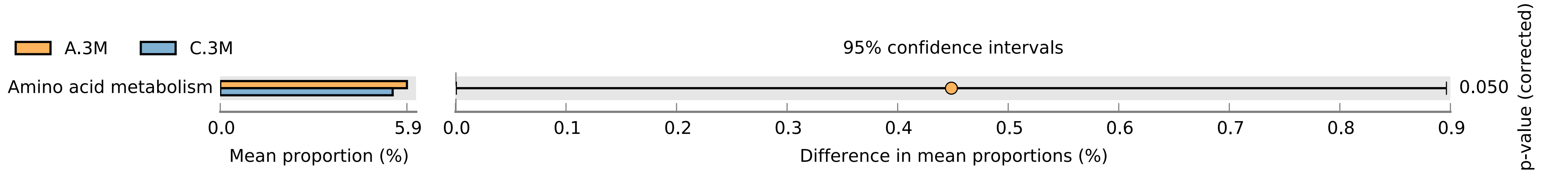
A.3M
C.3M
95% confidence intervals
Amino acid metabolism
0.050
0.0
5.9
Mean proportion (%)
0.0
0.1
0.2
0.3
0.4
0.5
0.6
0.7
0.8
0.9
Difference in mean proportions (%)
p-value (corrected)

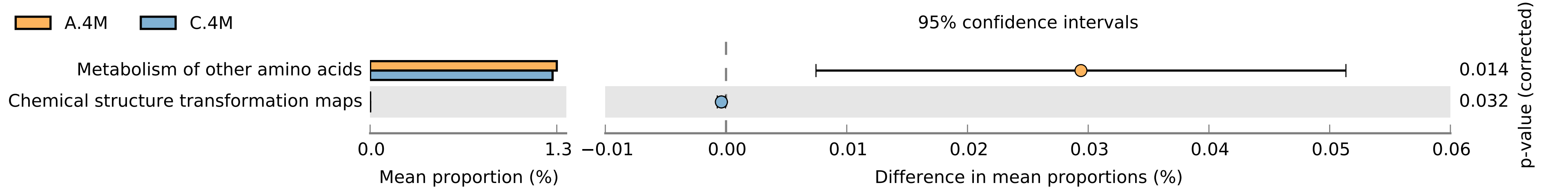

A.4M
C.4M
95% confidence intervals
Metabolism of other amino acids
Chemical structure transformation maps
0.014
0.032
p-value (corrected)
0.0
1.3
−0.01
0.00
0.01
0.02
0.03
0.04
0.05
0.06
Mean proportion (%)
Difference in mean proportions (%)

5M (There is not any pathway with significant difference.)

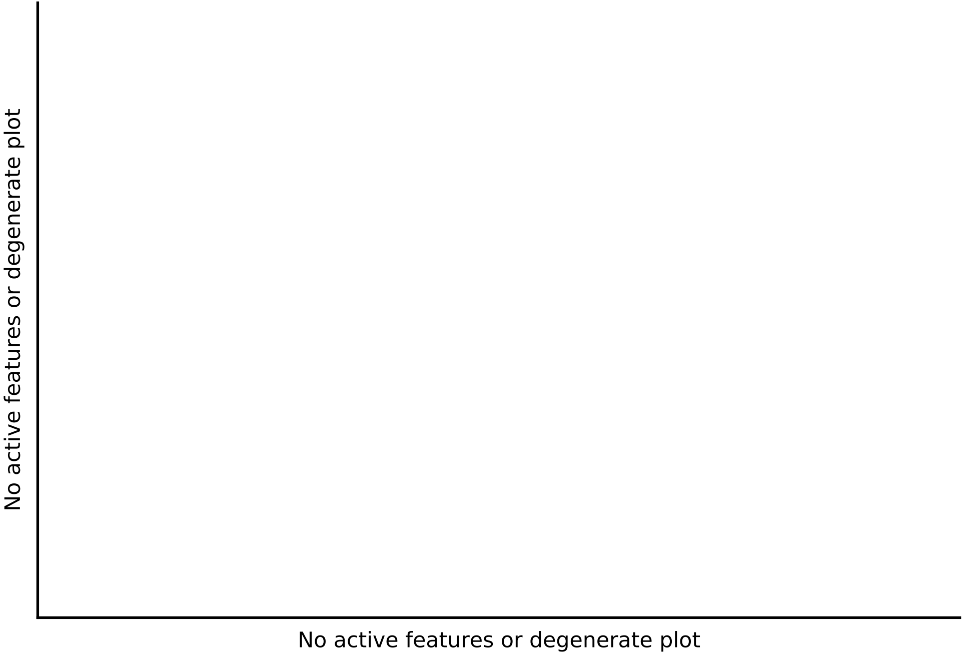

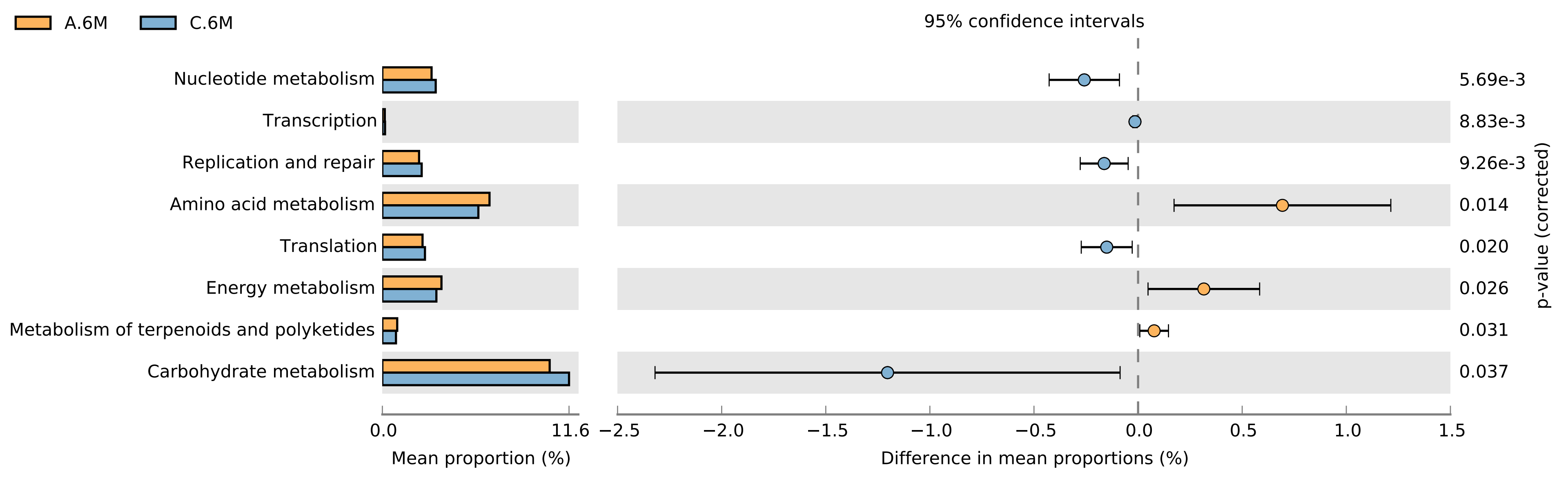

A.6M
C.6M
95% confidence intervals
Nucleotide metabolism
Transcription
Replication and repair
Amino acid metabolism
Translation
Energy metabolism
Metabolism of terpenoids and polyketides
Carbohydrate metabolism
5.69e-3
8.83e-3
9.26e-3
0.014
0.020
0.026
0.031
0.037
p-value (corrected)
0.0
11.6
−2.5
−2.0
−1.5
−1.0
−0.5
0.0
0.5
1.0
1.5
Mean proportion (%)
Difference in mean proportions (%)

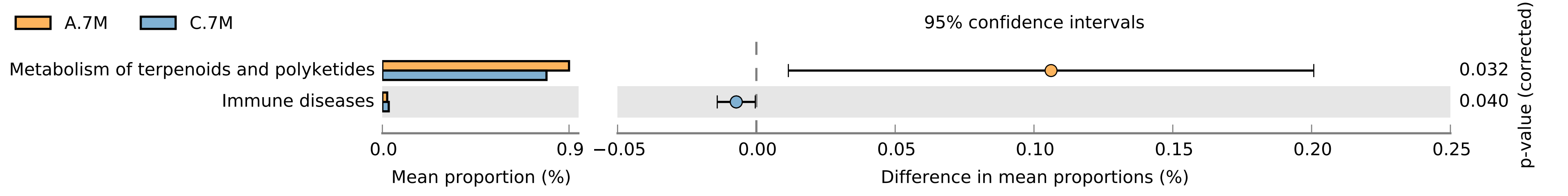
A.7M
C.7M
95% confidence intervals
Metabolism of terpenoids and polyketides
Immune diseases
0.032
0.040
p-value (corrected)
0.0
0.9
−0.05
0.00
0.05
0.10
0.15
0.20
0.25
Mean proportion (%)
Difference in mean proportions (%)

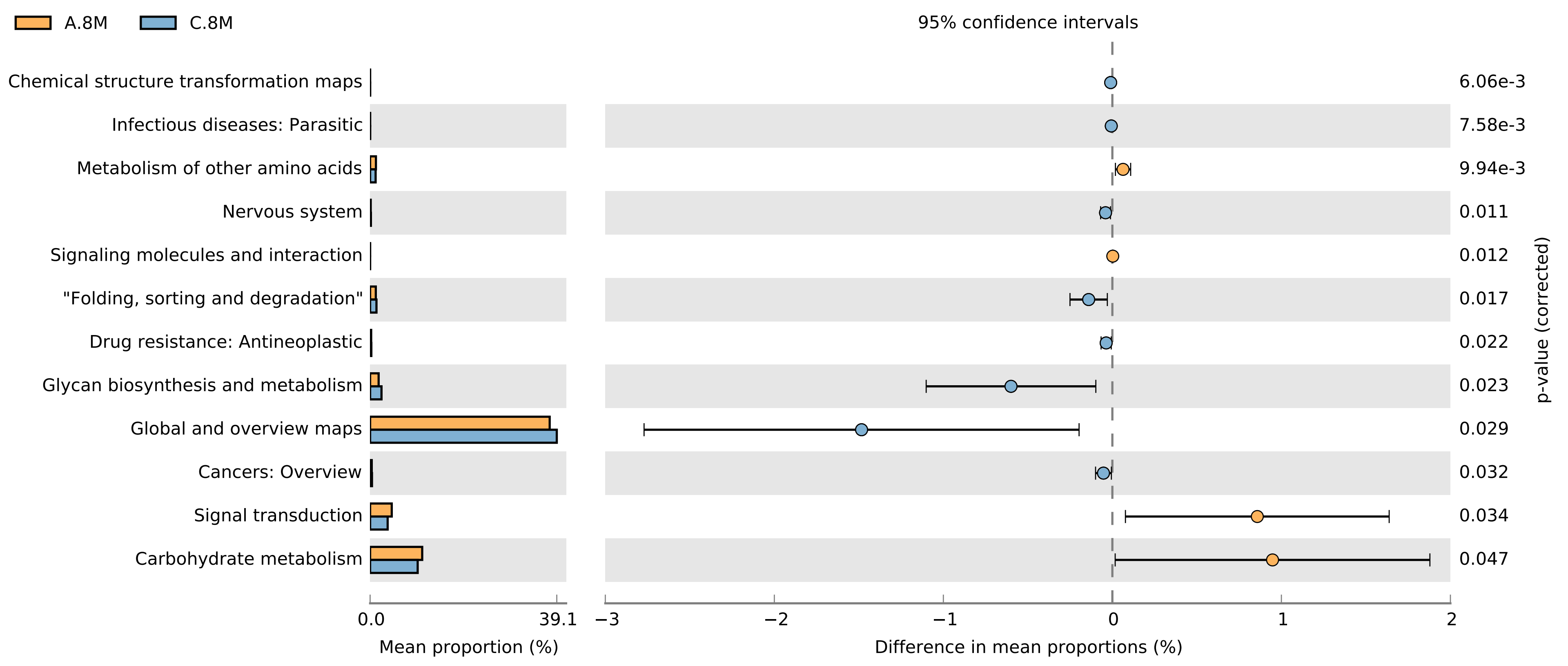

A.8M
C.8M
95% confidence intervals
Chemical structure transformation maps
Infectious diseases: Parasitic
Metabolism of other amino acids
Nervous system
Signaling molecules and interaction
"Folding, sorting and degradation"
Drug resistance: Antineoplastic
Glycan biosynthesis and metabolism
Global and overview maps
Cancers: Overview
Signal transduction
Carbohydrate metabolism
6.06e-3
7.58e-3
9.94e-3
0.011
0.012
0.017
0.022
0.023
0.029
0.032
0.034
0.047
p-value (corrected)
0.0
39.1
Mean proportion (%)
−3
−2
−1
0
1
2
Difference in mean proportions (%)

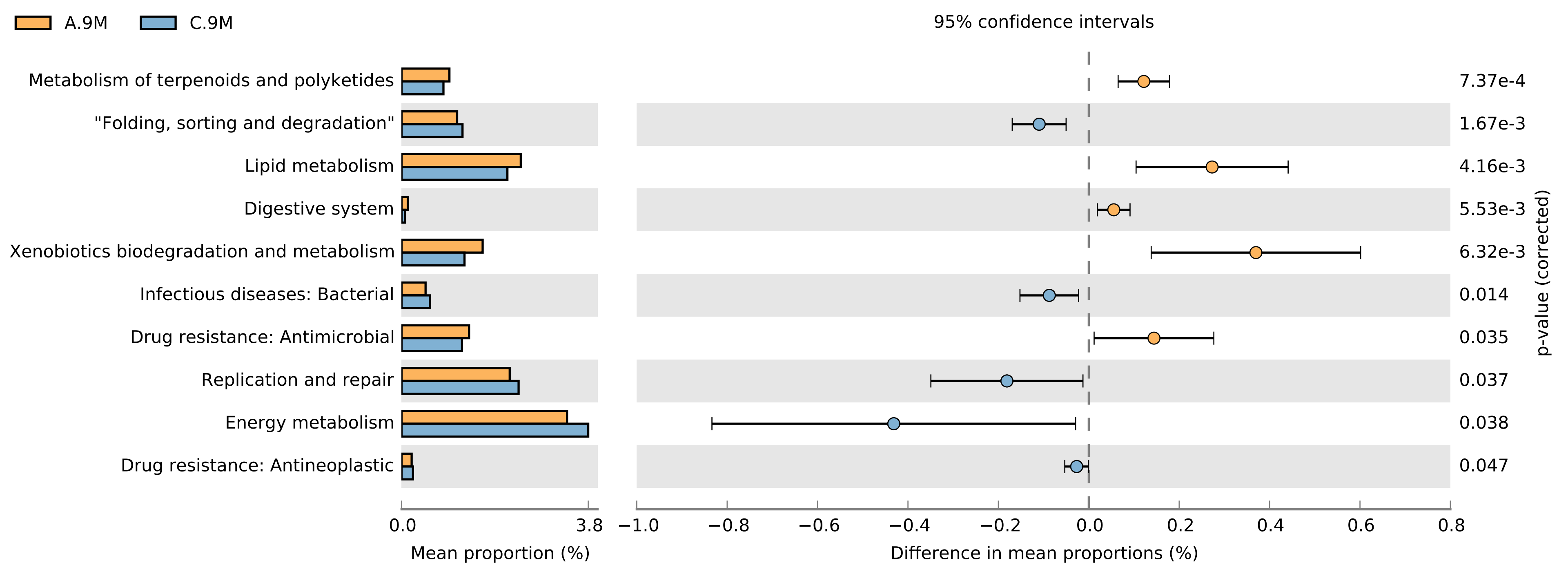
A.9M
C.9M
95% confidence intervals
Metabolism of terpenoids and polyketides
"Folding, sorting and degradation"
Lipid metabolism
Digestive system
Xenobiotics biodegradation and metabolism
Infectious diseases: Bacterial
Drug resistance: Antimicrobial
Replication and repair
Energy metabolism
Drug resistance: Antineoplastic
7.37e-4
1.67e-3
4.16e-3
5.53e-3
6.32e-3
0.014
0.035
0.037
0.038
0.047
p-value (corrected)
0.0
3.8
Mean proportion (%)
−1.0
−0.8
−0.6
−0.4
−0.2
0.0
0.2
0.4
0.6
0.8
Difference in mean proportions (%)

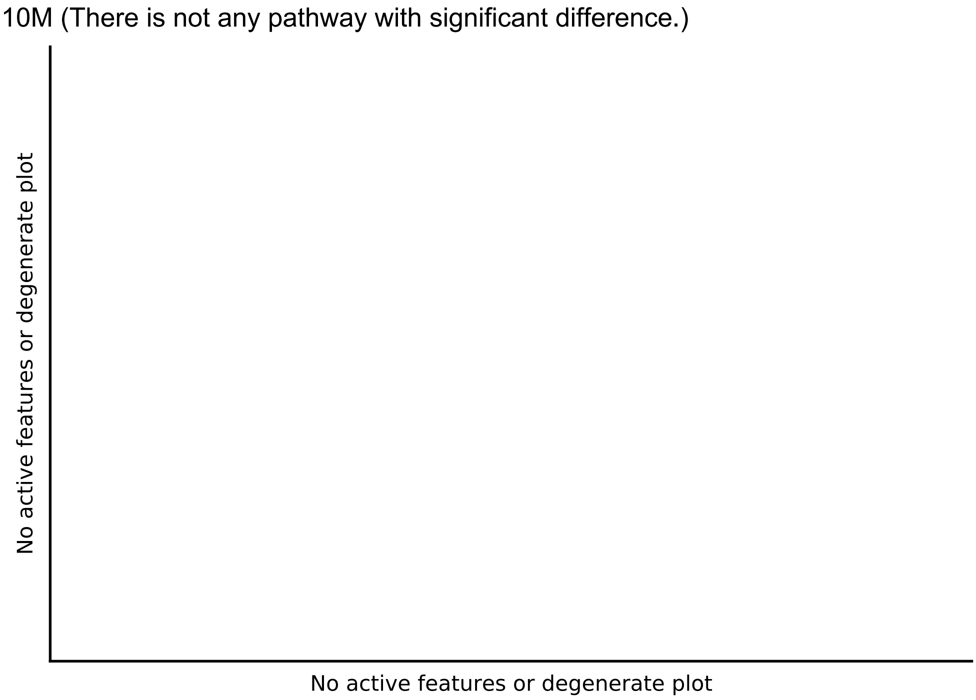
10M (There is not any pathway with significant difference.)
No active features or degenerate plot
No active features or degenerate plot

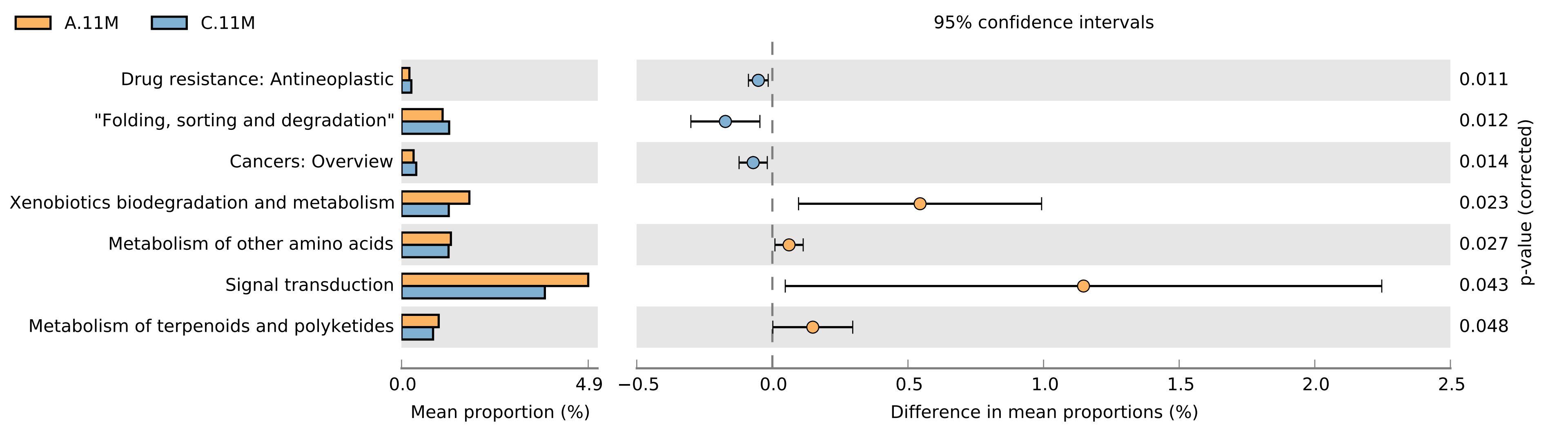
A.11M
C.11M
95% confidence intervals
Drug resistance: Antineoplastic
"Folding, sorting and degradation"
Cancers: Overview
Xenobiotics biodegradation and metabolism
Metabolism of other amino acids
Signal transduction
Metabolism of terpenoids and polyketides
0.011
0.012
0.014
0.023
0.027
0.043
0.048
p-value (corrected)
0.0
4.9
Mean proportion (%)
−0.5
0.0
0.5
1.0
1.5
2.0
2.5
Difference in mean proportions (%)

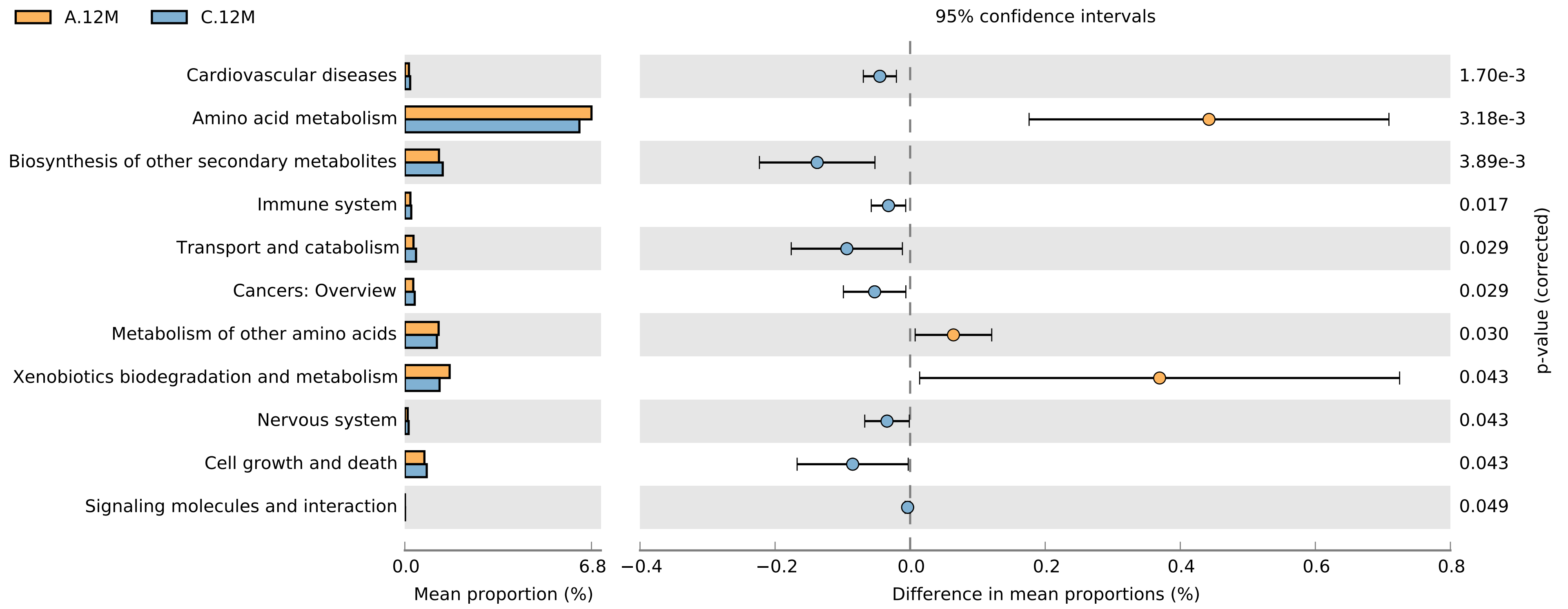
A.12M
C.12M
95% confidence intervals
Cardiovascular diseases
Amino acid metabolism
Biosynthesis of other secondary metabolites
Immune system
Transport and catabolism
Cancers: Overview
Metabolism of other amino acids
Xenobiotics biodegradation and metabolism
Nervous system
Cell growth and death
Signaling molecules and interaction
1.70e-3
3.18e-3
3.89e-3
0.017
0.029
0.029
0.030
0.043
0.043
0.043
0.049
p-value (corrected)
0.0
6.8
Mean proportion (%)
−0.4
−0.2
0.0
0.2
0.4
0.6
0.8
Difference in mean proportions (%)

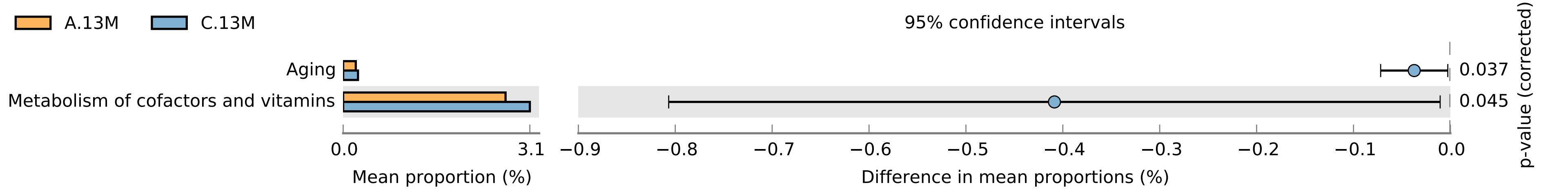

A.13M
C.13M
95% confidence intervals
Aging
Metabolism of cofactors and vitamins
0.037
0.045
p-value (corrected)
0.0
3.1
Mean proportion (%)
−0.9
−0.8
−0.7
−0.6
−0.5
−0.4
−0.3
−0.2
−0.1
0.0
Difference in mean proportions (%)

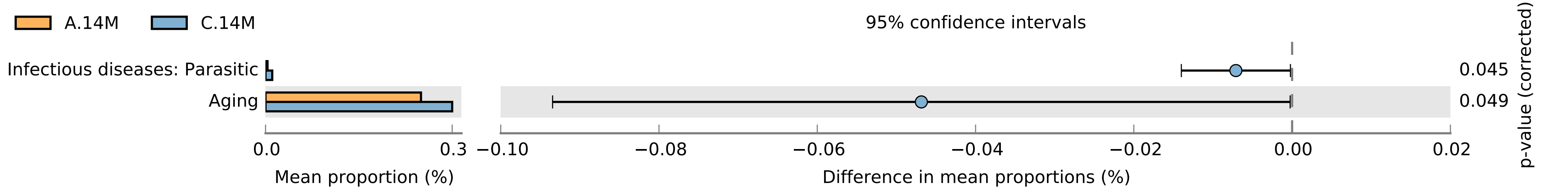

A.14M
C.14M
95% confidence intervals
Infectious diseases: Parasitic
Aging
0.045
0.049
p-value (corrected)
0.0
0.3
Mean proportion (%)
−0.10
−0.08
−0.06
−0.04
−0.02
0.00
0.02
Difference in mean proportions (%)
