# Supplementary material for: Short-term exposure to antibiotics begets long-term disturbance in gut microbial metabolism and molecular ecological networks
Source: Microbiome. 2024 May 7;12:80. doi: 10.1186/s40168-024-01795-z (PMC11075301; doi:10.1186/s40168-024-01795-z)
Supplement: Supplementary file 7 — Additional file 6: Supplementary Figure S11. Functional networks based on the 16S rRNA gene-sequencing data. A and C represent antibiotic group and control, respectively. Pathways and the correlation between pathways are represented by nodes and links, respectively. In each subfigure different subnetworks are represented by different colors. Yet, it does not mean that in groups A and C the same colour indicate the same subnetwork. The unclustered pathways are displayed in the grid layout. [file 40168_2024_1795_MOESM6_ESM.pdf]

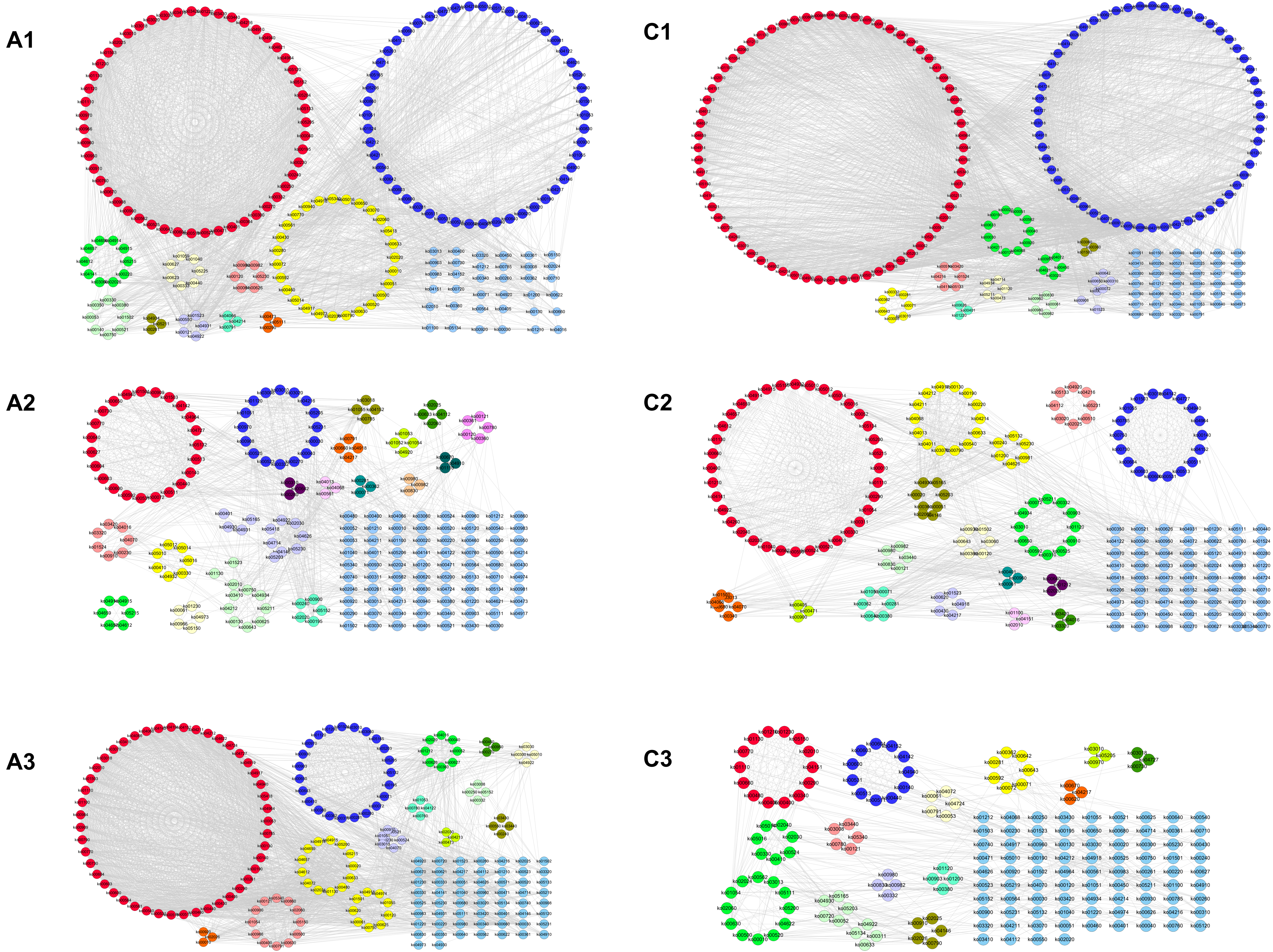
A1
C1
A2
C2
A3
C3

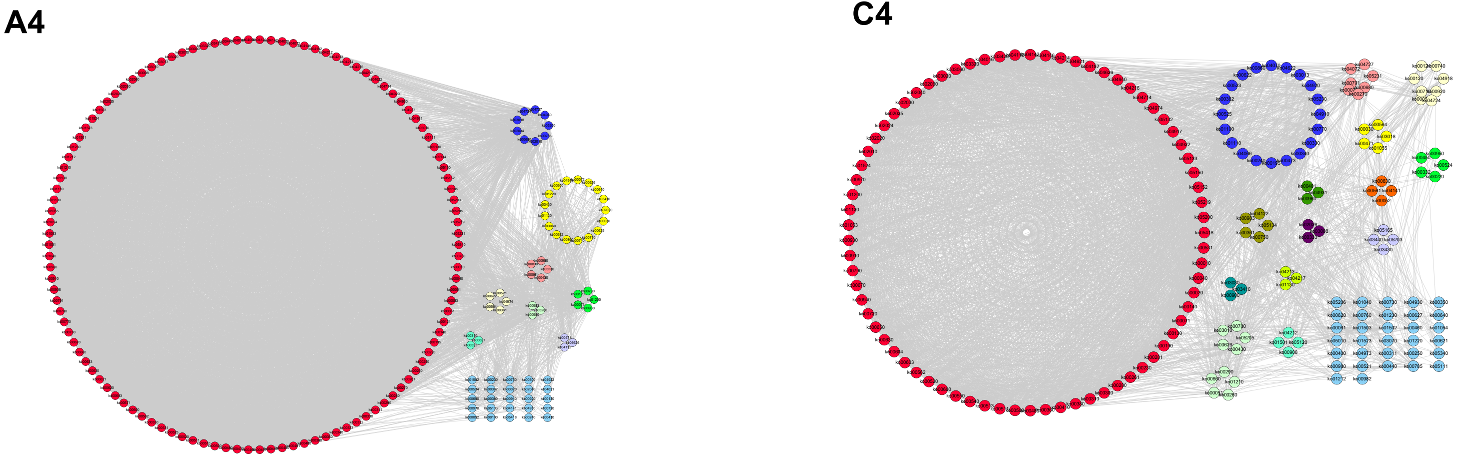
A4
C4

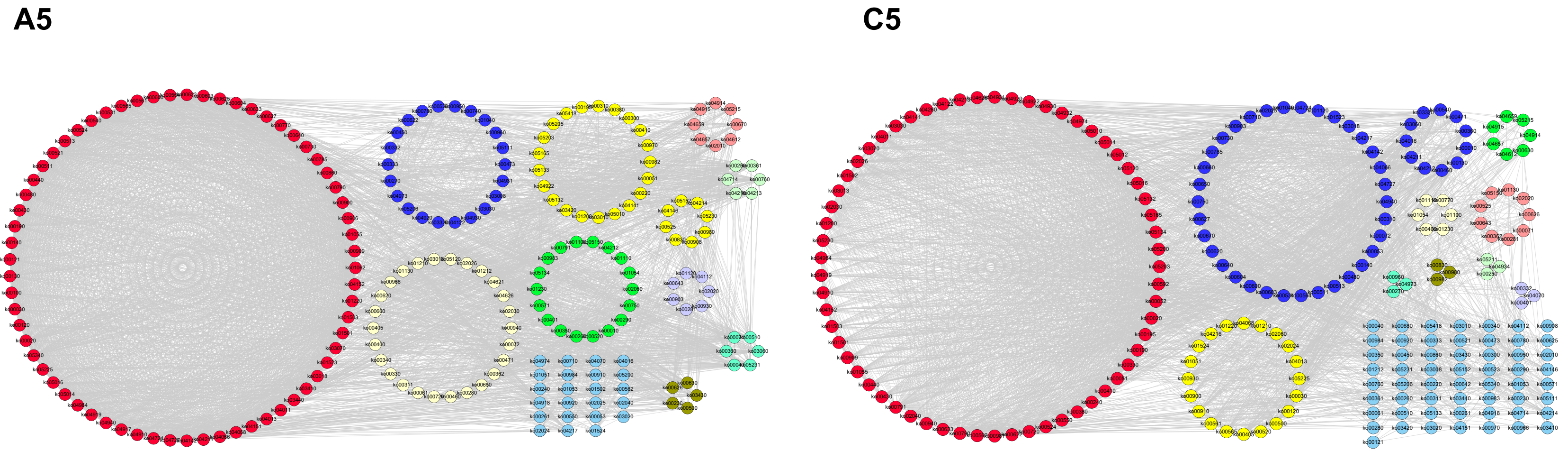
A5
C5

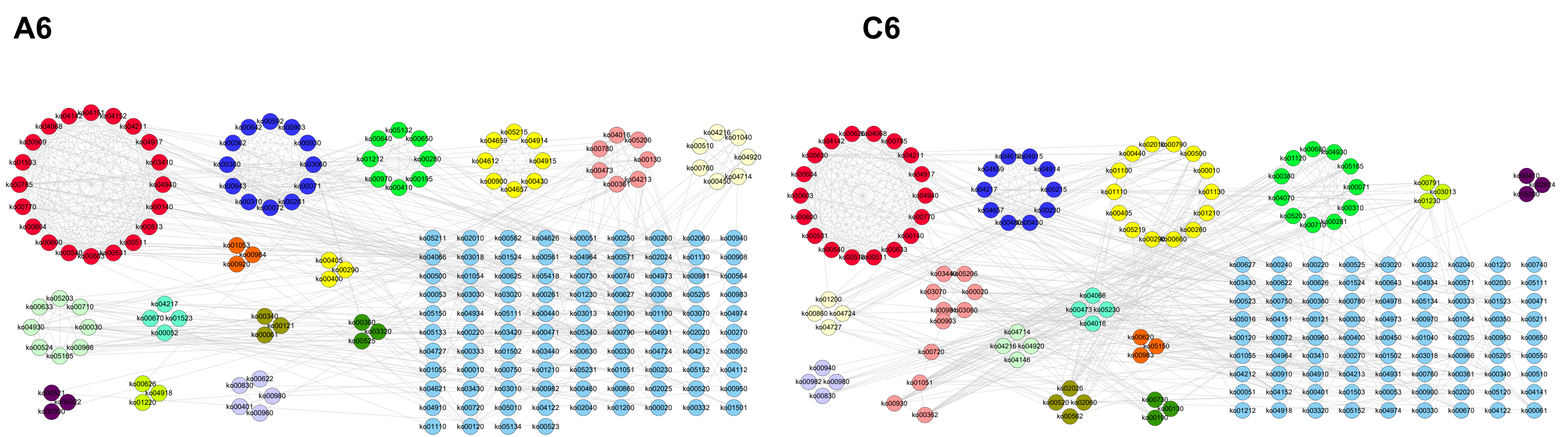
A6
C6

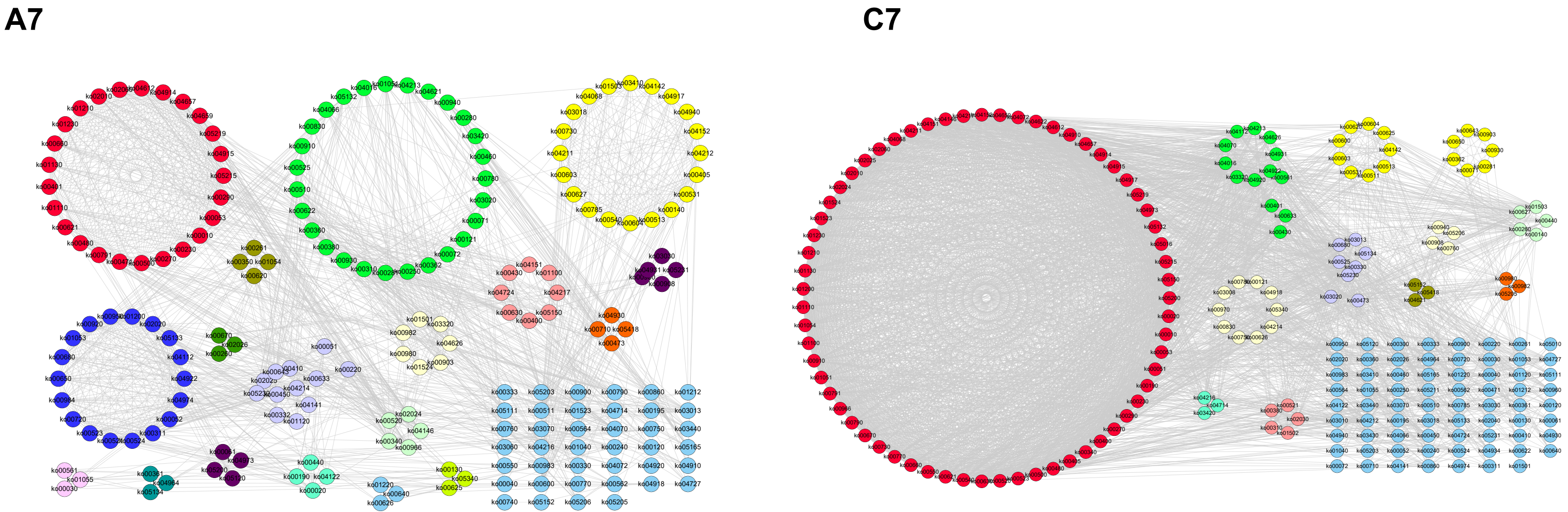
A7
C7

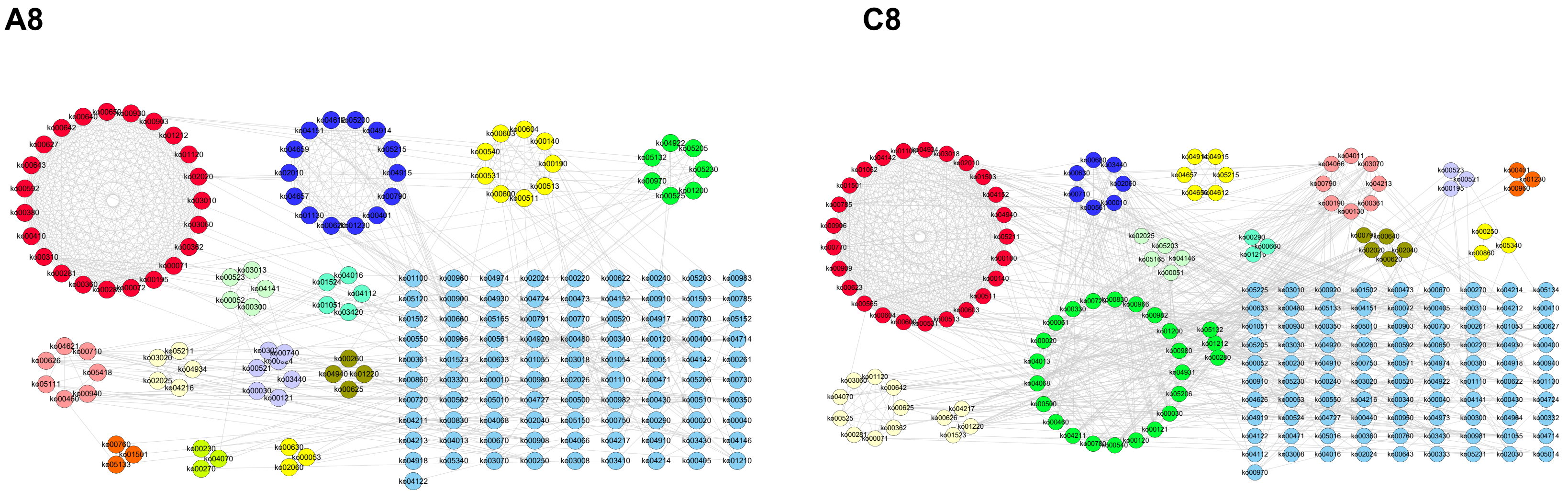
A8
C8

A9

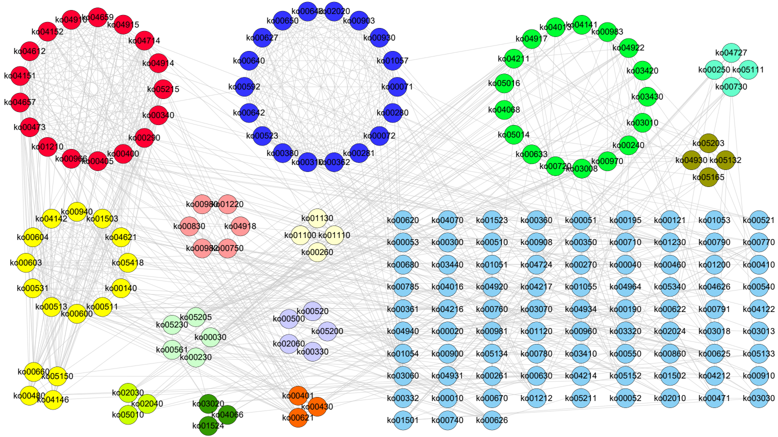
ko04910 ko04659 ko04915 ko04152 ko04714 ko04612 ko04914 ko04151 ko05215 ko04657 ko00340 ko00473 ko00290 ko01210 ko00400 ko00965 ko00405
ko00648 ko02020 ko00650 ko00903 ko00627 ko00930 ko01057 ko00640 ko00592 ko00071 ko00642 ko00280 ko00523 ko00072 ko00380 ko00281 ko00310 ko00362
ko04013 ko04141 ko04917 ko00983 ko04922 ko04211 ko03420 ko05016 ko03430 ko04068 ko03010 ko05014 ko00240 ko00633 ko00970 ko00720 ko03008
ko04727 ko00250 ko05111 ko00730
ko05203 ko04930 ko05132 ko05165
ko00940 ko04142 ko01503 ko00604 ko04621 ko00603 ko05418 ko00531 ko00140 ko00513 ko00511 ko00600 ko00660 ko05150 ko00480 ko04146
ko00980 ko01220 ko00830 ko04918 ko00982 ko00750
ko01130 ko01100 ko01110 ko00260
ko05205 ko05230 ko00030 ko00561 ko00230
ko00520 ko00500 ko05200 ko02060 ko00330
ko02030 ko02040 ko05010
ko03020 ko04066 ko01524
ko00401 ko00430 ko00621
ko00620 ko04070 ko01523 ko00360 ko00051 ko00195 ko00121 ko01053 ko00521
ko00053 ko00300 ko00510 ko00908 ko00350 ko00710 ko01230 ko00790 ko00770
ko00680 ko03440 ko01051 ko04724 ko00270 ko00040 ko00460 ko01200 ko00410
ko00785 ko04016 ko04920 ko04217 ko01055 ko04964 ko05340 ko04626 ko00540
ko00361 ko04216 ko00760 ko03070 ko04934 ko00190 ko00622 ko00791 ko04122
ko04940 ko00020 ko00981 ko01120 ko00960 ko03320 ko02024 ko03018 ko03013
ko01054 ko00900 ko05134 ko00780 ko03410 ko00550 ko00860 ko00625 ko05133
ko03060 ko04931 ko00261 ko00630 ko04214 ko05152 ko01502 ko04212 ko00910
ko00332 ko00010 ko00670 ko01212 ko05211 ko00052 ko02010 ko00471 ko03030
ko01501 ko00740 ko00626

C9

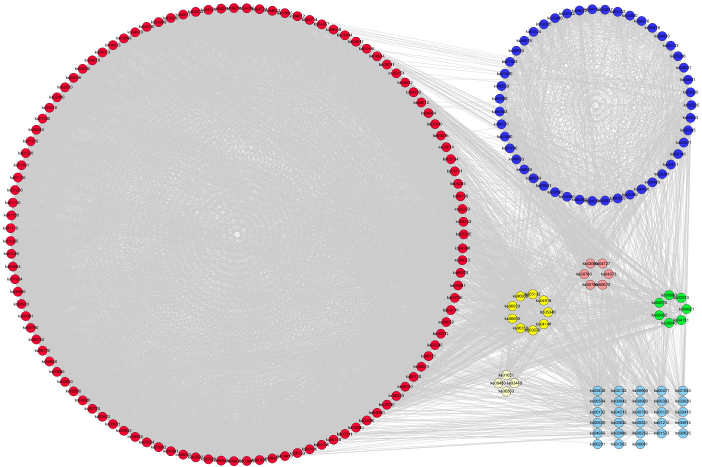

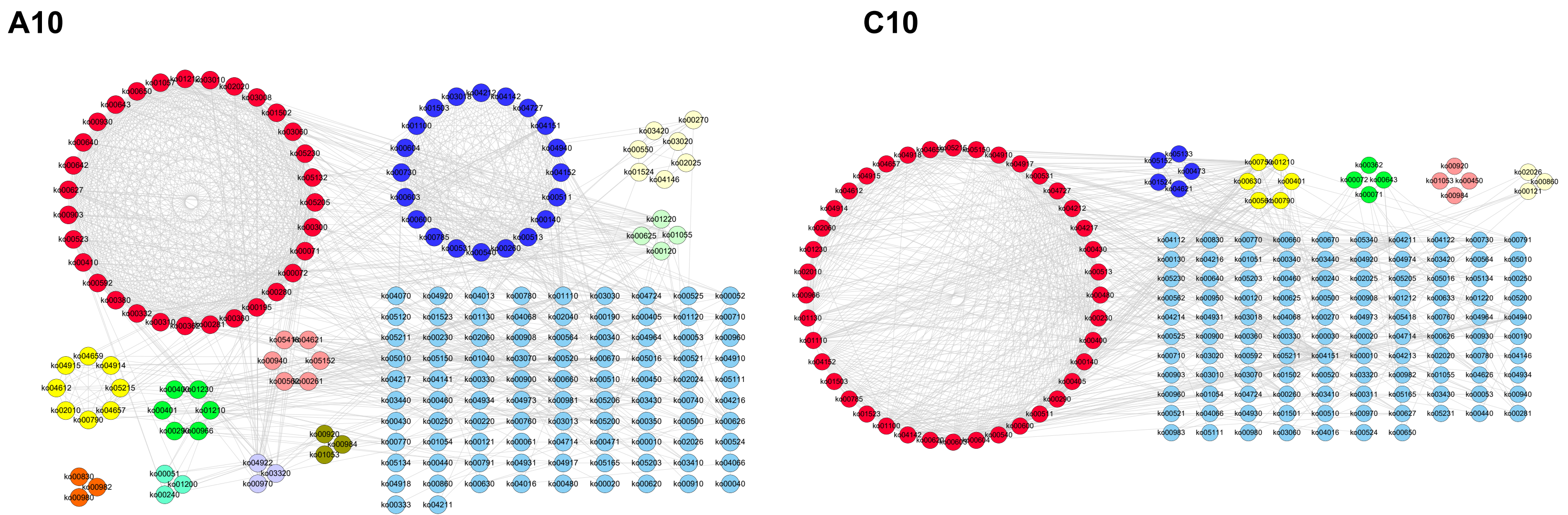
A10
C10

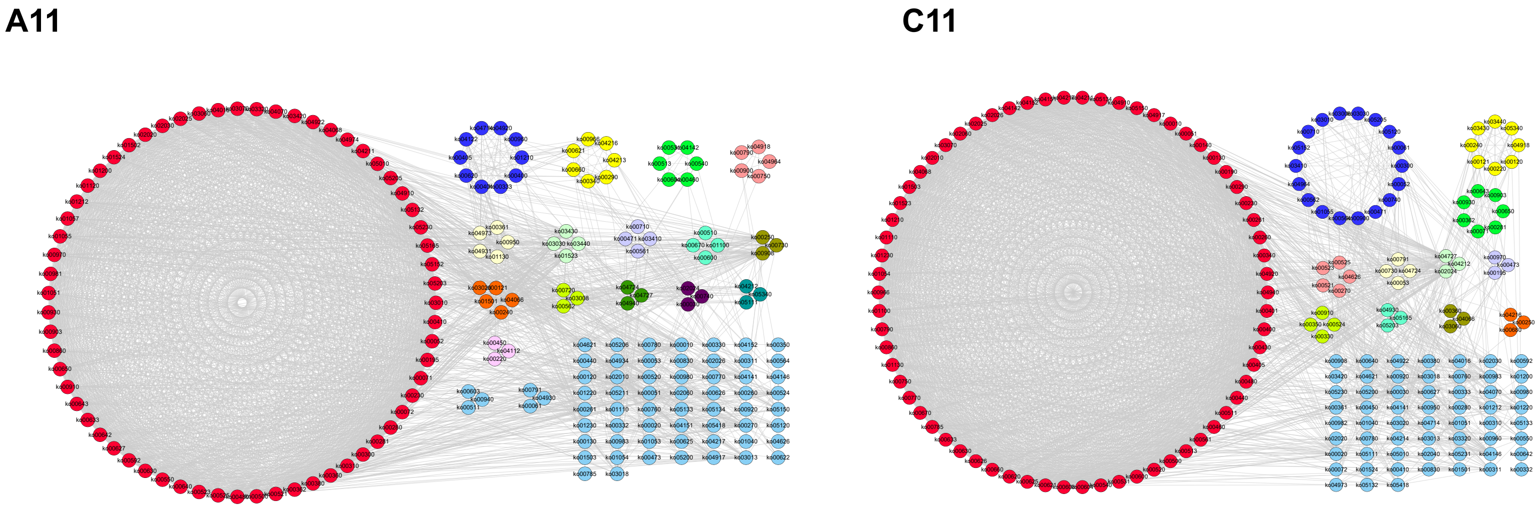
A11
C11

A12

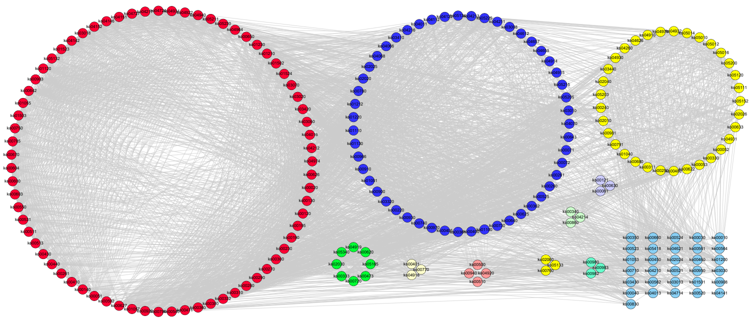

C12

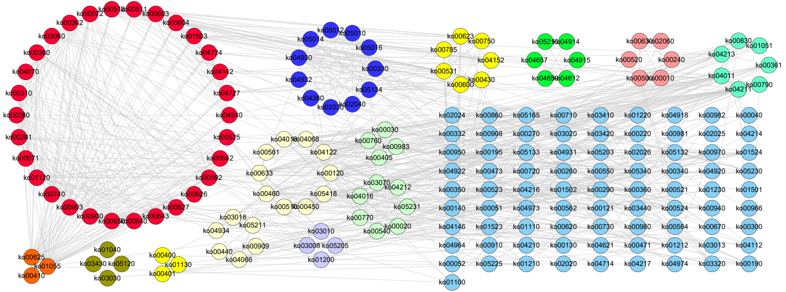
ko00512
ko00511
ko00012
ko00603
ko00362
ko00604
ko03060
ko01503
ko00380
ko04724
ko04070
ko04142
ko00310
ko04727
ko00280
ko04940
ko00281
ko00525
ko00071
ko00642
ko01120
ko00592
ko00740
ko00626
ko00903
ko00627
ko00930
ko00643
ko00650
ko00640
ko05012
ko05010
ko05014
ko05016
ko04930
ko00330
ko04932
ko05134
ko04260
ko02030
ko02040
ko00623
ko00750
ko00785
ko04152
ko00531
ko00600
ko00430
ko05215
ko04914
ko04657
ko04915
ko04650
ko04612
ko00630
ko02060
ko00520
ko00240
ko00500
ko00010
ko00830
ko01051
ko04213
ko00361
ko04011
ko04211
ko00790
ko00030
ko00760
ko00983
ko00405
ko03070
ko04212
ko04016
ko05231
ko00770
ko00020
ko00540
ko04010
ko04068
ko00561
ko04122
ko00633
ko00120
ko00460
ko05418
ko00510
ko00450
ko03018
ko05211
ko04934
ko00909
ko00440
ko04066
ko03010
ko03008
ko05205
ko01200
ko00625
ko01055
ko00410
ko01040
ko03430
ko05120
ko03030
ko00400
ko01130
ko00401
ko02024 ko00860 ko05165 ko00710 ko03410 ko01220 ko04918 ko00982 ko00040
ko00332 ko00908 ko00270 ko03020 ko03420 ko00220 ko00981 ko02025 ko04214
ko00950 ko00195 ko05133 ko04931 ko05203 ko02026 ko05132 ko00970 ko01524
ko04922 ko00473 ko00720 ko00260 ko00550 ko05340 ko00340 ko04920 ko05230
ko00350 ko00523 ko04216 ko01502 ko00290 ko00360 ko00521 ko01230 ko01501
ko00140 ko00051 ko04973 ko00562 ko00121 ko03440 ko00524 ko00940 ko00966
ko04146 ko01523 ko01110 ko00620 ko00730 ko00980 ko00564 ko00670 ko00300
ko04964 ko00910 ko04210 ko00130 ko04621 ko00471 ko01212 ko03013 ko04112
ko00052 ko05225 ko01210 ko02020 ko04714 ko04217 ko04974 ko03320 ko00190
ko01100

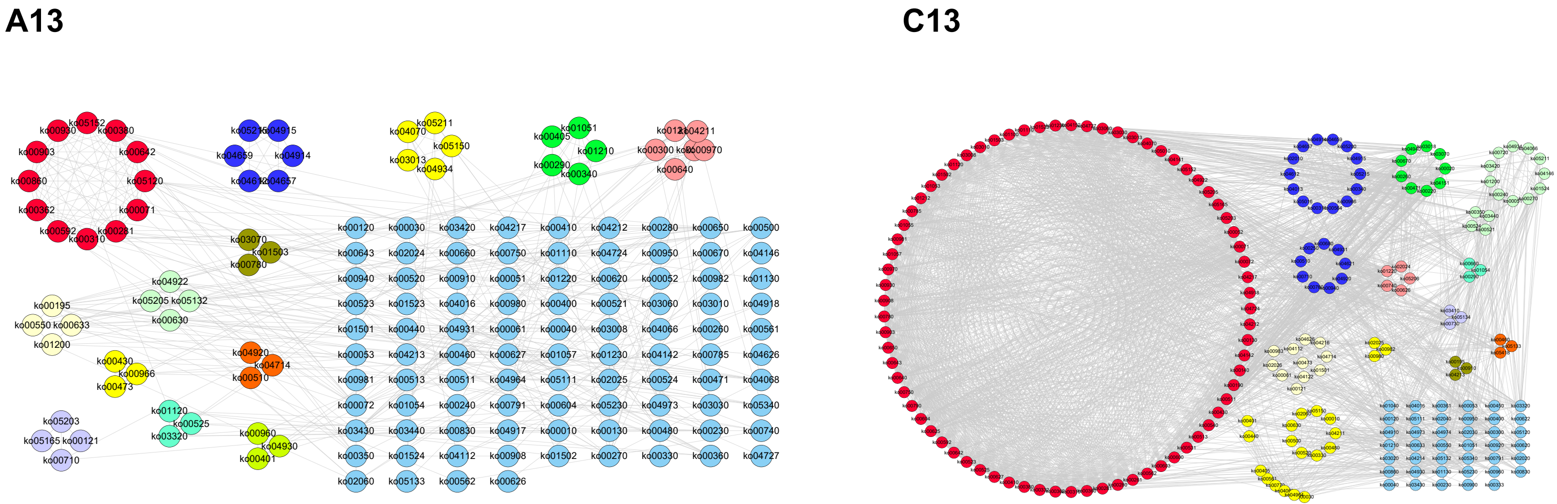

A13
C13
ko05152
ko00930
ko00380
ko00903
ko00642
ko00860
ko05120
ko00362
ko00071
ko00592
ko00281
ko00310
ko04915
ko04659
ko04914
ko04657
ko03070
ko01503
ko00780
ko04922
ko05205
ko05132
ko00630
ko00195
ko00550
ko00633
ko01200
ko00430
ko00966
ko00473
ko04920
ko04714
ko00510
ko05203
ko05165
ko00121
ko00710
ko01120
ko00525
ko03320
ko00960
ko04930
ko00401
ko05211
ko04070
ko05150
ko03013
ko04934
ko01051
ko00405
ko01210
ko00290
ko00340
ko04211
ko00300
ko00970
ko00640
ko00120
ko00030
ko03420
ko04217
ko00410
ko04212
ko00280
ko00650
ko00500
ko00643
ko02024
ko00660
ko00750
ko01110
ko04724
ko00950
ko00670
ko04146
ko00940
ko00520
ko00910
ko00051
ko01220
ko00620
ko00052
ko00982
ko01130
ko00523
ko01523
ko04016
ko00980
ko00400
ko00521
ko03060
ko03010
ko04918
ko01501
ko00440
ko04931
ko00061
ko00040
ko03008
ko04066
ko00260
ko00561
ko00053
ko04213
ko00460
ko00627
ko01057
ko01230
ko04142
ko00785
ko04626
ko00981
ko00513
ko00511
ko04964
ko05111
ko02025
ko00524
ko00471
ko04068
ko00072
ko01054
ko00240
ko00791
ko00604
ko05230
ko04973
ko03030
ko05340
ko03430
ko03440
ko00830
ko04917
ko00010
ko00130
ko00480
ko00230
ko00740
ko00350
ko01524
ko04112
ko00908
ko01502
ko00270
ko00330
ko00360
ko04727
ko02060
ko05133
ko00562
ko00626

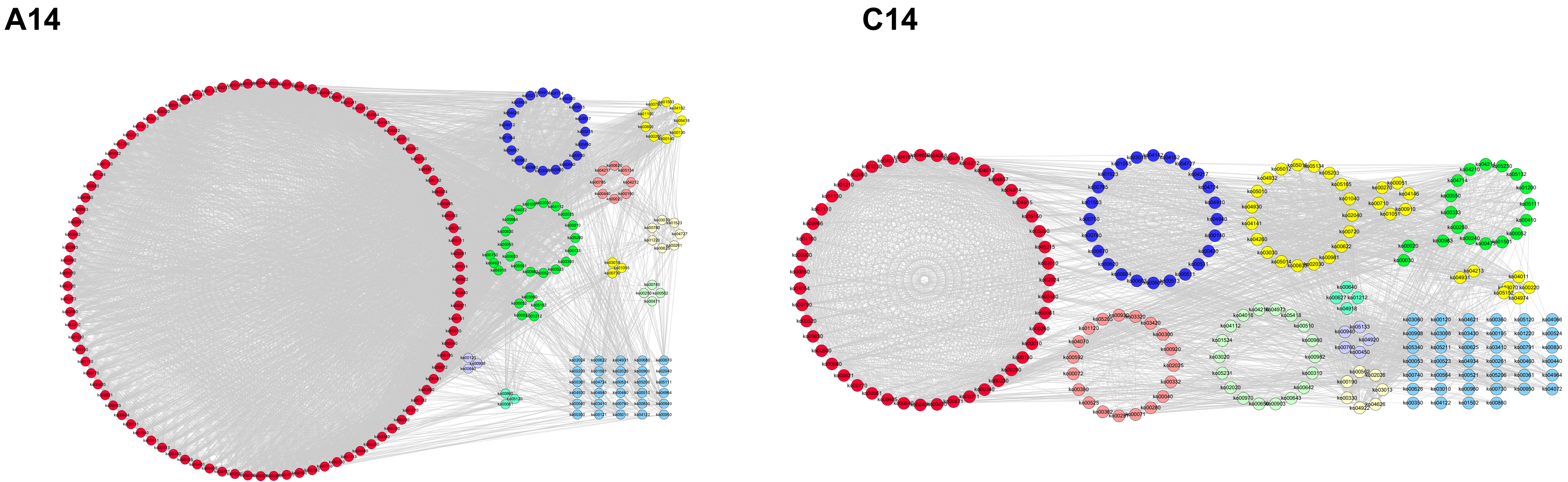
A14
C14
