# Supplementary material for: Short-term exposure to antibiotics begets long-term disturbance in gut microbial metabolism and molecular ecological networks
Source: Microbiome. 2024 May 7;12:80. doi: 10.1186/s40168-024-01795-z (PMC11075301; doi:10.1186/s40168-024-01795-z)
Supplement: Supplementary file 17 — Additional file 16: Table S8. The number of identified metabolites and pathways in metabolomics analysis. [file 40168_2024_1795_MOESM16_ESM.docx]

**Table S8 The number of identified metabolites and pathways in metabolomics analysis**

| **Month** | **The number of metabolites** | **Up-regulated metabolites in antibiotic group** | **Down-regulated metabolites in antibiotic group** | **The number of annotated pathways** |
| --- | --- | --- | --- | --- |
| 0.5M | 346 | 36 | 71 | 33 |
| 1M | 346 | 49 | 44 | 32 |
| 1.5M | 270 | 70 | 37 | 32 |
| 2M | 346 | 32 | 48 | 28 |
| 2.5M | 424 | 68 | 7 | 32 |
| 3M | 346 | 29 | 29 | 22 |
| 3.5M | 183 | 5 | 64 | 27 |
| 4M | 346 | 12 | 37 | 21 |
| 4.5M | 348 | 2 | 77 | 26 |
| 5M | 346 | 13 | 60 | 29 |
| 5.5M | 305 | 32 | 16 | 19 |
| 6M | 394 | 6 | 19 | 17 |
| 7M | 325 | 50 | 36 | 34 |
| 7.5M | 390 | 2 | 125 | 32 |
| 8M | 397 | 48 | 15 | 28 |
| 8.5M | 368 | 51 | 21 | 32 |
| 9M | 391 | 22 | 5 | 18 |
| 10M | 390 | 40 | 27 | 24 |
| 11M | 390 | 5 | 22 | 24 |
| 12M | 365 | 23 | 49 | 23 |

Note: M is short for month.
