# Supplementary material for: Short-term exposure to antibiotics begets long-term disturbance in gut microbial metabolism and molecular ecological networks
Source: Microbiome. 2024 May 7;12:80. doi: 10.1186/s40168-024-01795-z (PMC11075301; doi:10.1186/s40168-024-01795-z)
Supplement: Supplementary file 18 — Additional file 17: Table S9. The discussed pathways with significant difference between two groups. [file 40168_2024_1795_MOESM17_ESM.docx]

**Table S9 The discussed pathways with significant difference between two groups**

| **Month** | | | **1M** | **2M** | **3M** | **4M** | **5M** | **6M** | **7M** | **8M** | **9M** | **10M** | **11M** | **12M** | **13M** | **14M** |
| --- | --- | --- | --- | --- | --- | --- | --- | --- | --- | --- | --- | --- | --- | --- | --- | --- |
| The total number of pathways with significant difference | | | 118 | 50 | 12 | 17 | 4 | 47 | 13 | 77 | 69 | 3 | 62 | 73 | 11 | 7 |
| The number of dominant pathways in antibiotic group | | | 38 | 6 | 3 | 9 | 0 | 19 | 4 | 24 | 43 | 1 | 34 | 24 | 1 | 2 |
| The number of dominant pathways in control group | | | 80 | 44 | 9 | 8 | 4 | 28 | 9 | 53 | 26 | 2 | 28 | 49 | 10 | 5 |
| The number of dominant pathways related to antibiotic biosynthesis | Antibiotic group | | 2 | 1 | 1 | 1 | 0 | 0 | 0 | 1 | 1 | 1 | 2 | 0 | 0 | 0 |
|  | Control | | 8 | 1 | 1 | 1 | 0 | 1 | 1 | 3 | 1 | 0 | 2 | 4 | 1 | 0 |
| The number of dominant pathways related to degradation of organic toxicants | Antibiotic group | | 6 | 0 | 0 | 0 | 0 | 1 | 0 | 2 | 6 | 0 | 8 | 4 | 0 | 0 |
|  | Control | | 1 | 2 | 0 | 0 | 0 | 1 | 1 | 1 | 1 | 0 | 2 | 3 | 0 | 0 |
| The number of dominant pathways related to drug resistance | Antibiotic group | Drug resistance: antimicrobial | 0 | 0 | 0 | 0 | 0 | 0 | 0 | 0 | 2 | 0 | 1 | 0 | 0 | 0 |
|  |  | Drug resistance: antineoplastic | 0 | 0 | 0 | 0 | 0 | 0 | 0 | 0 | 0 | 0 | 0 | 0 | 0 | 0 |
|  | Control | Drug resistance: antimicrobial | 0 | 0 | 0 | 1 | 0 | 0 | 0 | 0 | 0 | 0 | 1 | 1 | 1 | 0 |
|  |  | Drug resistance: antineoplastic | 1 | 0 | 0 | 0 | 0 | 0 | 0 | 1 | 1 | 0 | 2 | 0 | 0 | 0 |

Note: 1M means the 1^st^ month, and so on.
